# Real-world study: Assessing the impact of hemolysis on 48 biochemical and immunological analytes through big data analysis and its feasibility validation

**DOI:** 10.1371/journal.pone.0340265

**Published:** 2026-01-23

**Authors:** Chaochao Ma, Xiaoqi Li, Wei Luo, Lian Hou, Dandan Sun, Li Liu, Xin Liu, Ying Zhang, Jingrong Xu, Ling Qiu, Liangyu Xia

**Affiliations:** 1 Department of Laboratory Medicine, Peking Union Medical College Hospital, Peking Union Medical College and Chinese Academy of Medical Science, Beijing, People's Republic of China; 2 Department of Occupational and Environmental Health Sciences, School of Public Health, Peking University, Beijing, China; 3 State Key Laboratory of Complex Severe and Rare Diseases, Peking Union Medical College Hospital, Peking Union Medical College and Chinese Academy of Medical Science, Beijing, People’s Republic of China; Versiti Blood Research Institute, UNITED STATES OF AMERICA

## Abstract

**Background:**

This research utilizes clinical laboratory real-world data to explore the influence of in vitro hemolysis on 48 biochemical and immunological analytes, aiming to propose and validate the methods and tools based on big data for analysis of the impact of hemolysis on laboratory analytes.

**Methods:**

This research initially employs univariate analysis to display the levels and distribution of 48 analytes across different H-index groups. Subsequently, it utilizes quantile regression models to analyze the impact of hemolysis on laboratory analytes, adjusting for age, gender, patient type, and PVD, with the magnitude of impact described using β values and 95% CIs, visualized through error bar graphs. Finally, the study compares its results with those obtained from homogenized experimental research using the same testing platforms and hemolysis assessment methods, validating the feasibility of conducting research based on big data.

**Results:**

Adjusting for gender, age, patient type, and PVD, hemolysis showed a significant positive interference on ALT, Alb, TBil, GGT, AST, CK, LD, K, P, Mg, and FFA(P < 0.001)., and a significant negative interference on DBil, Na, Cl, TCO2, and Cr (P < 0.001). High hemolysis levels also negatively interfere UA, PA, and GA. No consistent pattern of significance was observed for other analytes. Our multivariate analysis, when compared to experimental data, revealed a 93.0% concordance, with discrepancies noted in GGT, ALP, and RF.

**Conclusions:**

The impact of hemolysis on laboratory analytes can be effectively evaluated through comprehensive big data analysis, demonstrating a level of consistency comparable to that of homogeneous experimental research.

## 1. Introduction

Hemolysis, the rupture of erythrocyte and other blood cell membranes leading to the release of intracellular contents into serum or plasma, occurs both in vivo and in vitro [1,2]. In vivo hemolysis precedes blood collection, often resulting from pathological conditions warranting further investigation, such as infections by hemolytic Gram-positive bacteria or complications from artificial heart valves and hereditary erythrocyte disorders. In contrast, in vitro hemolysis, the most prevalent pre-analytical error accounting for 40–70% of global clinical chemistry sample rejections [3,4], arises from improper handling, transportation, and storage of samples.

Hemolysis can interfere with laboratory test results, leading to significant disruptions in clinical decision-making processes. The mechanisms of interference are multifaceted, including spectrophotometric interference from released hemoglobin, the interaction of intracellular components with analytes, sample dilution effects, and chemical interference from substances like free hemoglobin [5]. The extent of interference varies with the hemolysis level and may depend on the assay method, necessitating careful management of hemolyzed samples in laboratory measurements.

Previous research has shown that the impact of hemolysis on laboratory analytes significantly depends on the assay method and instrumentation used [6,7]. Thus, clinical laboratories must evaluate the influence of hemolysis on analytes based on their specific methodologies and equipment, including those assessing hemolysis levels, to develop appropriate management strategies for hemolyzed samples. However, experimental evaluation of hemolysis [8,9] effects is often laborious, time-consuming, and costly, requiring significant resources for extensive assessments across different platforms and methods. With advancements in computing power and the development of programming languages like R and Python, coupled with the training of data science talent in clinical laboratories, mining real-world data [10,11] for evidence to support clinical decisions [12,13] and laboratory management has become feasible. Accordingly, this study utilizes real-world big data from clinical laboratories, using R for data cleaning and annotation. We employed non-parametric regression models to analyze the impact and magnitude of in vitro hemolysis on 48 biochemical and immunological analytes. By integrating sampling error and model visualization concepts, this research aims to demonstrate the effects of in vitro hemolysis, comparing results from big data analysis with experimental outcomes to verify the feasibility of this approach. This study not only provides evidence on the extent of in vitro hemolysis’s impact on 48 laboratory analytes but also offers a real-world data analysis approach for assessing the effects of in vitro hemolysis on laboratory analytes, with open-source code serving as a valuable tool for laboratories conducting related analyses.

## 2. Method and materials

### 2.1. Study design and approach

This study was conducted based on real-world data, utilizing patient data from visits to Peking Union Medical College Hospital between September 1, 2016, and December 31, 2023. Data was accessed between January and February 2024. The research process is divided into six parts:

1. Obtaining a total data subset from the data repository according to our inclusion and exclusion criteria.
2. Developing a data cleaning scheme and executing data cleaning.
3. Describing the basic information of the dataset, including sample size, gender ratio, patient age level, and the proportion of patient sources.
4. Conducting univariate analyses to assess the impact of different degrees of hemolysis on 48 biochemical and immunological analytes.
5. Performing multivariate analyses to evaluate the impact of different degrees of hemolysis on 48 biochemical and immunological analytes, with adjustments made for influencing factors using models.
6. Comparing with similar clinical research results from our laboratory in the past to evaluate the feasibility of analyzing the impact of hemolysis on laboratory tests based on real-world data analysis.

### 2.2. Data inclusion and exclusion

**Fig 1:**
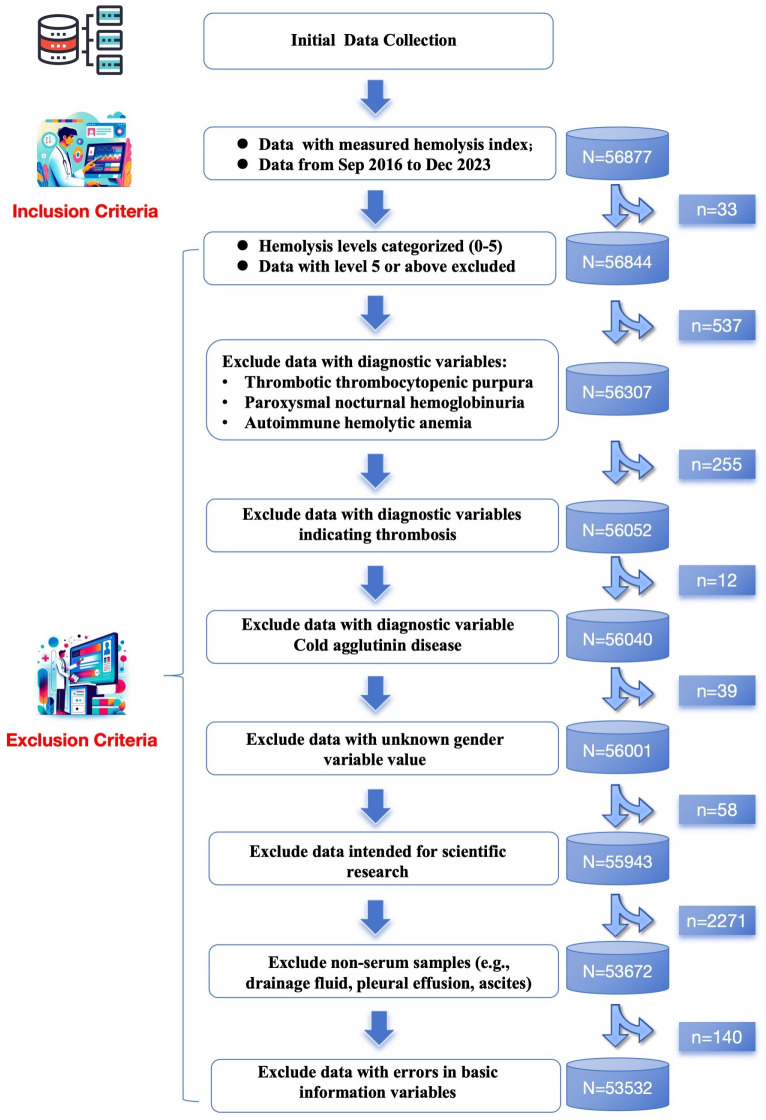
Flowchart of the study process.

Data were selected from the total data repository according to the following inclusion and exclusion criteria, establishing a data subset as illustrated in **Fig 1**.

#### 2.2.1. Inclusion criteria.

Data with a measured hemolysis index and collected between September 2016 and December 2023 were included to ensure consistency in the testing system during this period.

#### 2.2.2. Exclusion criteria.

Hemolysis levels were treated as ordinal categorical variables and divided into six grades (0–5). Records with a hemolysis level of 5 or above, representing extremely severe hemolysis with very few samples, were excluded. In addition, data associated with diagnostic variables such as thrombotic thrombocytopenic purpura, paroxysmal nocturnal hemoglobinuria, autoimmune hemolytic anemia, thrombosis, or cold agglutinin disease were excluded. Samples with an unknown gender variable, data used exclusively for research purposes, and non-serum specimens such as drainage fluid, pleural effusion, and ascites were also excluded. Finally, any records containing errors in basic information variables were removed.

### 2.3. Data cleaning process

The data cleaning procedures and details for this research are outlined below:

1. Due to the treatment of severely hemolyzed samples as non-qualified, some patients have multiple test results, which can affect the construction of the model. Therefore, only the first test result for the same patient was retained to ensure consistency and reliability in the dataset.
2. Exclusion of Missing and Null Values: Entries with missing or null values in their test result variables were removed from the dataset to improve data quality and analysis accuracy.
3. Patient Sample Source Coding: Outpatient samples were coded as 0, whereas inpatient samples were coded as 1, facilitating the differentiation of patient origin in the analysis.
4. Gender Encoding: Male participants were encoded with a value of 1, and female participants were encoded with a value of 0, standardizing the representation of gender across the dataset.
5. Age Standardization: The process involved standardizing age data by eliminating units and uniformly converting age to years, discarding any other units. This step ensures that age data is consistent and comparable across the dataset.
6. To standardize the variable Patient’s Department of Visit (PDV), if a patient visits the Pediatrics department, assign a value of 1, for Endocrinology-related departments assign 2, for Nephrology-related departments assign 3, for Emergency department assign 4, for Hematology-related departments assign 5, for Gynecology or Obstetrics-related departments assign 6, and assign 7 for all other departments.

These meticulous data cleaning steps were crucial to ensuring the dataset’s accuracy and usability, rendering it suitable for the intended analyses.

### 2.4. Instruments and methods

This research employed the AU5800 automatic biochemical analyzer (Beckman Coulter, USA) for conducting 45 out of the 48 analytical tests. These tests include Alanine Aminotransferase (ALT), Total Protein (TP), Albumin (Alb), Total Bilirubin (TBil), Direct Bilirubin (DBil), Gamma-Glutamyl Transferase (GGT), Alkaline Phosphatase (ALP), Aspartate Aminotransferase (AST), Total Bile Acid (TBA), Creatine Kinase (CK), Lactate Dehydrogenase (LD), Cholinesterase (ChE), Potassium (K), Sodium (Na), Chloride (Cl), Total Carbon Dioxide (TCO2), Calcium (Ca), Urea Nitrogen (Urea), Glucose (Glu), Uric Acid (UA), Phosphorus (P), Total Cholesterol (TC), Triglycerides (TG), High-Density Lipoprotein Cholesterol (HDL_C), Low-Density Lipoprotein Cholesterol (LDL_C), Apolipoprotein A1 (ApoA1), Apolipoprotein B (ApoB), Lipoprotein(a) (Lp(a)), High Sensitivity C-Reactive Protein (hsCRP), Magnesium (Mg), Prealbumin (PA), Rheumatoid Factor (RF), Homocysteine (HCY), Immunoglobulin G (IgG), Immunoglobulin A (IgA), Immunoglobulin M (IgM), Antistreptolysin O (ASO), Cystatin C (CysC), Complement Component 3 (C3), Complement Component 4 (C4), Free Fatty Acids (FFA), Glycated Albumin (GA), Creatinine (Cr(E)), Serum Iron (SI) and Transferrin (TRF).The remaining three tests: Serum Ferritin (SF), Serum folic acid (Sfa), and Vitamin B12 (VB12) were carried out using the UniCel DxI 800 Access Immunoassay System (Beckman Coulter, USA). Blood samples for these analyses were collected in Vacuette blood collection tubes (Greiner Bio-One GmbH, Frickenhausen, Germany), which contain a coagulant to accelerate blood coagulation. Following serum separation, analyses were conducted as per the specified methods. Detailed information on the methods and units employed for these 48 tests is provided in S1 Table in S1 File, ensuring clear and comprehensive documentation of the analytical procedures.

Beckman Coulter instruments equipped with hemolysis index (H-Index) capabilities automatically assess these indices using spectrophotometric methods. The instruments measure the absorbance of light at specific wavelengths that correspond to the optical characteristics of hemoglobin. Based on these measurements, the instrument calculates the indices, which help laboratory personnel identify samples that may require pre-analytical treatment, dilution, or re-collection to ensure accurate test results. In this study, the hemolysis index is classified into six levels: 0, 1, 2, 3, 4, and 5, with level 5 indicating samples with very severe hemolysis. Given that samples with an H-index of 5 are rare and could negatively impact model fitting, they have been excluded from the analysis. The H-index classification used here follows the manufacturer’s LIH serum-index workflow described in the analyzer’s Instructions for Use: a continuous spectrophotometric H-index (derived from predefined hemoglobin-sensitive wavelengths and internally calibrated to hemolysis signal intensity) is converted to ordinal categories via analyzer-specific decision limits, i.e., instrument-defined cut-offs that correspond to increasing degrees of hemolysis and may be locally verified/adjusted by the laboratory to align with method performance and reporting policy.

### 2.5. Quality control

The quality assurance in this study revolves around two critical aspects: quality control during data generation, and quality control during data analysis and programming. During the data generation phase, all 48 tests regularly participated in the inter-laboratory quality assessment program organized by the National Clinical Laboratory Center. Additionally, daily quality control (QC) checks were conducted for these tests to ensure the accuracy and reliability of our test results. Throughout the period covered by the data, there were no changes to the analytical platforms for the analytes. Measurements of specimens were only conducted after passing quality control criteria.

Regarding data analysis and programming, a rigorous code review mechanism was implemented. This mechanism not only ensured that each piece of code was logically annotated but also required that every segment of code be reviewed, checked, and tested by two independent individuals. This dual-review process ensured robust and consistent analysis, thereby minimizing the potential for computational errors or oversights and ensuring that our research findings were based on accurate and reliable data.

### 2.6. Statistical analysis

Data were organized in Microsoft Excel 365 (Microsoft, Redmond, WA, USA) and analyzed using R (version 4.3.1) with relevant packages and scripts. The hemolysis index, categorized into five ordinal levels (0, 1, 2, 3, 4), was grouped according to the H-index output of the instrument, excluding level 5 due to its extremely low frequency and potential to destabilize statistical modeling. Within each group, the Anderson-Darling test was employed to assess the normality of the data. For descriptive statistics, if continuous variables conformed to normality, they were described using mean (standard deviation); otherwise, median (interquartile range) was used. Categorical variables with two categories were described using proportions.

In univariate analysis, if the data across groups were normally distributed, multivariate analysis of variance was utilized for inter-group difference testing. If the data did not meet the normality assumption, the Kruskal-Wallis H test was conducted to examine differences among the H-index groups. For the Kruskal-Wallis H test, the Benjamini-Hochberg (BH) method was applied to control the False Discovery Rate (FDR), thereby balancing the risk of false positives with statistical power. Furthermore, post-hoc pairwise comparisons were performed using the dunn.test package in R, and the Bonferroni correction was used for P-value adjustment to ensure the reliability of the results. Violin plots were used to display and compare data distributions, incorporating features of both box plots and kernel density plots to show statistical information such as median and quartiles, as well as density estimates. Sensitivity analysis was performed by excluding data from individuals under 18 years old to observe if the results changed, considering this analysis does not adjust for other factors’ effects.

For multivariate analysis, considering the impact of outliers on parametric model results, the study employed multivariate quantile regression for modeling, which offers better robustness to outliers. The response variable in the model was the result values of the laboratory tests, with H-index as an explanatory variable. Dummy variables were created for the H-index, using the data with H-index equal to 0 as the reference. The model also adjusted for sex, age, and patient type, PDV. The results were visualized to show changes in the median of the laboratory test results and their 95% confidence intervals with increasing degrees of hemolysis, after adjusting for various factors. If the model issued warnings due to insufficient sample size, a multivariate linear regression model was used under the assumption that model premises were met. To evaluate the dose-dependent impact of hemolysis, we assessed the monotonicity of the adjusted regression coefficients across hemolysis groups (indices 1–4) using a one-sided Spearman’s rank correlation test. To ensure robustness, a penalty correction was applied to the trend P-value based on the number of non-significant coefficients (P > 0.05) in the regression models.

To ensure the robustness and validity of the multivariate regression models, a comprehensive model diagnostic framework was applied. For quantile regression models, Koenker’s goodness-of-fit measure (R^1, a pseudo-R^2 based on the reduction in deviance) was calculated to quantify the proportion of variance explained by the predictors. The Akaike Information Criterion (AIC) was computed to assess model parsimony. Furthermore, to evaluate the generalization performance and rule out overfitting, the Root Mean Square Error (RMSE) was estimated using a 5-fold cross-validation procedure. Finally, residual diagnostics (residuals vs. fitted values plots) were visually inspected for all models to verify the assumptions of linearity and to confirm the presence of heteroscedasticity, thereby justifying the selection of quantile regression over ordinary least squares (OLS) regression for specific analytes.

Data analysis and visualization were performed using R’s for loop, with the ggplot2 package for drawing and the rq function for modeling. The significance level was set at 0.05.

### 2.7. Ethics statements

Ethical clearance was obtained from the Ethics Committee of the Peking Union Medical College Hospital of the Chinese Academy of Medical Sciences, under approval number I-24PJ2489, prior to the commencement of the study. All procedures adhered strictly to the applicable guidelines and regulations. The data were analyzed in an anonymized manner to ensure the privacy and confidentiality of individuals in the database.

## 3. Results

### 3.1. Baseline characteristics

In our detailed evaluation of normality across continuous variables, stratified by four distinct levels of hemolysis, significant deviations from a normal distribution were universally observed across a wide array of analyzed variables (S1 Table in S1 File). The baseline characteristics of the study population were stratified into five groups based on H-index values, ranging from 0 to 4. The distribution of sample sizes, gender ratios, ages, and patient type ratios varied across the H-index categories, reflecting the diversity within the population on these variables (Table 1).

**Table 1:** Baseline characteristics according to H-index categories.

Markers	Sample Size					Gender Ratio					Age					PatientType Ratio				
	H-index_0	H-index_1	H-index_2	H-index_3	H-index_4	H-index_0	H-index_1	H-index_2	H-index_3	H-index_4	H-index_0	H-index_1	H-index_2	H-index_3	H-index_4	H-index_0	H-index_1	H-index_2	H-index_3	H-index_4
**ALT**	18727	18429	4632	870	462	0.6	0.66	0.63	0.65	0.72	47.79 (17.8)	44.78 (21.56)	43.85 (22.52)	39.41 (24.75)	29.89 (26.85)	8.03	5.75	3.69	2.51	1.43
**TP**	16918	16602	4187	790	420	0.61	0.66	0.63	0.64	0.71	48.22 (17.71)	45.16 (21.51)	43.9 (22.63)	39.08 (25.09)	29.04 (26.74)	8.2	5.97	4.03	2.51	1.46
**Alb**	18537	18202	4579	857	456	0.59	0.66	0.63	0.65	0.72	47.85 (17.8)	44.86 (21.56)	43.88 (22.55)	39.24 (24.78)	29.94 (26.92)	7.94	5.65	3.64	2.44	1.44
**TBil**	18448	18112	4557	814	368	0.6	0.66	0.62	0.63	0.64	47.82 (17.8)	44.83 (21.56)	43.89 (22.49)	41.35 (23.66)	36.76 (25.17)	7.95	5.68	3.66	2.75	2.29
**DBil**	18401	18089	4550	829	367	0.6	0.66	0.62	0.64	0.64	47.83 (17.81)	44.82 (21.57)	43.9 (22.49)	40.62 (24.09)	36.78 (25.2)	7.94	5.68	3.67	2.6	2.31
**GGT**	13685	13528	3388	646	355	0.62	0.66	0.66	0.66	0.79	48.9 (17.63)	45.98 (21.39)	44.7 (22.63)	39.85 (25.5)	28.9 (27.71)	6.73	4.97	3.32	2.09	1.11
**ALP**	15525	15346	3835	721	394	0.59	0.66	0.64	0.67	0.77	48.27 (17.79)	45.3 (21.59)	44.25 (22.66)	39.85 (25.12)	29.69 (27.47)	6.74	4.94	3.15	2.13	1.2
**AST**	15448	15319	3676	640	276	0.6	0.67	0.63	0.67	0.72	48.34 (17.77)	45.28 (21.58)	46.12 (21.3)	44.89 (22.2)	42.55 (23.37)	6.71	4.94	3.48	2.83	2.45
**TBA**	13558	13393	3341	638	341	0.61	0.66	0.65	0.66	0.78	48.93 (17.63)	45.98 (21.4)	44.65 (22.65)	39.89 (25.51)	28.89 (27.36)	6.9	5.12	3.45	2.14	1.2
**CK**	1225	1490	323	54	28	0.89	0.91	0.81	0.59	1.15	53.45 (17.91)	44.33 (23.89)	46.91 (22.17)	35.46 (25.96)	41.26 (23.65)	13.41	11.74	6.69	4.4	4.6
**LD**	15320	15184	3646	636	272	0.6	0.67	0.64	0.67	0.72	48.4 (17.78)	45.39 (21.55)	46.21 (21.32)	44.86 (22.34)	42.51 (23.34)	6.66	4.93	3.47	2.74	2.49
**ChE**	13541	13380	3342	639	345	0.61	0.66	0.65	0.66	0.79	48.97 (17.63)	46 (21.4)	44.68 (22.68)	39.76 (25.61)	28.58 (27.49)	6.89	5.12	3.45	2.1	1.14
**K**	16467	16313	3957	633	219	0.63	0.69	0.64	0.67	0.67	48.72 (17.82)	45.83 (21.34)	46.94 (20.96)	45.6 (21.82)	43.65 (22.23)	6.85	4.98	3.49	2.62	2.59
**Na**	16452	16314	3995	678	317	0.63	0.69	0.64	0.67	0.75	48.73 (17.83)	45.82 (21.35)	46.65 (21.23)	44.73 (22.79)	40.72 (24.78)	6.85	4.99	3.42	2.39	2.05
**Cl**	16373	16255	4099	736	401	0.63	0.69	0.65	0.69	0.77	48.7 (17.83)	45.73 (21.42)	45.38 (22.25)	41.06 (24.97)	32.17 (27.53)	6.86	4.96	3.19	1.94	1.19
**TCO2**	16230	16118	4059	728	401	0.62	0.68	0.65	0.69	0.77	48.73 (17.82)	45.78 (21.4)	45.38 (22.25)	41.03 (24.99)	32.06 (27.6)	7.01	5.06	3.25	1.96	1.17
**Ca**	16788	16614	4202	744	415	0.62	0.67	0.65	0.67	0.74	48.55 (17.79)	45.6 (21.44)	45.05 (22.32)	41.02 (24.8)	32.23 (27.48)	7.14	5.17	3.35	2.02	1.22
**Urea**	17759	17591	4482	822	443	0.62	0.67	0.64	0.67	0.74	48.35 (17.83)	45.21 (21.54)	44.35 (22.54)	39.49 (24.97)	30.93 (27.08)	7.68	5.52	3.6	2.33	1.38
**Glu**	16855	16639	4162	739	375	0.63	0.68	0.65	0.68	0.73	48.66 (17.81)	45.96 (21.24)	45.67 (21.97)	42.51 (24.16)	34.25 (27.21)	7.38	5.33	3.53	2.34	1.39
**UA**	16524	16241	4091	737	399	0.63	0.68	0.66	0.69	0.77	48.75 (17.78)	46.04 (21.22)	45.55 (22.17)	41.55 (24.91)	32.31 (27.52)	7.11	5.15	3.32	2.03	1.18
**P**	16696	16428	4132	666	299	0.62	0.67	0.65	0.66	0.7	48.68 (17.79)	45.86 (21.33)	45.39 (22.22)	45.75 (22.1)	44.39 (22.67)	6.86	5.07	3.37	2.58	2.52
**TC**	9282	8882	2049	371	165	0.71	0.75	0.72	0.73	0.74	48.82 (17.51)	46.91 (20.64)	47.31 (20.73)	46.18 (22.29)	43.58 (22.91)	6.79	5.63	3.81	2.37	2.3
**TG**	9364	8992	2070	374	168	0.72	0.75	0.72	0.73	0.75	48.83 (17.5)	46.88 (20.58)	47.22 (20.71)	46.24 (22.28)	43.54 (22.71)	6.7	5.5	3.74	2.37	2.29
**HDL_C**	8644	8342	1893	349	146	0.69	0.75	0.73	0.75	0.72	49.23 (17.47)	47.46 (20.43)	48.09 (20.34)	47.74 (21.52)	46.33 (21.89)	6.43	5.47	3.59	2.36	2.11
**LDL_C**	9034	8650	1989	354	152	0.7	0.74	0.73	0.75	0.73	49.08 (17.45)	47.38 (20.34)	47.96 (20.29)	47.83 (21.46)	46.01 (21.67)	6.69	5.67	3.75	2.4	2.23
**ApoA1**	4676	4484	1028	209	83	0.65	0.73	0.73	0.74	0.93	49.81 (17.19)	47.52 (21.13)	47.76 (21.28)	46.73 (22.84)	46.67 (22.75)	4.02	3.79	2.54	1.65	1.59
**ApoB**	4676	4484	1028	209	83	0.65	0.73	0.73	0.74	0.93	49.81 (17.19)	47.52 (21.13)	47.76 (21.28)	46.73 (22.84)	46.67 (22.75)	4.02	3.79	2.54	1.65	1.59
**Lp(a)**	4676	4481	1028	206	82	0.65	0.73	0.73	0.75	0.91	49.81 (17.18)	47.53 (21.13)	47.76 (21.28)	46.89 (22.78)	46.44 (22.79)	4.02	3.79	2.54	1.71	1.65
**hsCRP**	8863	8417	2104	372	179	0.6	0.65	0.63	0.65	0.67	48.37 (17.09)	46.91 (20.01)	47.19 (20.2)	45.93 (20.94)	44.88 (21.41)	3.99	3.54	2.5	1.88	1.8
**Mg**	4084	4135	911	198	138	0.62	0.68	0.65	0.6	0.79	49.29 (18.08)	45.87 (22.06)	43.92 (24.17)	36.87 (27.62)	19.18 (26.41)	8.43	6.63	3.07	1.44	0.59
**PA**	16854	16563	4171	725	307	0.61	0.66	0.63	0.61	0.62	48.25 (17.71)	45.16 (21.52)	43.9 (22.62)	42.39 (23.34)	39.1 (23.85)	8.14	5.96	4.03	3.17	3.26
**RF**	1138	1085	291	52	22	0.39	0.4	0.37	0.41	0.47	47.04 (16.79)	42.36 (22.02)	40.48 (23.89)	39.67 (24.49)	32.75 (24.73)	6.34	6.14	6.46	4.2	10
**HCY**	1005	1037	271	50	31	0.99	1.23	0.94	1	0.63	53.2 (17.41)	51.42 (20.59)	51.89 (20.44)	52.14 (24.45)	48.08 (21.63)	3.51	3.05	2.04	1.5	1.21
**IgG**	3088	3164	892	138	94	0.44	0.5	0.52	0.42	0.71	44.78 (17.17)	40.51 (20.56)	38.86 (21.97)	34.34 (23.72)	32.56 (24.97)	7.92	6.26	5.07	3.6	3.09
**IgA**	3078	3150	888	138	94	0.44	0.5	0.51	0.42	0.71	44.76 (17.16)	40.5 (20.56)	38.84 (21.94)	34.34 (23.72)	32.56 (24.97)	7.97	6.31	5.04	3.6	3.09
**IgM**	3082	3153	888	138	87	0.44	0.5	0.52	0.42	0.71	44.77 (17.16)	40.5 (20.54)	38.86 (21.95)	34.34 (23.72)	34.81 (24.49)	7.99	6.33	5	3.6	3.14
**ASO**	330	444	125	28	15	0.66	0.55	0.71	0.56	1.14	41.92 (19.32)	31.81 (23.72)	27.11 (25.13)	27.05 (26.84)	13.26 (15.27)	2.71	2.86	3.81	1.8	2.75
**CysC**	218	222	61	15	13	1.83	1.49	1.18	0.88	0.44	40.78 (17.59)	31.12 (23.03)	28.49 (24.26)	21.53 (21.02)	18.05 (24.48)	12.62	10.1	7.71	6.5	1.6
**C3**	2229	2297	680	109	68	0.35	0.35	0.33	0.38	0.45	42.24 (16.53)	37.08 (20.07)	36.4 (21.13)	29.67 (21.22)	27.58 (21.9)	6.41	4.87	4.71	3.54	4.67
**C4**	2238	2315	685	110	69	0.35	0.36	0.34	0.38	0.47	42.23 (16.53)	37.02 (20.06)	36.32 (21.14)	29.97 (21.35)	27.31 (21.86)	6.44	4.92	4.76	3.58	4.75
**FFA**	4761	4561	1048	213	83	0.65	0.73	0.74	0.75	0.93	49.76 (17.17)	47.6 (21.11)	47.74 (21.26)	47.15 (22.6)	46.85 (22.12)	4.11	3.87	2.63	1.8	1.77
**GA**	961	763	170	36	8	0.79	0.99	1.02	1.12	3	48.67 (15.81)	49.5 (17.85)	49.35 (17.57)	50.36 (18.78)	33.75 (18.24)	11.16	15.96	7.1	8	7
**Cr(E)**	18032	17894	4432	759	340	0.62	0.67	0.63	0.65	0.69	48.33 (17.83)	45.18 (21.56)	45.28 (21.81)	43.47 (22.85)	40.7 (23.71)	7.76	5.59	3.91	3.17	3
**SI**	1182	1086	317	45	22	0.46	0.67	0.59	0.73	0.57	43.98 (18.38)	41.36 (22.08)	42.3 (22.87)	34.76 (24.46)	35.93 (24.8)	3.51	2.77	2.23	2.21	4.5
**TRF**	1183	1082	323	55	27	0.46	0.67	0.6	0.72	0.59	44 (18.37)	41.29 (22.04)	41.53 (23.36)	28.51 (25.81)	31.59 (26.46)	3.52	2.77	2.14	1.39	2.38
**SF**	1575	1470	433	73	38	0.46	0.62	0.55	0.62	0.52	43.44 (17.76)	41.07 (21.44)	40.41 (22.29)	32.56 (25.56)	34.49 (24.28)	3.16	2.35	1.87	1.52	1.24
**Sfa**	936	924	241	25	15	0.61	0.71	0.54	1.08	0.36	44.87 (18.24)	44.22 (20.98)	46.11 (21.37)	39.08 (25.43)	44.71 (18.96)	3.75	3	2.26	1.08	4
**VB12**	1096	1075	289	40	27	0.58	0.7	0.57	0.9	0.42	45.58 (18.32)	43.73 (21.78)	44.18 (22.26)	36.38 (25.78)	37.51 (22.83)	3.79	3.07	2.11	1.5	2

ALT, Alanine Aminotransferase; TP, Total Protein; Alb, Albumin; TBil, Total Bilirubin; DBil, Direct Bilirubin; GGT, Gamma-Glutamyl Transferase; ALP, Alkaline Phosphatase; AST, Aspartate Aminotransferase; TBA, Total Bile Acid; CK, Creatine Kinase; LD, Lactate Dehydrogenase; ChE, Cholinesterase; K, Potassium; Na, Sodium; Cl, Chloride; TCO2, Total Carbon Dioxide; Ca, Calcium; Urea, Urea Nitrogen; Glu, Glucose; UA, Uric Acid; P, Phosphorus; TC, Total Cholesterol; TG, Triglycerides; HDL_C, High-Density Lipoprotein Cholesterol; LDL_C, Low-Density Lipoprotein Cholesterol; ApoA1, Apolipoprotein A1; ApoB, Apolipoprotein B; Lp(a), Lipoprotein(a); hsCRP, High Sensitivity C-Reactive Protein; Mg, Magnesium; PA, Prealbumin; RF, Rheumatoid Factor; HCY, Homocysteine; IgG, Immunoglobulin G; IgA, Immunoglobulin A; IgM, Immunoglobulin M; ASO, Antistreptolysin O; CysC, Cystatin C; C3, Complement Component 3; C4, Complement Component 4; FFA, Free Fatty Acids; GA, Glycated Albumin; Cr(E), Creatinine; SI, Serum Iron; TRF, Transferrin; SF, Serum Ferritin; Sfa, Serum Folic Acid; VB12, Vitamin B12.

Note: Values in parentheses represent the Standard Deviation for normally distributed variables.

### 3.2. Univariate analysis of hemolysis on biochemical and immunological analytes

**Fig 2:**
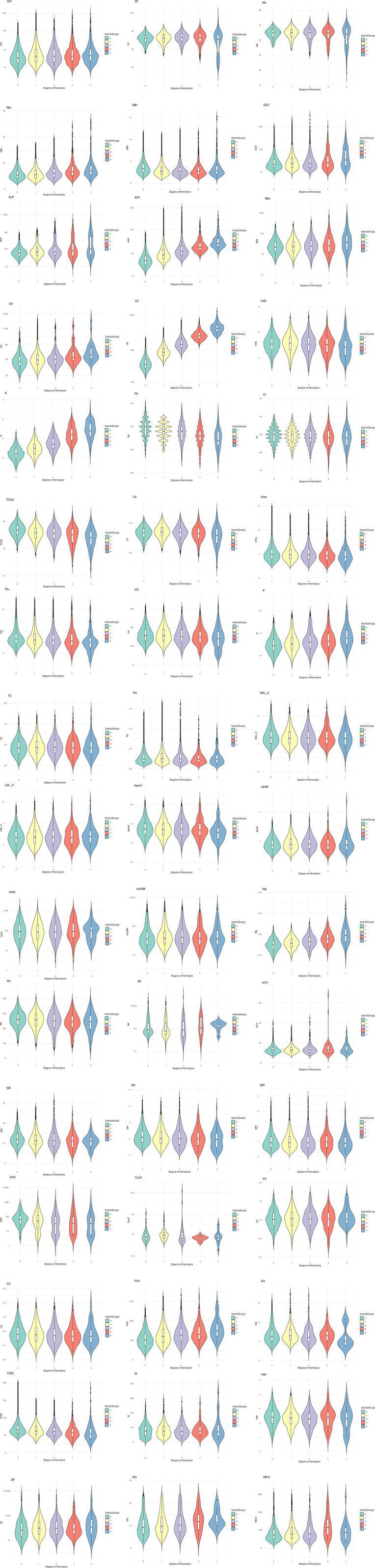
Distribution and levels of biochemical and immunological analytes across different hemolysis groups.

In our analysis, while TC, Lp(a), IgM, C3, and SI showed no statistically significant differences across different degrees of hemolysis, the impacts on other analytes were significant (Table 2). Specifically, AST, CK, LD, K, P, Mg, and FFA levels increased with the severity of hemolysis. In contrast, Cr and TCO2 levels decreased as hemolysis intensity escalated (Fig 2). These findings illustrate the variable impact of hemolysis on biochemical and immunological analytes, highlighting the importance of considering hemolysis degree in clinical evaluations. Detailed results of the post-hoc analysis are provided in S2 File.

**Table 2:** Univariate analysis of the impact of hemolysis on biochemical and immunological biomarkers.

Makers	H-index_0	H-index_1	H-index_2	H-index_3	H-index_4	P_Value
**ALT**	18.00 (14.00)	20.00 (17.00)	20.00 (17.00)	20.00 (16.08)	21.00 (16.00)	<0.001
**TP**	72.00 (7.00)	72.00 (8.00)	73.00 (8.00)	73.00 (10.00)	71.00 (20.00)	<0.001
**Alb**	44.00 (4.00)	45.00 (5.00)	44.00 (6.00)	45.00 (6.20)	44.00 (11.00)	<0.001
**TBil**	10.70 (5.90)	11.00 (6.10)	11.90 (6.50)	13.30 (7.20)	14.20 (7.82)	<0.001
**DBil**	3.30 (2.00)	2.70 (1.80)	2.60 (1.80)	2.70 (1.80)	2.80 (2.00)	<0.001
**GGT**	22.00 (21.00)	24.00 (25.00)	24.00 (27.00)	26.00 (35.00)	34.00 (74.00)	<0.001
**ALP**	76.00 (36.00)	82.00 (47.00)	82.00 (52.00)	87.00 (82.00)	100.00 (161.50)	<0.001
**AST**	21.00 (10.00)	28.00 (14.00)	34.00 (16.00)	42.00 (16.25)	50.00 (21.00)	<0.001
**TBA**	2.20 (2.60)	2.30 (2.90)	2.40 (3.20)	2.50 (4.20)	3.70 (6.60)	<0.001
**CK**	87.00 (71.00)	98.00 (87.75)	100.00 (77.50)	116.50 (97.00)	141.50 (123.00)	<0.001
**LD**	184.00 (56.00)	279.00 (80.00)	367.00 (105.67)	490.50 (106.25)	637.00 (142.25)	<0.001
**ChE**	7.70 (2.40)	7.90 (2.60)	7.70 (2.70)	7.60 (2.70)	7.00 (2.90)	<0.001
**K**	4.20 (0.50)	4.50 (0.50)	4.80 (0.50)	5.20 (0.60)	5.40 (0.70)	<0.001
**Na**	140.00 (3.00)	139.00 (2.00)	139.00 (3.00)	138.00 (3.00)	137.00 (3.00)	<0.001
**Cl**	105.00 (3.00)	104.00 (3.00)	104.00 (3.00)	104.00 (4.00)	104.00 (4.00)	<0.001
**TCO2**	26.90 (3.50)	25.80 (3.80)	25.60 (4.00)	25.10 (5.00)	24.00 (5.00)	<0.001
**Ca**	2.33 (0.14)	2.35 (0.15)	2.33 (0.16)	2.32 (0.18)	2.29 (0.25)	<0.001
**Urea**	4.91 (2.10)	4.88 (2.08)	4.87 (2.18)	4.64 (2.04)	4.40 (2.32)	<0.001
**Glu**	5.30 (1.10)	5.20 (1.10)	5.20 (1.10)	5.10 (1.20)	5.00 (1.20)	<0.001
**UA**	313.00 (124.00)	312.00 (128.00)	302.00 (130.00)	292.00 (125.00)	280.00 (159.50)	<0.001
**P**	1.22 (0.26)	1.26 (0.29)	1.30 (0.29)	1.33 (0.30)	1.39 (0.33)	<0.001
**TC**	4.62 (1.43)	4.65 (1.47)	4.68 (1.50)	4.60 (1.54)	4.57 (1.55)	0.081
**TG**	1.22 (0.98)	1.27 (1.09)	1.24 (1.06)	1.20 (0.92)	1.17 (1.00)	0.004
**HDL_C**	1.21 (0.45)	1.19 (0.44)	1.20 (0.45)	1.18 (0.46)	1.18 (0.49)	<0.001
**LDL_C**	2.68 (1.16)	2.72 (1.24)	2.72 (1.24)	2.68 (1.27)	2.78 (1.18)	0.022
**ApoA1**	1.36 (0.31)	1.35 (0.31)	1.36 (0.32)	1.33 (0.32)	1.28 (0.32)	0.004
**ApoB**	0.87 (0.32)	0.89 (0.35)	0.90 (0.34)	0.88 (0.36)	0.87 (0.37)	0.004
**Lp(a)**	89.00 (166.00)	84.00 (158.00)	87.00 (165.25)	89.00 (187.50)	101.00 (116.00)	0.386
**hsCRP**	1.29 (3.59)	1.47 (4.00)	1.66 (4.87)	1.41 (3.58)	1.32 (4.38)	<0.001
**Mg**	0.87 (0.10)	0.89 (0.10)	0.91 (0.10)	0.94 (0.11)	0.99 (0.17)	<0.001
**PA**	260.00 (75.00)	258.00 (81.00)	250.00 (89.00)	242.00 (78.00)	244.00 (92.50)	<0.001
**RF**	7.00 (27.98)	6.00 (26.20)	4.00 (30.20)	2.63 (19.00)	1.20 (27.85)	<0.001
**HCY**	12.60 (5.00)	13.00 (5.00)	13.20 (5.25)	13.10 (6.68)	12.30 (6.25)	0.057
**IgG**	11.93 (5.21)	11.32 (5.38)	11.12 (6.12)	11.12 (5.88)	10.91 (4.46)	<0.001
**IgA**	2.17 (1.47)	2.04 (1.49)	2.01 (1.58)	1.97 (1.64)	1.92 (1.77)	<0.001
**IgM**	0.91 (0.70)	0.92 (0.74)	0.91 (0.76)	0.98 (0.80)	0.85 (0.80)	0.320
**ASO**	64.25 (95.17)	49.05 (107.43)	32.00 (93.00)	35.00 (91.00)	34.00 (83.50)	<0.001
**CysC**	0.88 (0.38)	0.96 (0.33)	0.79 (0.44)	0.84 (0.11)	0.84 (0.31)	0.015
**C3**	1.01 (0.34)	1.03 (0.36)	1.02 (0.34)	1.02 (0.38)	1.02 (0.32)	0.535
**C4**	0.18 (0.09)	0.17 (0.10)	0.17 (0.10)	0.17 (0.09)	0.16 (0.09)	0.004
**FFA**	508.00 (298.00)	593.00 (335.00)	627.00 (334.25)	654.00 (319.00)	745.00 (333.00)	<0.001
**GA**	6.27 (2.87)	6.44 (2.81)	5.94 (3.08)	6.48 (2.42)	5.11 (2.55)	0.05
**Cr(E)**	66.00 (24.00)	62.00 (25.00)	59.00 (24.00)	56.00 (25.50)	53.50 (30.25)	<0.001
**SI**	77.00 (65.90)	75.70 (62.30)	74.00 (64.00)	74.00 (56.00)	70.00 (78.49)	0.855
**TRF**	2.39 (0.88)	2.34 (0.82)	2.29 (0.81)	2.41 (0.96)	2.30 (1.02)	0.038
**SF**	64.00 (235.00)	95.00 (304.75)	92.00 (305.00)	78.00 (198.00)	114.00 (447.75)	<0.001
**Sfa**	9.15 (6.60)	9.70 (6.70)	10.20 (6.80)	11.80 (6.70)	11.30 (4.30)	<0.001
**VB12**	356.00 (265.75)	384.00 (303.00)	386.00 (348.00)	569.50 (427.00)	428.00 (330.50)	<0.001

ALT, Alanine Aminotransferase; TP, Total Protein; Alb, Albumin; TBil, Total Bilirubin; DBil, Direct Bilirubin; GGT, Gamma-Glutamyl Transferase; ALP, Alkaline Phosphatase; AST, Aspartate Aminotransferase; TBA, Total Bile Acid; CK, Creatine Kinase; LD, Lactate Dehydrogenase; ChE, Cholinesterase; K, Potassium; Na, Sodium; Cl, Chloride; TCO2, Total Carbon Dioxide; Ca, Calcium; Urea, Urea Nitrogen; Glu, Glucose; UA, Uric Acid; P, Phosphorus; TC, Total Cholesterol; TG, Triglycerides; HDL_C, High-Density Lipoprotein Cholesterol; LDL_C, Low-Density Lipoprotein Cholesterol; ApoA1, Apolipoprotein A1; ApoB, Apolipoprotein B; Lp(a), Lipoprotein(a); hsCRP, High Sensitivity C-Reactive Protein; Mg, Magnesium; PA, Prealbumin; RF, Rheumatoid Factor; HCY, Homocysteine; IgG, Immunoglobulin G; IgA, Immunoglobulin A; IgM, Immunoglobulin M; ASO, Antistreptolysin O; CysC, Cystatin C; C3, Complement Component 3; C4, Complement Component 4; FFA, Free Fatty Acids; GA, Glycated Albumin; Cr(E), Creatinine; SI, Serum Iron; TRF, Transferrin; SF, Serum Ferritin; Sfa, Serum Folic Acid; VB12, Vitamin B12.

Note: Values in parentheses represent the Interquartile Range for non-normally distributed variables.

### 3.3. Sensitivity analysis excluding participants under 18 years of age

In our sensitivity analysis, which excluded participants under the age of 18, the impact of hemolysis on biochemical and immunological analytes was reassessed using univariate analysis. This approach revealed that the effects of hemolysis on IgG, IgA, CysC, C4, and GA became statistically non-significant after removing data from underage participants. Conversely, the influence of hemolysis on TC shifted to become significant. The outcomes for other analytes remained unchanged **(S1 Table in S1 File)**. These results underscore the importance of considering age as a potential confounder in the evaluation of hemolysis effects on specific analytes.

### 3.4. Multivariate analysis of hemolysis impact on biochemical and immunological analytes

**Fig 3:**
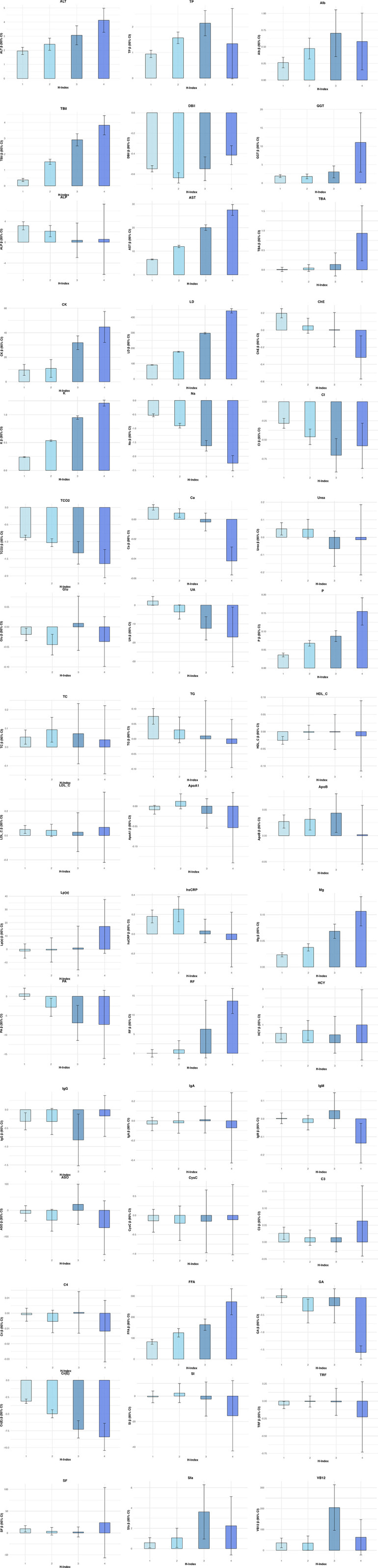
Visualization of the impact of hemolysis on biochemical and immunological analytes after adjusting for confounding factors in the multivariate analysis.

After adjusting for gender, age, patient type, and PVD, with a hemolysis index of 0 as the reference point, hemolysis significantly positively influenced ALT, Alb, TBil, GGT, AST, CK, LD, K, P, Mg, and FFA. Conversely, the impact on DBil, Na, Cl, TCO2, and Cr was significantly negative. High levels of hemolysis negatively interfere with UA, PA, and GA. The effects on other analytes did not exhibit a consistent pattern of significance (Table 3). Within the analytes significantly affected, the positive impact of hemolysis on ALT, TBil, AST, CK, LD, K, P, Mg, and FFA intensifies with an increase in the hemolysis index. Simultaneously, the negative effects on Na TCO2, and Cr also amplify as the hemolysis index rises (Fig 3). Compared to the univariate analysis, the multivariate analysis, which adjusted for potential confounding factors, demonstrated that the effects of hemolysis on ALT, TBil, GGT, and Na were of trend-level statistical significance(P < 0.05). The negative interference of hemolysis on RF was not observed in the multivariate analysis. The hemolysis impact on analytes underwent modifications after adjusting for confounding factors in the multivariate analysis, which also provided 95% CI estimates for the effect sizes of hemolysis. This adjustment offers a more precise understanding of how hemolysis influences each analyte, underlining the importance of considering various patient and clinical characteristics when interpreting the effects of hemolysis on laboratory results. Detailed model diagnostic metrics (including Pseudo-R^2, AIC, and RMSE) are provided in **S3 File**, and the corresponding residual diagnostic plots are available in **S4 File**.

**Table 3:** Multivariate analysis of hemolysis impact on biochemical and immunological analytes adjusted for gender, age, patient type, PDV.

Markers	H-index_1 (β(95%CI, P value))	H-index_2 (β(95%CI, P value))	H-index_3 (β(95%CI, P value))	H-index_4 (β(95%CI, P value))	P for Trend
ALT	1.95 (1.69–2.21, p = 0.000)	2.43 (2.00–2.86, p = 0.000)	3.06 (2.38–3.74, p = 0.000)	4.13 (3.27–4.98, p = 0.000)	<0.05
TP	0.94 (0.80–1.08, p = 0.000)	1.57 (1.35–1.80, p = 0.000)	2.14 (1.65–2.63, p = 0.000)	1.34 (−0.03–2.71, p = 0.055)	0.75
Alb	0.26 (0.18–0.34, p = 0.000)	0.47 (0.32–0.63, p = 0.000)	0.70 (0.35–1.05, p = 0.000)	0.58 (0.15–1.00, p = 0.008)	0.167
TBil	0.37 (0.26–0.48, p = 0.000)	1.52 (1.35–1.68, p = 0.000)	2.91 (2.52–3.29, p = 0.000)	3.83 (3.22–4.44, p = 0.000)	<0.05
DBil	−0.55 (−0.58–0.52, p = 0.000)	−0.64 (−0.69–0.59, p = 0.000)	−0.55 (−0.66–0.43, p = 0.000)	−0.41 (−0.51–0.32, p = 0.000)	0.375
GGT	1.91 (1.51–2.30, p = 0.000)	1.79 (1.14–2.44, p = 0.000)	3.02 (1.40–4.64, p = 0.000)	11.02 (2.96–19.08, p = 0.007)	0.167
ALP	3.15 (2.37–3.93, p = 0.000)	2.12 (1.04–3.20, p = 0.000)	0.37 (−2.94–3.68, p = 0.828)	0.56 (−6.17–7.29, p = 0.870)	0.5
AST	6.50 (6.30–6.70, p = 0.000)	12.00 (11.50–12.50, p = 0.000)	20.00 (18.82–21.18, p = 0.000)	27.50 (25.18–29.82, p = 0.000)	<0.05
TBA	0.01 (−0.04–0.06, p = 0.723)	0.05 (−0.04–0.13, p = 0.310)	0.14 (−0.16–0.43, p = 0.368)	0.93 (0.23–1.64, p = 0.010)	0.167
CK	9.62 (5.13–14.10, p = 0.000)	10.82 (3.53–18.10, p = 0.004)	31.85 (26.26–37.43, p = 0.000)	44.69 (32.03–57.35, p = 0.000)	<0.05
LD	90.91 (89.57–92.24, p = 0.000)	176.36 (173.19–179.54, p = 0.000)	297.09 (292.17–302.01, p = 0.000)	441.91 (427.71–456.11, p = 0.000)	<0.05
ChE	0.20 (0.14–0.25, p = 0.000)	0.05 (−0.04–0.14, p = 0.259)	0.00 (−0.20–0.21, p = 0.967)	−0.32 (−0.57–0.07, p = 0.013)	0.125
K	0.24 (0.23–0.25, p = 0.000)	0.53 (0.52–0.55, p = 0.000)	0.95 (0.92–0.98, p = 0.000)	1.21 (1.16–1.26, p = 0.000)	<0.05
Na	−0.53 (−0.58–0.48, p = 0.000)	−0.90 (−0.99–0.82, p = 0.000)	−1.62 (−1.81–1.44, p = 0.000)	−2.25 (−2.52–1.97, p = 0.000)	<0.05
Cl	−0.28 (−0.35–0.22, p = 0.000)	−0.46 (−0.56–0.36, p = 0.000)	−0.70 (−0.92–0.48, p = 0.000)	−0.58 (−0.88–0.28, p = 0.000)	0.167
TCO2	−0.88 (−0.95–0.81, p = 0.000)	−1.03 (−1.14–0.92, p = 0.000)	−1.33 (−1.66–1.00, p = 0.000)	−1.64 (−2.05–1.23, p = 0.000)	<0.05
Ca	0.01 (0.01–0.01, p = 0.000)	0.01 (0.00–0.01, p = 0.004)	−0.00 (−0.01–0.01, p = 0.552)	−0.04 (−0.06–0.03, p = 0.000)	0.083
Urea	0.05 (0.01–0.08, p = 0.008)	0.05 (−0.01–0.10, p = 0.103)	−0.07 (−0.17–0.04, p = 0.204)	−0.01 (−0.22–0.19, p = 0.886)	0.667
Glu	−0.02 (−0.03–0.00, p = 0.017)	−0.04 (−0.07–0.02, p = 0.001)	0.01 (−0.06–0.08, p = 0.797)	−0.04 (−0.10–0.03, p = 0.249)	1
UA	2.26 (−0.07–4.59, p = 0.057)	−3.63 (−7.43–0.17, p = 0.061)	−12.37 (−18.57–6.17, p = 0.000)	−17.00 (−32.93–1.07, p = 0.036)	0.125
P	0.04 (0.03–0.04, p = 0.000)	0.07 (0.06–0.08, p = 0.000)	0.09 (0.07–0.10, p = 0.000)	0.15 (0.12–0.19, p = 0.000)	<0.05
TC	0.05 (0.02–0.09, p = 0.006)	0.09 (0.03–0.16, p = 0.006)	0.07 (−0.09–0.23, p = 0.384)	0.04 (−0.14–0.22, p = 0.673)	1
TG	0.07 (0.05–0.10, p = 0.000)	0.03 (−0.01–0.07, p = 0.170)	0.01 (−0.11–0.13, p = 0.867)	−0.02 (−0.10–0.06, p = 0.705)	0.167
HDL_C	−0.03 (−0.04–0.01, p = 0.000)	−0.00 (−0.02–0.02, p = 0.844)	−0.00 (−0.05–0.05, p = 0.968)	−0.01 (−0.11–0.09, p = 0.812)	1
LDL_C	0.05 (0.02–0.08, p = 0.003)	0.04 (−0.01–0.09, p = 0.099)	0.03 (−0.13–0.19, p = 0.737)	0.07 (−0.22–0.36, p = 0.649)	1
ApoA1	−0.01 (−0.02–0.00, p = 0.112)	0.01 (−0.01–0.03, p = 0.208)	−0.02 (−0.05–0.02, p = 0.334)	−0.05 (−0.14–0.03, p = 0.230)	0.833
ApoB	0.03 (0.01–0.04, p = 0.000)	0.03 (0.01–0.05, p = 0.003)	0.04 (0.01–0.08, p = 0.022)	0.00 (−0.05–0.06, p = 0.947)	0.917
Lp(a)	−1.39 (−6.80–4.02, p = 0.614)	−0.60 (−9.78–8.59, p = 0.899)	0.93 (−15.54–17.40, p = 0.912)	17.18 (−3.16–37.53, p = 0.098)	0.208
hsCRP	0.18 (0.11–0.25, p = 0.000)	0.25 (0.13–0.38, p = 0.000)	0.03 (−0.09–0.15, p = 0.608)	−0.06 (−0.34–0.22, p = 0.689)	0.5
Mg	0.02 (0.02–0.03, p = 0.000)	0.04 (0.03–0.04, p = 0.000)	0.07 (0.05–0.08, p = 0.000)	0.11 (0.08–0.13, p = 0.000)	<0.05
PA	0.66 (−0.81–2.12, p = 0.380)	−2.84 (−5.19–0.49, p = 0.018)	−6.90 (−11.46–2.34, p = 0.003)	−7.28 (−16.12–1.56, p = 0.107)	0.125
RF	−0.00 (−0.97–0.97, p = 1.000)	0.93 (−1.43–3.30, p = 0.439)	6.29 (−1.20–13.78, p = 0.100)	13.60 (10.35–16.85, p = 0.000)	0.167
HCY	0.52 (0.20–0.85, p = 0.002)	0.69 (0.14–1.24, p = 0.014)	0.44 (−0.59–1.47, p = 0.398)	1.00 (−0.96–2.96, p = 0.319)	1
IgG	−0.32 (−0.55–0.08, p = 0.008)	−0.32 (−0.68–0.04, p = 0.077)	−0.82 (−1.52–0.12, p = 0.022)	−0.17 (−0.73–0.38, p = 0.548)	1
IgA	−0.03 (−0.10–0.04, p = 0.347)	−0.02 (−0.13–0.09, p = 0.702)	0.01 (−0.12–0.15, p = 0.862)	−0.07 (−0.43–0.29, p = 0.695)	1
IgM	0.00 (−0.03–0.03, p = 0.878)	−0.02 (−0.06–0.02, p = 0.296)	0.05 (−0.05–0.14, p = 0.369)	−0.14 (−0.25–0.03, p = 0.016)	1
ASO	−11.56 (−40.52–17.40, p = 0.434)	−37.68 (−79.42–4.07, p = 0.077)	22.73 (−53.72–99.17, p = 0.560)	−66.16 (−168.45–36.12, p = 0.205)	1
CysC	−0.14 (−0.44–0.15, p = 0.348)	−0.21 (−0.66–0.24, p = 0.368)	−0.16 (−0.97–0.66, p = 0.709)	−0.12 (−1.03–0.80, p = 0.805)	1
C3	0.03 (0.01–0.04, p = 0.005)	0.01 (−0.01–0.04, p = 0.263)	0.01 (−0.03–0.06, p = 0.541)	0.06 (−0.04–0.17, p = 0.240)	1
C4	−0.00 (−0.01–0.00, p = 0.658)	−0.01 (−0.01–0.00, p = 0.152)	0.00 (−0.01–0.01, p = 0.938)	−0.01 (−0.03–0.01, p = 0.253)	1
FFA	82.28 (70.57–93.99, p = 0.000)	125.07 (104.88–145.26, p = 0.000)	163.69 (136.69–190.69, p = 0.000)	272.66 (210.83–334.49, p = 0.000)	<0.05
GA	0.05 (−0.15–0.25, p = 0.606)	−0.39 (−0.73–0.04, p = 0.027)	−0.24 (−0.73–0.26, p = 0.344)	−1.59 (−1.78–1.39, p = 0.000)	0.5
Cr(E)	−3.11 (−3.44–2.78, p = 0.000)	−4.97 (−5.58–4.37, p = 0.000)	−7.29 (−8.58–6.00, p = 0.000)	−8.41 (−10.43–6.39, p = 0.000)	<0.05
SI	−0.66 (−5.26–3.95, p = 0.780)	2.33 (−5.19–9.85, p = 0.543)	−2.40 (−15.81–11.01, p = 0.726)	−15.49 (−43.09–12.12, p = 0.272)	0.833
TRF	−0.06 (−0.11–0.01, p = 0.029)	−0.01 (−0.09–0.07, p = 0.872)	−0.01 (−0.21–0.18, p = 0.905)	−0.23 (−0.73–0.28, p = 0.376)	1
SF	9.55 (2.48–16.62, p = 0.008)	3.76 (−4.96–12.48, p = 0.398)	2.30 (−9.12–13.71, p = 0.693)	23.45 (−57.09–104.00, p = 0.568)	1
Sfa	0.58 (0.07–1.08, p = 0.025)	1.06 (0.11–2.01, p = 0.029)	3.62 (0.94–6.29, p = 0.008)	2.24 (−0.64–5.12, p = 0.127)	0.333
VB12	35.42 (12.45–58.39, p = 0.003)	34.55 (0.48–68.62, p = 0.047)	204.47 (95.29–313.65, p = 0.000)	62.33 (−22.71–147.37, p = 0.151)	0.417

PDV, Patient’s Department of Visit; ALT, Alanine Aminotransferase; TP, Total Protein; Alb, Albumin; TBil, Total Bilirubin; DBil, Direct Bilirubin; GGT, Gamma-Glutamyl Transferase; ALP, Alkaline Phosphatase; AST, Aspartate Aminotransferase; TBA, Total Bile Acid; CK, Creatine Kinase; LD, Lactate Dehydrogenase; ChE, Cholinesterase; K, Potassium; Na, Sodium; Cl, Chloride; TCO2, Total Carbon Dioxide; Ca, Calcium; Urea, Urea Nitrogen; Glu, Glucose; UA, Uric Acid; P, Phosphorus; TC, Total Cholesterol; TG, Triglycerides; HDL_C, High-Density Lipoprotein Cholesterol; LDL_C, Low-Density Lipoprotein Cholesterol; ApoA1, Apolipoprotein A1; ApoB, Apolipoprotein B; Lp(a), Lipoprotein(a); hsCRP, High Sensitivity C-Reactive Protein; Mg, Magnesium; PA, Prealbumin; RF, Rheumatoid Factor; HCY, Homocysteine; IgG, Immunoglobulin G; IgA, Immunoglobulin A; IgM, Immunoglobulin M; ASO, Antistreptolysin O; CysC, Cystatin C; C3, Complement Component 3; C4, Complement Component 4; FFA, Free Fatty Acids; GA, Glycated Albumin; Cr(E), Creatinine; SI, Serum Iron; TRF, Transferrin; SF, Serum Ferritin; Sfa, Serum Folic Acid; VB12, Vitamin B12.

### 3.5. Comparison with experimentally obtained results

The results in our study derived from big data analysis were compared with those [14] obtained through experimental methods on the same platform and hemolysis index assessment techniques **(Table 4)**. The comparison revealed that 38 parameters, including K, Na, and LD, yielded consistent results across both studies. Notably, for key analytes such as K, the degree of hemolysis impact calculated through big data closely matched the experimental findings. However, discrepancies were observed in the results for GGT, ALP, and RF.

**Table 4:** Comparison with results from other studies.

Index	This study	Xia Liangyu’study	Are the trends consistent
**Research Methods**	Real-world big data	Experiment	/
**Platform**	Beckman Coulter	Beckman Coulter	/
**ALT**	Positive interference	Positive interference	Yes
**TP**	No obvious effect	No obvious effect	Yes
**Alb**	Positive interference	Positive interference	Yes
**TBil**	Positive interference	Positive interference	Yes
**DBil**	Negative interference	Negative interference	Yes
**GGT**	Positive interference	No obvious effect	No
**ALP**	No obvious effect	Negative interference	No
**AST**	Positive interference	Positive interference	Yes
**TBA**	No obvious effect	No obvious effect	Yes
**CK**	Positive interference	Positive interference	Yes
**LD**	Positive interference	Positive interference	Yes
**ChE**	No obvious effect	No obvious effect	Yes
**K**	Positive interference	Positive interference	Yes
**Na**	Negative interference	Negative interference	Yes
**Cl**	Negative interference	Negative interference	Yes
**TCO2**	Negative interference	Not reported	Not available
**Ca**	No obvious effect	No obvious effect	Yes
**Urea**	No obvious effect	No obvious effect	Yes
**Glu**	No obvious effect	No obvious effect	Yes
**UA**	Negative interference*	Negative interference	Yes
**P**	Positive interference	Positive interference	Yes
**TC**	No obvious effect	No obvious effect	Yes
**TG**	No obvious effect	No obvious effect	Yes
**HDL_C**	No obvious effect	No obvious effect	Yes
**LDL_C**	No obvious effect	No obvious effect	Yes
**ApoA1**	No obvious effect	No obvious effect	Yes
**ApoB**	No obvious effect	No obvious effect	Yes
**Lp(a)**	No obvious effect	No obvious effect	Yes
**hsCRP**	No obvious effect	No obvious effect	Yes
**Mg**	Positive interference	Not reported	Not available
PA	Negative interference*	Negative interference	Yes
**RF**	No obvious effect	Negative interference	No
**HCY**	No obvious effect	No obvious effect	Yes
**IgG**	No obvious effect	No obvious effect	Yes
**IgA**	No obvious effect	No obvious effect	Yes
**IgM**	No obvious effect	Negative interference	No
**ASO**	No obvious effect	No obvious effect	Yes
**CysC**	No obvious effect	No obvious effect	Yes
**C3**	No obvious effect	No obvious effect	Yes
**C4**	No obvious effect	No obvious effect	Yes
**FFA**	Positive interference	Not reported	Not available
**GA**	Negative interference*	Not reported	Not available
**Cr(E)**	Negative interference	Negative interference	Yes
**SI**	No obvious effect	No obvious effect	Yes
**TRF**	No obvious effect	No obvious effect	Yes
**SF**	No obvious effect	Not reported	Not available
**Sfa**	Positive interference*	Not reported	Not available
**VB12**	Positive interference*	Not reported	Not available

ALT, Alanine Aminotransferase; TP, Total Protein; Alb, Albumin; TBil, Total Bilirubin; DBil, Direct Bilirubin; GGT, Gamma-Glutamyl Transferase; ALP, Alkaline Phosphatase; AST, Aspartate Aminotransferase; TBA, Total Bile Acid; CK, Creatine Kinase; LD, Lactate Dehydrogenase; ChE, Cholinesterase; K, Potassium; Na, Sodium; Cl, Chloride; TCO2, Total Carbon Dioxide; Ca, Calcium; Urea, Urea Nitrogen; Glu, Glucose; UA, Uric Acid; P, Phosphorus; TC, Total Cholesterol; TG, Triglycerides; HDL_C, High-Density Lipoprotein Cholesterol; LDL_C, Low-Density Lipoprotein Cholesterol; ApoA1, Apolipoprotein A1; ApoB, Apolipoprotein B; Lp(a), Lipoprotein(a); hsCRP, High Sensitivity C-Reactive Protein; Mg, Magnesium; PA, Prealbumin; RF, Rheumatoid Factor; HCY, Homocysteine; IgG, Immunoglobulin G; IgA, Immunoglobulin A; IgM, Immunoglobulin M; ASO, Antistreptolysin O; CysC, Cystatin C; C3, Complement Component 3; C4, Complement Component 4; FFA, Free Fatty Acids; GA, Glycated Albumin; Cr(E), Creatinine; SI, Serum Iron; TRF, Transferrin; SF, Serum Ferritin; Sfa, Serum Folic Acid; VB12, Vitamin B12; No obvious effectthe impact of hemolysis on the test parameter does not show significant changes or variations as the degree of hemolysis varies;Positive interference*, when the hemolysis index is at 4, the small sample size may lead to unstable results, making it difficult to draw reliable conclusions at this level of hemolysis. However, an observable trend is evident across hemolysis indices from 1 to 3, suggesting a consistent pattern or effect of hemolysis on the test parameter within these ranges;Negative interference*,High levels of hemolysis negatively interfere with UA, PA, and GA.

## 4. Discussion

In this study, we leveraged real-world clinical laboratory data to analyze the impact of in vitro hemolysis on 48 biochemical and immunological analytes using non-parametric regression and other methods. Extensive work was undertaken in data cleaning and the presentation of baseline information, highlighting data cleaning as a critical step in big data analysis [15]. The use of regular expressions for handling text variables ensured the correct extraction of hemolysis indices, while secondary database searches were employed to address missing variable information and verify data integrity. Importantly, the study excluded results for samples with a hemolysis index of 5 due to their minimal volume, which could significantly impact model construction and parameter estimation. Future researchers are advised to exercise caution when dealing with groups of small sample sizes to prevent bias in parameter estimates. Additionally, during the data cleaning process, our study established stringent criteria for data inclusion and exclusion, eliminating diseases associated with in vivo hemolysis and excluding observations that could potentially affect experimental outcomes, such as cold agglutinin disease, from the study. The aim was to minimize interference with the research findings, ensuring that the analysis accurately reflects the impact of in vitro hemolysis on laboratory analytes without confounding from unrelated pathological conditions.

In presenting baseline information, given the potential for false positives due to large sample sizes [16], only descriptive statistical results were objectively presented. These results highlighted differences in gender composition, age, and patient types across different hemolysis index groups. Such confounding factors’ imbalance across groups poses a potential risk to the study’s outcomes, underscoring the necessity of adjustment in modeling efforts.

The baseline information table highlights imbalances across different hemolysis groups, underscoring the necessity of conducting multivariate analysis. Given the small sample size for certain analytes with a H-index of 4, and to observe changes introduced by multivariate adjustments, we performed univariate analysis prior to multivariate modeling. This approach allowed us to objectively present the levels and distributions of various test parameters across different H-index groups before adjustment, providing a clear foundation for subsequent analyses.

Acknowledging the influence of age on various analytes, our study conducted an additional round of univariate analysis after excluding minors. This adjustment led to a shift in the conclusions for indicators such as IgG, IgA, CysC, C4, and GA. These steps is crucial not only for identifying potential confounding factors but also for ensuring the robustness and validity of the multivariate models developed later in the study.

In our study, we utilized clinical laboratory real-world data to analyze the impact of in vitro hemolysis on 48 biochemical and immunological analytes. Our findings corroborate with those of Ji JZ et al. [17], who noted severe negative interference of hemolysis on PA, consistent with our observations. Additionally, our research identified a negative interference of hemolysis on Cr, diverging from the results presented by Mehmet Koseoglu [6], likely due to differing creatinine detection methodologies. The effect of hemolysis on TBil is a subject of debate [6,17,18]; our results indicate a positive interference, adding to the discourse. For analytes like ALT, GGT, AST, CK, LD, K, P, Mg, and FFA, a significant positive interference from hemolysis was observed, aligning with the conclusions of many previous studies [19,20]. Among them, the effect of analytes such as ALT was statistically significant, but the clinical significance was small. The discrepancy in findings emphasizes the need for clinical laboratories to assess the impact of hemolysis based on their specific testing platforms and devise suitable strategies for managing hemolyzed samples. Further, through multivariate modeling adjusted for confounders, minor changes were noted in the impact magnitude of hemolysis on analytes like K, without altering the trend. For analytes significantly affected by confounders, such as TCO2, adjustments revealed a trend-level impact. Comparing our multivariate analysis results with experimental data showed a high agreement rate of 93.0%, except for discrepancies in GGT, ALP, and RF. The discrepancy in RF could be attributed to our study’s small sample size compared to other analytes, potentially leading to biased estimates. The inconsistency in GGT results may relate to Xia’s study’s [14] limited sample size, impacting the statistical power, while the discrepancy in ALP may be due to the extreme rightward skewness of ALP that leads to bias in estimating the trend in small samples.

The high consistency between our big data analysis and experimental findings validates the rationale for multivariate adjustments. Importantly, it confirms the feasibility of conducting correlative analyses using big data. This convergence underscores the robustness of big data methodologies in replicating traditional experimental outcomes, offering a compelling case for their integration into clinical laboratory research and decision-making processes.

Crucially, our analysis of β values allows for a nuanced differentiation between statistical significance and clinical relevance, guiding precise laboratory interventions. While analytes such as ALT, TBil, P, TCO2, and Na exhibited statistically significant trends (P < 0.05) across hemolysis groups, the magnitude of these shifts (small β values) is likely negligible relative to clinical decision limits, suggesting limited clinical impact. In contrast, LD, CK, AST, and FFA demonstrated substantial positive interference (β values indicating large shifts) starting immediately at H-index 1, necessitating strict quality control even for mild hemolysis. Furthermore, we identified actionable thresholds for specific analytes where interference becomes clinically meaningful: K shows marked elevation at H-index ≧ 2, Mg at H-index ≧ 3, and Creatinine (Cr) shows significant negative bias at H-index≧ 3. These findings directly inform laboratory protocols. For instance, we recommend that for samples with H-index ≧ 2, the laboratory should withhold the report and contact the clinician for a re-draw, rather than releasing a potentially erroneous result that could misguide clinical decision-making.

Given the extensive number of analytes analyzed in this study, strict control over false discovery rates was essential to ensure the reliability of our statistical inferences. By applying the Benjamini-Hochberg (BH) correction to the initial screening (Kruskal-Wallis tests) and the conservative Bonferroni correction to post-hoc pairwise comparisons, we effectively minimized the risk of Type I errors (false positives). While these rigorous statistical adjustments inevitably reduced the significance of marginal associations, the relationships that remained significant are robust and highly likely to represent true biological interference caused by hemolysis.

The comprehensive model diagnostics provided further validation for our methodological choices and offered additional biological insights. Visual inspection of residual plots revealed distinct heteroscedasticity (non-constant variance) in the relationship between hemolysis indices and several analyte concentrations, providing strong statistical justification for employing quantile regression, which—unlike OLS—is robust to such distributional irregularities. The analysis of model fit statistics, specifically Koenker’s pseudo-R^2, highlighted the varying explanatory power of hemolysis across different tests. High pseudo-R^2 values were observed for markers such as LDH and K, indicating that hemolysis is the dominant driver of variance for these analytes; conversely, lower values for other analytes (such as RF) suggest that while hemolysis exerts a statistically significant effect, biological noise contribute more substantially to the observed variability. Additionally, the consistency of RMSE derived from 5-fold cross-validation confirms that the models possess stable generalization capabilities and are not subject to overfitting.

The limitations and strengths of this study are as follows: A limitation is that it solely investigates in vitro hemolysis, which may not be applicable to in vivo hemolysis scenarios. Additionally, severe hemolysis cases (H-index equal to 5) were not included in the analysis due to the small sample size. The advantages of this study include the introduction of a method using big data analysis to assess the impact and magnitude of hemolysis on laboratory analytes, with the development of open-source code providing a convenient analytical tool. Unlike previous studies that only provided point estimates, this study introduces the concept of sampling error through modeling, offering interval estimates for the impact of hemolysis on analytes. This provides more comprehensive evidence to support laboratory managers in devising rational strategies for managing hemolyzed samples. Furthermore, the study vividly and effectively demonstrates the influence and degree of hemolysis on analytes through appropriate visualization schemes. Lastly, the approach of big data analysis adopted in this study is also suitable for analyzing lipemia and jaundice, representing a more efficient and cost-effective analytical strategy.

## 5. Conclusion

Based on big data analysis, the impact of hemolysis on laboratory analytes can be effectively evaluated with a high level of consistency comparable to homogeneous experimental research.

## Supporting information

S1 File Clinical laboratory test index details, normality assessments, and hemolysis impact analyses of biomarkers. (DOCX)

S2 File Post-hoc tests. (CSV)

S3 File Model performance metrics. (CSV)

S4 File Residual plots. (PDF)

S1 Data Code for data analysis. (RMD)

S2 Data Code for data cleaning. (R)

## Abbreviations

CIs: Confidence Intervals
PVD: Patient Visit Department
H-index: Hemolysis index
ALT: Alanine Aminotransferase
TP: Total Protein
Alb: Albumin
TBil: Total Bilirubin
DBil: Direct Bilirubin
GGT: Gamma-Glutamyl Transferase
ALP: Alkaline Phosphatase
AST: Aspartate Aminotransferase
TBA: Total Bile Acid
CK: Creatine Kinase
LD: Lactate Dehydrogenase
ChE: Cholinesterase
K: Potassium
Na: Sodium
Cl: Chloride
TCO2: Total Carbon Dioxide
Ca: Calcium
Urea: Urea Nitrogen
Glu: Glucose
UA: Uric Acid
P: Phosphorus
TC: Total Cholesterol
TG: Triglycerides
HDL_C: High-Density Lipoprotein Cholesterol
LDL_C: Low-Density Lipoprotein Cholesterol
ApoA1: Apolipoprotein A1
ApoB: Apolipoprotein B
Lp(a): Lipoprotein(a)
hsCRP: High Sensitivity C-Reactive Protein
Mg: Magnesium
PA: Prealbumin
RF: Rheumatoid Factor
HCY: Homocysteine
IgG: Immunoglobulin G
IgA: Immunoglobulin A
IgM: Immunoglobulin M
ASO: Antistreptolysin O
CysC: Cystatin C
C3: Complement Component 3
C4: Complement Component 4
FFA: Free Fatty Acids
GA: Glycated Albumin
Cr(E): Creatinine
SI: Serum Iron
TRF: Transferrin
SF: Serum Ferritin
Sfa: Serum folic acid
VB12: Vitamin B12.
