# Supplementary material for: Real-world study: Assessing the impact of hemolysis on 48 biochemical and immunological analytes through big data analysis and its feasibility validation
Source: PLoS One. 2026 Jan 23;21(1):e0340265. doi: 10.1371/journal.pone.0340265 (PMC12829831; doi:10.1371/journal.pone.0340265)
Supplement: S1 File — (DOCX) [file pone.0340265.s001.docx]

**Supplementary materials**

Chaochao Ma^1,3a^, Xiaoqi Li^1a^, Wei Luo^1a^, Lian Hou^1^, Dandan Sun^1^, Li

Liu^1^, Xin Liu^1^, Ying Zhang^1^, Jingrong Xu^1^, Ling Qiu^1,2*^, Liangyu Xia^1*^

1 Department of Laboratory Medicine, Peking Union Medical College Hospital, Peking Union Medical College & Chinese Academy of Medical Science, Beijing 100730, PR China;

2 State Key Laboratory of Complex Severe and Rare Diseases, Peking Union Medical College Hospital, Peking Union Medical College & Chinese Academy of Medical Science, Beijing 100730, PR China;

3 Department of Occupational and Environmental Health Sciences, School of Public Health, Peking University, Beijing, 100191, China

**Running head:** **Big Data-Based Hemolysis Evaluation Method**

a These authors contributed equally to this work.

* Corresponding author.

1. mail address:

xlypumch@dingtalk.com (Liangyu Xia)

lingqiubjbigdata@126.com & qiul@pumch.cn (Ling Qiu).

**Supplementary Table 1 Comprehensive Clinical Laboratory Test Index: Methods, Quality Control Levels, and Measurement Units**

| **Test_Name** | **Methods** | **Quality control level** | **Units** |
| --- | --- | --- | --- |
| ALT | Enzymatic Method | 2 | U/L |
| TP | Biuret Method | 2 | g/L |
| Alb | Bromocresol Green Method | 2 | g/L |
| TBil | Vanadate Oxidation Method | 2 | μmol/L |
| DBil | Vanadate Oxidation Method | 2 | μmol/L |
| GGT | Enzymatic Method | 2 | U/L |
| ALP | Enzymatic Method | 2 | U/L |
| AST | Enzymatic Method | 2 | U/L |
| TBA | Enzyme Cycling Method | 2 | μmol/L |
| CK | Enzymatic Method | 2 | U/L |
| LD | Enzymatic Method | 2 | U/L |
| ChE | Enzymatic Method | 2 | KU/L |
| K | Indirect Ion-Selective Electrode Method | 2 | mmol/L |
| Na | Indirect Ion-Selective Electrode Method | 2 | mmol/L |
| Cl | Indirect Ion-Selective Electrode Method | 2 | mmol/L |
| TCO2 | Enzymatic Method | 2 | mmol/L |
| Ca | Arsenazo III Method | 2 | mmol/L |
| Urea | Urease Method | 2 | mmol/L |
| Glu | Hexokinase Method | 2 | mmol/L |
| UA | Uricase Method | 2 | μmol/L |
| P | Phosphomolybdic Acid Ultraviolet Spectrophotometry | 2 | mmol/L |
| TC | Cholesterol Oxidase Method | 2 | mmol/L |
| TG | Glycerophosphate Oxidase Method | 2 | mmol/L |
| HDL_C | Homogeneous Enzymatic Colorimetry | 2 | mmol/L |
| LDL_C | Homogeneous Enzymatic Colorimetry | 2 | mmol/L |
| ApoA1 | Immunoturbidimetric Assay | 2 | g/L |
| ApoB | Immunoturbidimetric Assay | 2 | g/L |
| Lp(a) | Latex Agglutination Turbidimetry | 2 | mg/L |
| hsCRP | Immunoturbidimetric Assay | 2 | mg/L |
| Mg | Dimethylbenzene Blue Colorimetry | 2 | mmol/L |
| PA | Immunoturbidimetric Assay | 2 | mg/L |
| RF | Immunoturbidimetric Assay | 2 | IU/ml |
| HCY | Enzyme Cycling Method | 2 | μmol/L |
| IgG | Immunoturbidimetric Assay | 2 | g/L |
| IgA | Immunoturbidimetric Assay | 2 | g/L |
| IgM | Immunoturbidimetric Assay | 2 | g/L |
| ASO | Immunoturbidimetric Assay | 2 | IU/ml |
| CysC | Latex Enhanced Immunoturbidimetry | 2 | mg/L |
| C3 | Immunoturbidimetric Assay | 2 | g/L |
| C4 | Immunoturbidimetric Assay | 2 | g/L |
| FFA | Enzymatic Method | 2 | μmol/L |
| GA | Bromocresol Green Method | 2 | g/L |
| Cr(E) | Enzymatic Method | 2 | μmol/L |
| SI | 2,4,6-Tripyridyl-s-triazine Colorimetric Method | 2 | μg/dl |
| TRF | Immunoturbidimetric Assay | 2 | g/L |
| SF | Chemiluminescence Assay | 3 | ng/ml |
| Sfa | Chemiluminescence Assay | 3 | ng/ml |
| VB12 | Chemiluminescence Assay | 3 | pg/ml |

ALT, Alanine Aminotransferase; TP, Total Protein; Alb, Albumin; TBil, Total Bilirubin; DBil, Direct Bilirubin; GGT, Gamma-Glutamyl Transferase; ALP, Alkaline Phosphatase; AST, Aspartate Aminotransferase; TBA, Total Bile Acid; CK, Creatine Kinase; LD, Lactate Dehydrogenase; ChE, Cholinesterase; K, Potassium; Na, Sodium; Cl, Chloride; TCO2, Total Carbon Dioxide; Ca, Calcium; Urea, Urea Nitrogen; Glu, Glucose; UA, Uric Acid; P, Phosphorus; TC, Total Cholesterol; TG, Triglycerides; HDL_C, High-Density Lipoprotein Cholesterol; LDL_C, Low-Density Lipoprotein Cholesterol; ApoA1, Apolipoprotein A1; ApoB, Apolipoprotein B; Lp(a), Lipoprotein(a); hsCRP, High Sensitivity C-Reactive Protein; Mg, Magnesium; PA, Prealbumin; RF, Rheumatoid Factor; HCY, Homocysteine; IgG, Immunoglobulin G; IgA, Immunoglobulin A; IgM, Immunoglobulin M; ASO, Antistreptolysin O; CysC, Cystatin C; C3, Complement Component 3; C4, Complement Component 4; FFA, Free Fatty Acids; GA, Glycated Albumin; Cr(E), Creatinine; SI, Serum Iron; TRF, Transferrin; SF, Serum Ferritin; Sfa, Serum Folic Acid; VB12, Vitamin B12.

**Supplementary Table 2 Normality Test Results for Continuous Variables Across Four Hemolysis Degree Group**

| Variables | **H-index_0** | **H-index_1** | **H-index_2** | **H-index_3** | **H-index_4** |
| --- | --- | --- | --- | --- | --- |
| Age | <0.001 | <0.001 | <0.001 | <0.001 | <0.001 |
| ALT | <0.001 | <0.001 | <0.001 | <0.001 | <0.001 |
| TP | <0.001 | <0.001 | <0.001 | <0.001 | <0.001 |
| Alb | <0.001 | <0.001 | <0.001 | <0.001 | <0.001 |
| TBil | <0.001 | <0.001 | <0.001 | <0.001 | <0.001 |
| DBil | <0.001 | <0.001 | <0.001 | <0.001 | <0.001 |
| GGT | <0.001 | <0.001 | <0.001 | <0.001 | <0.001 |
| ALP | <0.001 | <0.001 | <0.001 | <0.001 | <0.001 |
| AST | <0.001 | <0.001 | <0.001 | <0.001 | <0.001 |
| TBA | <0.001 | <0.001 | <0.001 | <0.001 | <0.001 |
| CK | <0.001 | <0.001 | <0.001 | <0.001 | <0.001 |
| LD | <0.001 | <0.001 | <0.001 | <0.001 | <0.001 |
| ChE | <0.001 | <0.001 | <0.001 | 0.003 | 0.001 |
| K | <0.001 | <0.001 | <0.001 | <0.001 | 0.002 |
| Na | <0.001 | <0.001 | <0.001 | <0.001 | <0.001 |
| Cl | <0.001 | <0.001 | <0.001 | <0.001 | <0.001 |
| TCO2 | <0.001 | <0.001 | <0.001 | <0.001 | 0.005 |
| Ca | <0.001 | <0.001 | <0.001 | <0.001 | <0.001 |
| Urea | <0.001 | <0.001 | <0.001 | <0.001 | <0.001 |
| Glu | <0.001 | <0.001 | <0.001 | <0.001 | <0.001 |
| UA | <0.001 | <0.001 | <0.001 | <0.001 | 0.004 |
| P | <0.001 | <0.001 | <0.001 | <0.001 | <0.001 |
| TC | <0.001 | <0.001 | <0.001 | <0.001 | <0.001 |
| TG | <0.001 | <0.001 | <0.001 | <0.001 | <0.001 |
| HDL_C | <0.001 | <0.001 | <0.001 | <0.001 | 0.543 |
| LDL_C | <0.001 | <0.001 | <0.001 | <0.001 | <0.001 |
| ApoA1 | <0.001 | <0.001 | <0.001 | <0.001 | 0.008 |
| ApoB | <0.001 | <0.001 | <0.001 | <0.001 | <0.001 |
| Lp(a) | <0.001 | <0.001 | <0.001 | <0.001 | <0.001 |
| hsCRP | <0.001 | <0.001 | <0.001 | <0.001 | <0.001 |
| Mg | <0.001 | <0.001 | <0.001 | <0.001 | <0.001 |
| PA | <0.001 | <0.001 | <0.001 | <0.001 | 0.022 |
| RF | <0.001 | <0.001 | <0.001 | <0.001 | <0.001 |
| HCY | <0.001 | <0.001 | <0.001 | <0.001 | <0.001 |
| IgG | <0.001 | <0.001 | <0.001 | <0.001 | <0.001 |
| IgA | <0.001 | <0.001 | <0.001 | <0.001 | <0.001 |
| IgM | <0.001 | <0.001 | <0.001 | <0.001 | <0.001 |
| ASO | <0.001 | <0.001 | <0.001 | <0.001 | <0.001 |
| CysC | <0.001 | <0.001 | <0.001 | 0.14 | 0.013 |
| C3 | <0.001 | <0.001 | 0.004 | 0.057 | 0.037 |
| C4 | <0.001 | <0.001 | <0.001 | <0.001 | 0.004 |
| FFA | <0.001 | <0.001 | <0.001 | <0.001 | <0.001 |
| GA | <0.001 | <0.001 | <0.001 | 0.908 | 0.013 |
| Cr(E) | <0.001 | <0.001 | <0.001 | <0.001 | <0.001 |
| SI | <0.001 | <0.001 | <0.001 | <0.001 | <0.001 |
| TRF | 0.069 | <0.001 | 0.583 | 0.322 | 0.322 |
| SF | <0.001 | <0.001 | <0.001 | <0.001 | <0.001 |
| Sfa | <0.001 | <0.001 | <0.001 | 0.841 | 0.157 |
| VB12 | <0.001 | <0.001 | <0.001 | 0.038 | 0.041 |

ALT, Alanine Aminotransferase; TP, Total Protein; Alb, Albumin; TBil, Total Bilirubin; DBil, Direct Bilirubin; GGT, Gamma-Glutamyl Transferase; ALP, Alkaline Phosphatase; AST, Aspartate Aminotransferase; TBA, Total Bile Acid; CK, Creatine Kinase; LD, Lactate Dehydrogenase; ChE, Cholinesterase; K, Potassium; Na, Sodium; Cl, Chloride; TCO2, Total Carbon Dioxide; Ca, Calcium; Urea, Urea Nitrogen; Glu, Glucose; UA, Uric Acid; P, Phosphorus; TC, Total Cholesterol; TG, Triglycerides; HDL_C, High-Density Lipoprotein Cholesterol; LDL_C, Low-Density Lipoprotein Cholesterol; ApoA1, Apolipoprotein A1; ApoB, Apolipoprotein B; Lp(a), Lipoprotein(a); hsCRP, High Sensitivity C-Reactive Protein; Mg, Magnesium; PA, Prealbumin; RF, Rheumatoid Factor; HCY, Homocysteine; IgG, Immunoglobulin G; IgA, Immunoglobulin A; IgM, Immunoglobulin M; ASO, Antistreptolysin O; CysC, Cystatin C; C3, Complement Component 3; C4, Complement Component 4; FFA, Free Fatty Acids; GA, Glycated Albumin; Cr(E), Creatinine; SI, Serum Iron; TRF, Transferrin; SF, Serum Ferritin; Sfa, Serum Folic Acid; VB12, Vitamin B12.When the P-value is less than 0.001, it indicates a significant departure from normality in the statistical analysis of the data.

**Supplementary Table 3 Univariate Analysis of the Impact of Hemolysis on Biochemical and Immunological Biomarkers Excluding Participants Under 18 Years of Age**

| **Makers** | **H-index_0** | **H-index_1** | **H-index_2** | **H-index_3** | **H-index_4** | **P_Value** |
| --- | --- | --- | --- | --- | --- | --- |
| **ALT** | 18.00 (15.00) | 20.00 (18.00) | 21.00 (17.00) | 21.00 (16.00) | 22.00 (17.00) | <0.001 |
| **TP** | 72.00 (7.00) | 73.00 (7.00) | 73.00 (8.00) | 74.00 (9.00) | 75.00 (8.00) | <0.001 |
| **Alb** | 44.00 (4.00) | 44.00 (6.00) | 44.00 (6.00) | 45.00 (6.00) | 45.00 (7.00) | <0.001 |
| **TBil** | 10.80 (5.90) | 11.30 (6.20) | 12.20 (6.40) | 13.70 (7.10) | 14.35 (7.48) | <0.001 |
| **DBil** | 3.30 (1.90) | 2.80 (1.80) | 2.70 (1.70) | 2.80 (1.60) | 2.90 (1.90) | <0.001 |
| **GGT** | 23.00 (22.00) | 26.00 (26.00) | 25.00 (27.00) | 26.00 (26.00) | 27.00 (30.25) | <0.001 |
| **ALP** | 75.00 (33.00) | 77.00 (35.00) | 76.00 (35.00) | 74.00 (41.00) | 71.00 (35.50) | <0.001 |
| **AST** | 21.00 (10.00) | 27.00 (12.00) | 33.00 (15.00) | 41.00 (15.00) | 49.00 (20.00) | <0.001 |
| **TBA** | 2.20 (2.60) | 2.30 (2.90) | 2.40 (3.00) | 2.20 (2.60) | 2.45 (2.97) | 0.033 |
| **CK** | 87.00 (68.00) | 92.00 (75.00) | 95.00 (66.25) | 108.50 (66.50) | 136.50 (116.50) | <0.001 |
| **LD** | 183.00 (54.00) | 273.00 (72.00) | 361.00 (102.00) | 482.00 (103.00) | 632.00 (153.00) | <0.001 |
| **ChE** | 7.70 (2.40) | 7.80 (2.60) | 7.60 (2.60) | 7.70 (2.50) | 7.40 (2.50) | <0.001 |
| **K** | 4.20 (0.50) | 4.40 (0.50) | 4.70 (0.50) | 5.10 (0.70) | 5.40 (0.67) | <0.001 |
| **Na** | 140.00 (3.00) | 139.00 (3.00) | 139.00 (3.00) | 138.00 (3.00) | 138.00 (3.00) | <0.001 |
| **Cl** | 105.00 (3.00) | 104.00 (3.00) | 104.00 (4.00) | 104.00 (4.00) | 104.00 (4.00) | <0.001 |
| **TCO2** | 26.90 (3.40) | 26.10 (3.60) | 26.00 (3.60) | 25.80 (3.90) | 25.50 (3.55) | <0.001 |
| **Ca** | 2.33 (0.15) | 2.34 (0.15) | 2.32 (0.15) | 2.31 (0.16) | 2.28 (0.19) | <0.001 |
| **Urea** | 4.95 (2.13) | 5.01 (2.10) | 5.06 (2.24) | 4.94 (2.09) | 5.13 (2.31) | <0.001 |
| **Glu** | 5.30 (1.10) | 5.30 (1.20) | 5.30 (1.20) | 5.20 (1.30) | 5.20 (1.30) | <0.001 |
| **UA** | 314.00 (124.00) | 318.00 (128.00) | 308.00 (130.00) | 305.50 (123.00) | 295.00 (128.50) | <0.001 |
| **P** | 1.21 (0.25) | 1.23 (0.25) | 1.27 (0.25) | 1.29 (0.25) | 1.36 (0.27) | <0.001 |
| **TC** | 4.65 (1.44) | 4.71 (1.49) | 4.75 (1.52) | 4.68 (1.52) | 4.63 (1.57) | 0.002 |
| **TG** | 1.24 (0.99) | 1.34 (1.10) | 1.28 (1.05) | 1.25 (0.91) | 1.28 (1.07) | <0.001 |
| **HDL_C** | 1.21 (0.44) | 1.17 (0.42) | 1.19 (0.45) | 1.17 (0.44) | 1.17 (0.46) | <0.001 |
| **LDL_C** | 2.71 (1.16) | 2.76 (1.25) | 2.74 (1.24) | 2.71 (1.27) | 2.83 (1.27) | <0.001 |
| **ApoA1** | 1.36 (0.31) | 1.34 (0.31) | 1.36 (0.32) | 1.32 (0.32) | 1.29 (0.32) | 0.009 |
| **ApoB** | 0.88 (0.31) | 0.92 (0.35) | 0.91 (0.34) | 0.91 (0.35) | 0.90 (0.40) | <0.001 |
| **Lp(a)** | 90.00 (168.00) | 88.00 (161.75) | 91.00 (174.00) | 103.00 (223.00) | 95.00 (114.50) | 0.256 |
| **hsCRP** | 1.33 (3.62) | 1.62 (4.31) | 1.79 (5.07) | 1.50 (4.63) | 1.40 (4.36) | <0.001 |
| **Mg** | 0.87 (0.10) | 0.89 (0.10) | 0.91 (0.10) | 0.94 (0.10) | 0.94 (0.12) | <0.001 |
| **PA** | 261.00 (75.00) | 263.00 (78.00) | 257.80 (84.00) | 250.50 (75.00) | 253.00 (77.25) | <0.001 |
| **RF** | 7.70 (30.70) | 7.00 (40.50) | 7.60 (41.50) | 3.00 (16.00) | 14.90 (31.00) | <0.001 |
| **HCY** | 12.60 (4.90) | 13.20 (4.80) | 13.40 (5.40) | 13.80 (6.50) | 13.30 (6.45) | 0.001 |
| **IgG** | 12.03 (5.23) | 11.70 (5.36) | 11.89 (6.14) | 11.70 (4.64) | 11.87 (3.83) | 0.107 |
| **IgA** | 2.21 (1.46) | 2.18 (1.47) | 2.24 (1.55) | 2.32 (1.56) | 2.26 (1.38) | 0.797 |
| **IgM** | 0.90 (0.71) | 0.89 (0.76) | 0.89 (0.82) | 0.97 (0.78) | 0.78 (0.95) | 0.178 |
| **ASO** | 65.20 (77.97) | 58.00 (74.75) | 50.00 (54.55) | 37.00 (24.80) | 89.00 (150.93) | 0.039 |
| **CysC** | 0.88 (0.40) | 0.99 (0.36) | 0.96 (0.58) | 0.84 (0.07) | 0.83 (0.54) | 0.088 |
| **C3** | 1.01 (0.34) | 1.03 (0.37) | 1.03 (0.36) | 0.94 (0.45) | 0.98 (0.32) | 0.136 |
| **C4** | 0.18 (0.09) | 0.18 (0.10) | 0.18 (0.10) | 0.16 (0.11) | 0.17 (0.09) | 0.644 |
| **FFA** | 509.00 (296.00) | 582.00 (316.75) | 619.50 (312.00) | 646.00 (320.00) | 745.00 (308.00) | <0.001 |
| **GA** | 6.25 (2.83) | 6.44 (2.73) | 5.98 (3.17) | 6.41 (2.34) | 5.11 (4.72) | 0.078 |
| **Cr(E)** | 66.00 (23.00) | 65.00 (24.00) | 62.00 (24.00) | 60.00 (25.50) | 59.00 (28.90) | <0.001 |
| **SI** | 76.00 (66.05) | 76.00 (62.00) | 76.00 (65.20) | 73.40 (52.47) | 76.00 (69.00) | 0.521 |
| **TRF** | 2.39 (0.89) | 2.32 (0.82) | 2.26 (0.79) | 2.41 (1.02) | 2.54 (0.63) | 0.014 |
| **SF** | 69.00 (239.00) | 106.00 (310.00) | 98.00 (335.50) | 79.50 (224.25) | 122.00 (411.25) | <0.001 |
| **Sfa** | 9.40 (6.80) | 9.70 (6.57) | 10.10 (6.30) | 11.80 (4.60) | 11.25 (3.35) | 0.007 |
| **VB12** | 355.00 (267.00) | 381.00 (291.25) | 380.00 (330.50) | 520.00 (427.50) | 381.00 (270.00) | 0.015 |

ALT, Alanine Aminotransferase; TP, Total Protein; Alb, Albumin; TBil, Total Bilirubin; DBil, Direct Bilirubin; GGT, Gamma-Glutamyl Transferase; ALP, Alkaline Phosphatase; AST, Aspartate Aminotransferase; TBA, Total Bile Acid; CK, Creatine Kinase; LD, Lactate Dehydrogenase; ChE, Cholinesterase; K, Potassium; Na, Sodium; Cl, Chloride; TCO2, Total Carbon Dioxide; Ca, Calcium; Urea, Urea Nitrogen; Glu, Glucose; UA, Uric Acid; P, Phosphorus; TC, Total Cholesterol; TG, Triglycerides; HDL_C, High-Density Lipoprotein Cholesterol; LDL_C, Low-Density Lipoprotein Cholesterol; ApoA1, Apolipoprotein A1; ApoB, Apolipoprotein B; Lp(a), Lipoprotein(a); hsCRP, High Sensitivity C-Reactive Protein; Mg, Magnesium; PA, Prealbumin; RF, Rheumatoid Factor; HCY, Homocysteine; IgG, Immunoglobulin G; IgA, Immunoglobulin A; IgM, Immunoglobulin M; ASO, Antistreptolysin O; CysC, Cystatin C; C3, Complement Component 3; C4, Complement Component 4; FFA, Free Fatty Acids; GA, Glycated Albumin; Cr(E), Creatinine; SI, Serum Iron; TRF, Transferrin; SF, Serum Ferritin; Sfa, Serum Folic Acid; VB12, Vitamin B12.
