# Supplementary material for: Real-world study: Assessing the impact of hemolysis on 48 biochemical and immunological analytes through big data analysis and its feasibility validation
Source: PLoS One. 2026 Jan 23;21(1):e0340265. doi: 10.1371/journal.pone.0340265 (PMC12829831; doi:10.1371/journal.pone.0340265)
Supplement: S4 File — (PDF) [file pone.0340265.s004.pdf]

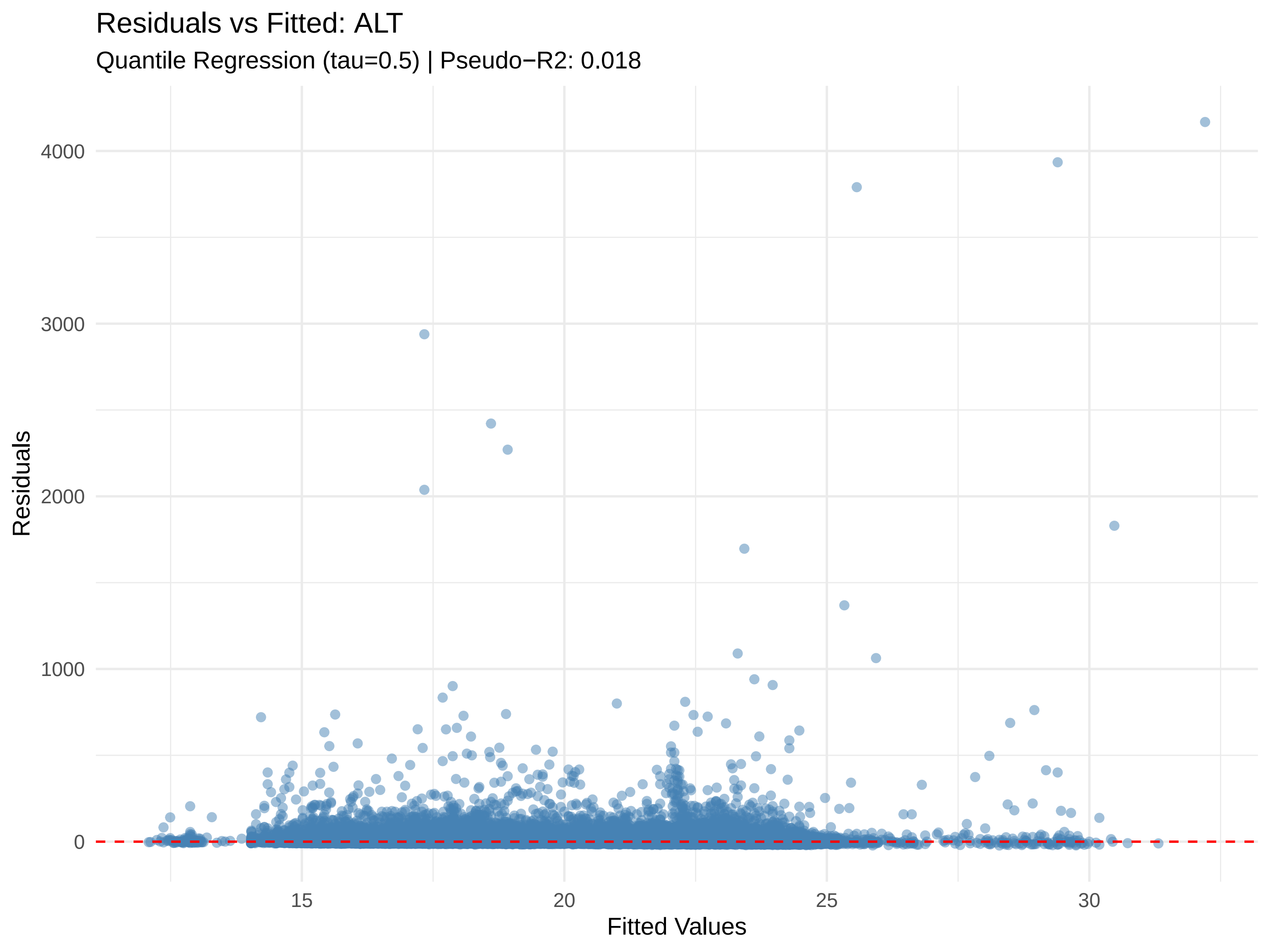
Residuals vs Fitted: ALT
Quantile Regression (tau=0.5) | Pseudo−R2: 0.018
4000
3000
2000
1000
0
15
20
25
30
Fitted Values
Residuals

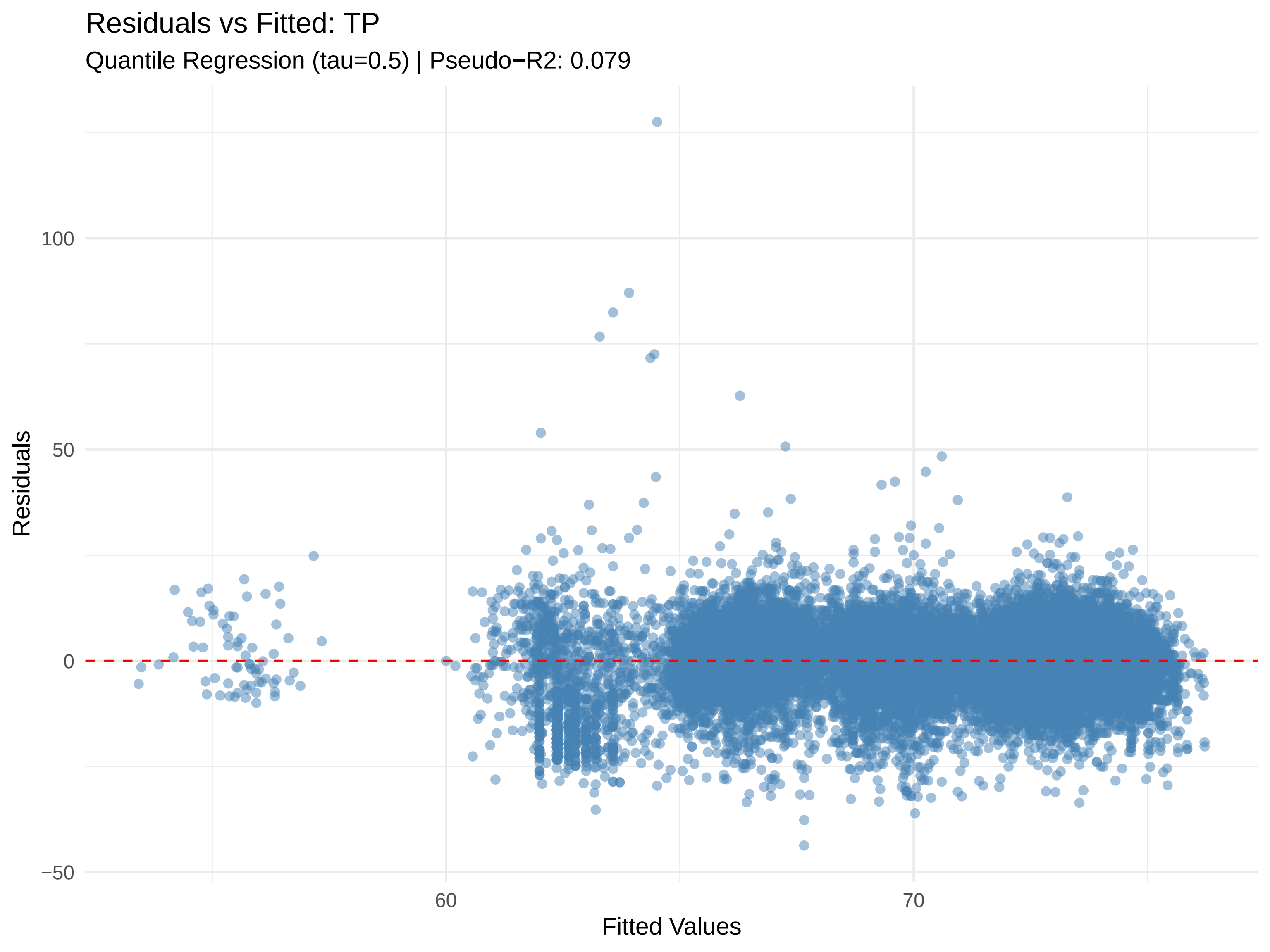

Residuals vs Fitted: TP
Quantile Regression (tau=0.5) | Pseudo−R2: 0.079
100
50
0
−50
60
70
Residuals
Fitted Values

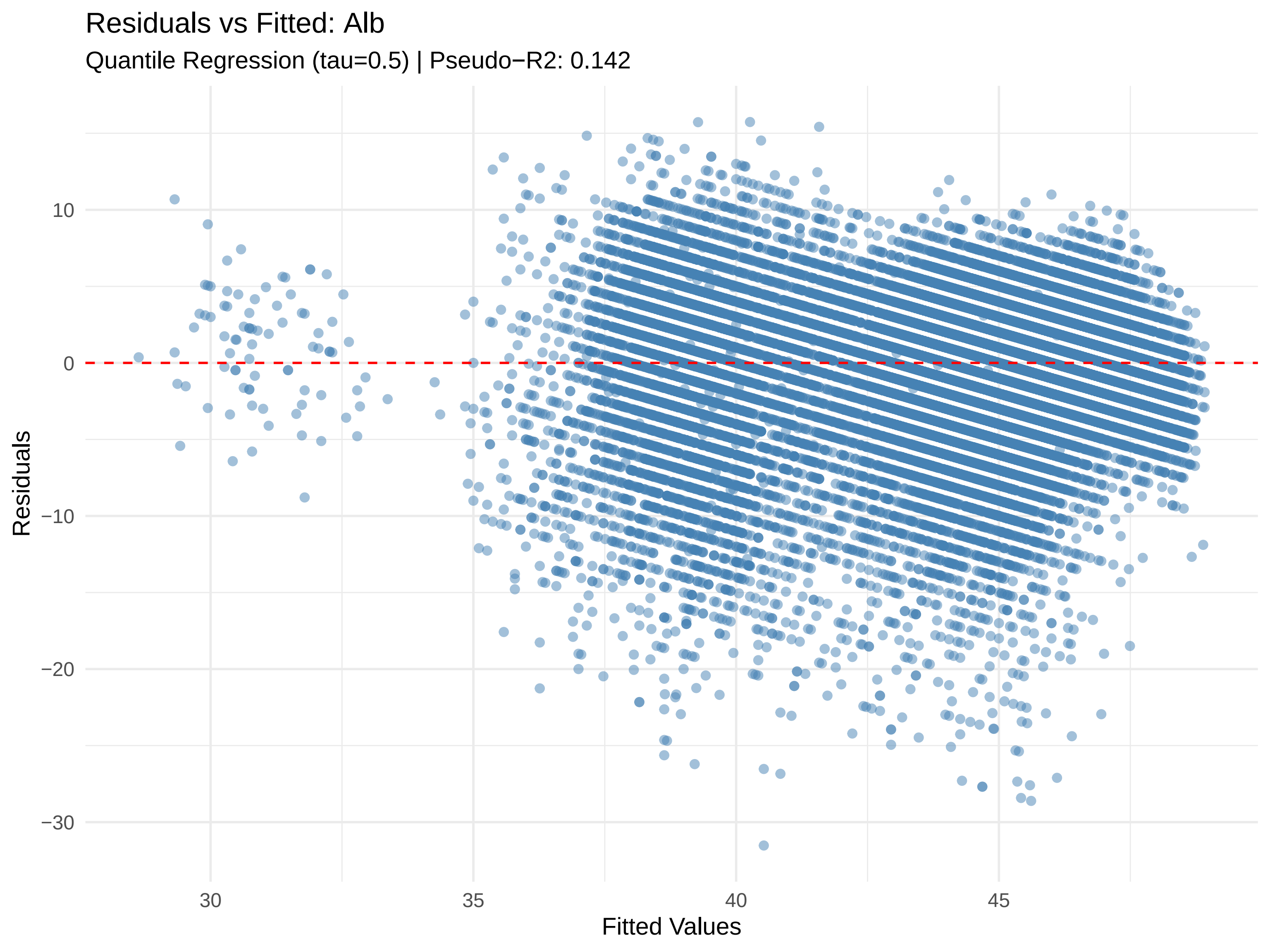

Residuals vs Fitted: Alb
Quantile Regression (tau=0.5) | Pseudo−R2: 0.142
Residuals
Fitted Values
10
0
−10
−20
−30
30
35
40
45

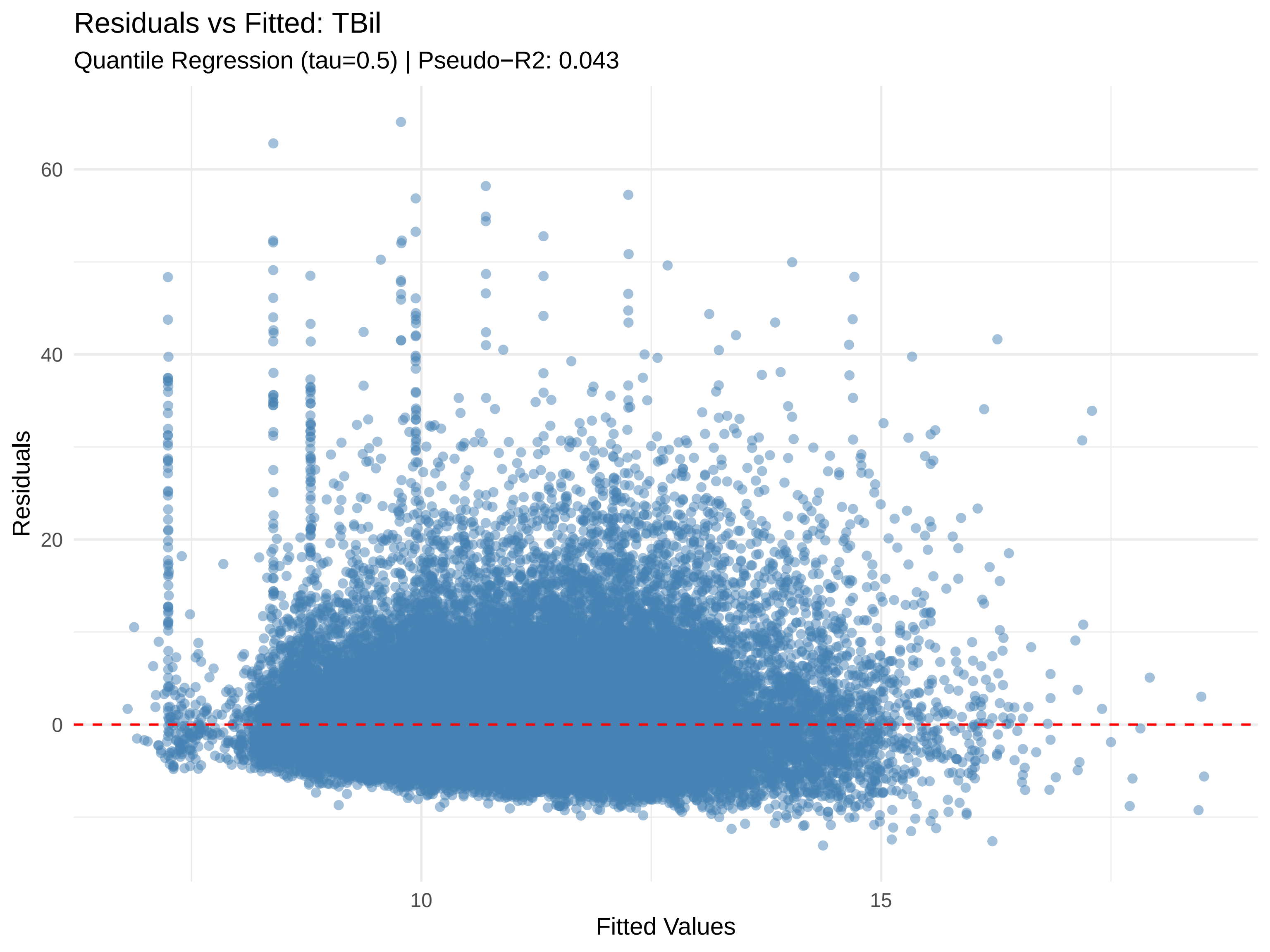
Residuals vs Fitted: TBil
Quantile Regression (tau=0.5) | Pseudo−R2: 0.043
60
40
20
0
Residuals
10
15
Fitted Values

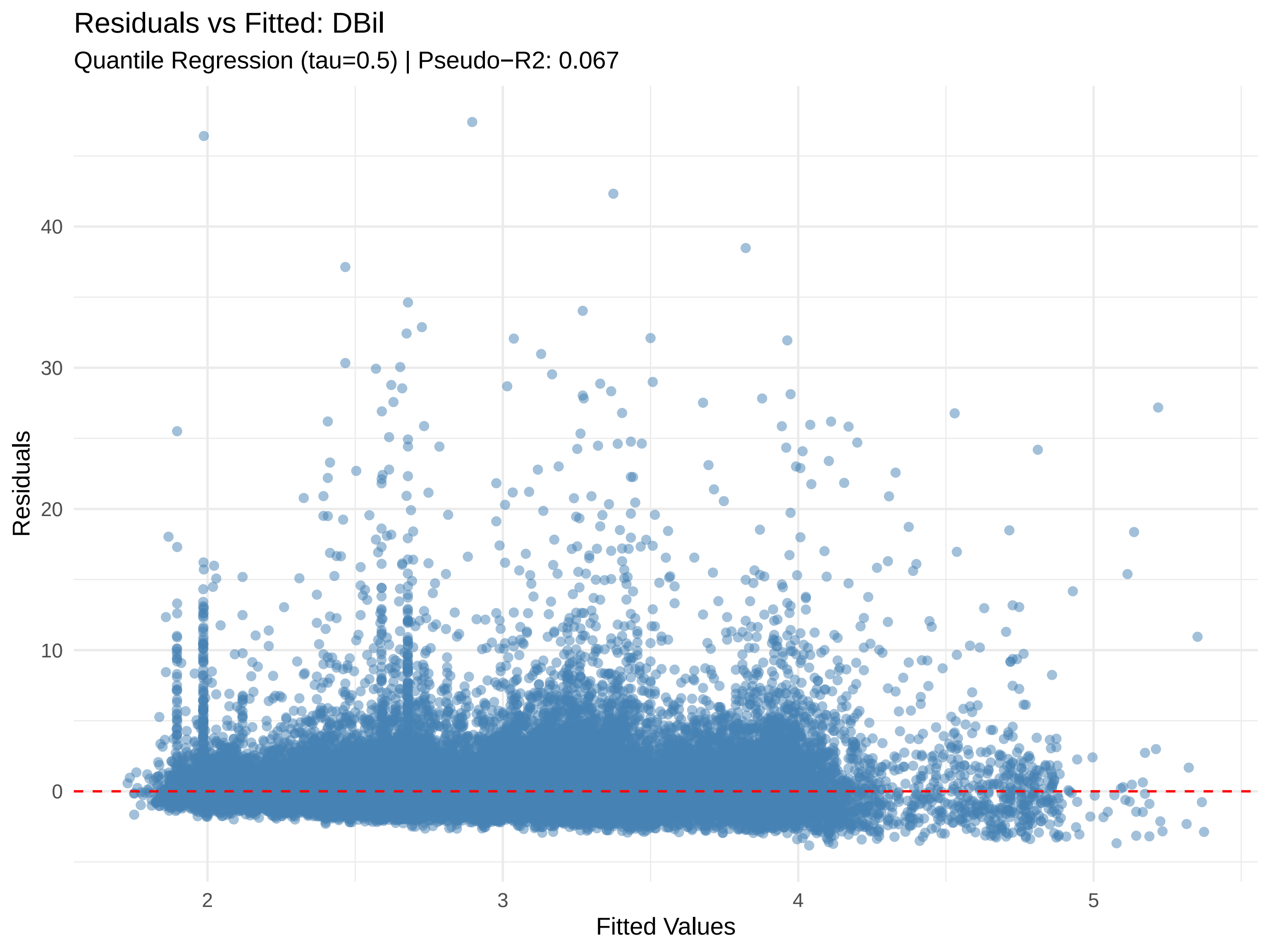
Residuals vs Fitted: DBil
Quantile Regression (tau=0.5) | Pseudo−R2: 0.067
Residuals
40
30
20
10
0
2
3
4
5
Fitted Values

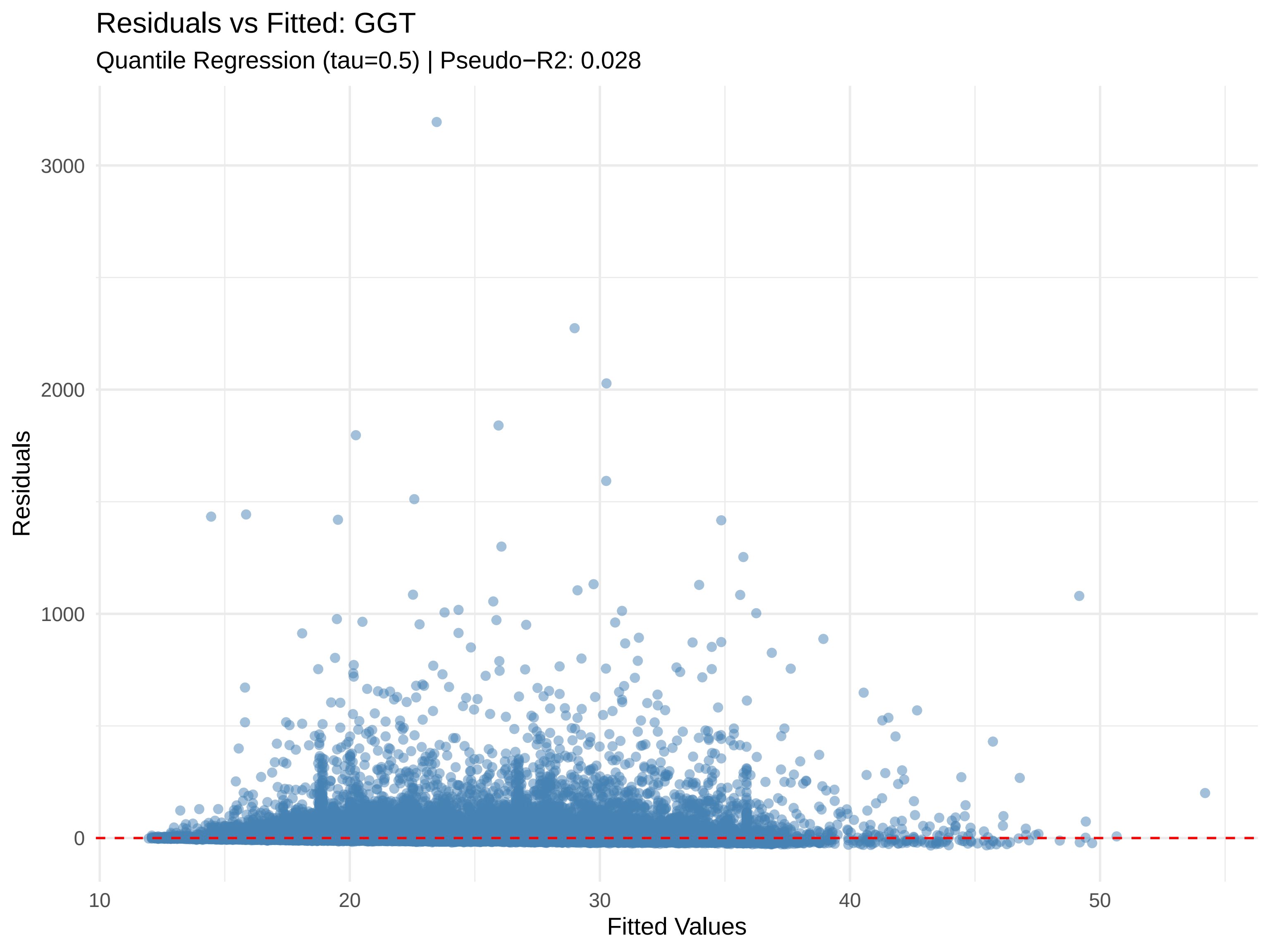

Residuals vs Fitted: GGT
Quantile Regression (tau=0.5) | Pseudo−R2: 0.028
Residuals
Fitted Values
3000
2000
1000
0
10
20
30
40
50

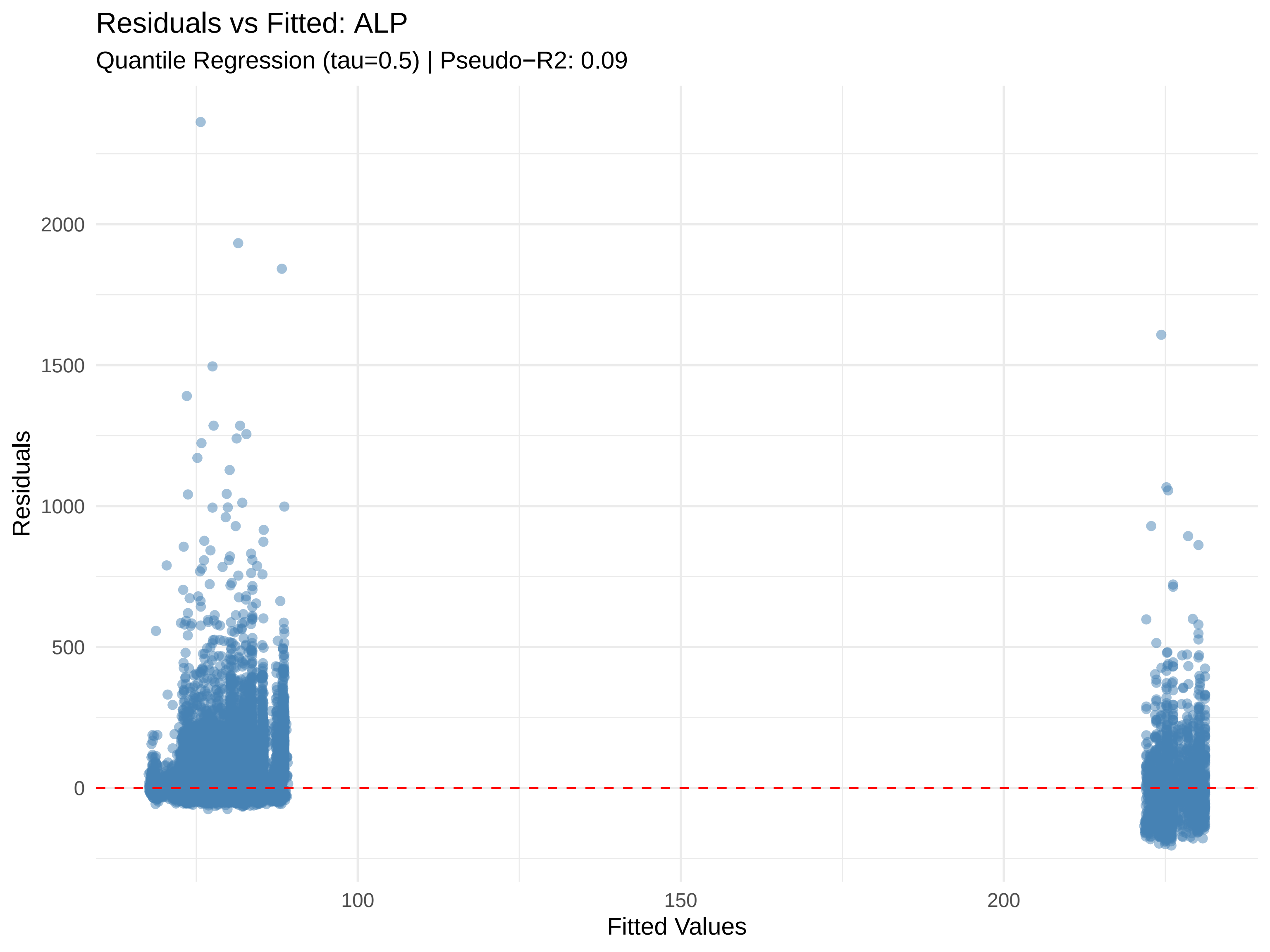
Residuals vs Fitted: ALP
Quantile Regression (tau=0.5) | Pseudo−R2: 0.09
Residuals
2000
1500
1000
500
0
100
150
200
Fitted Values

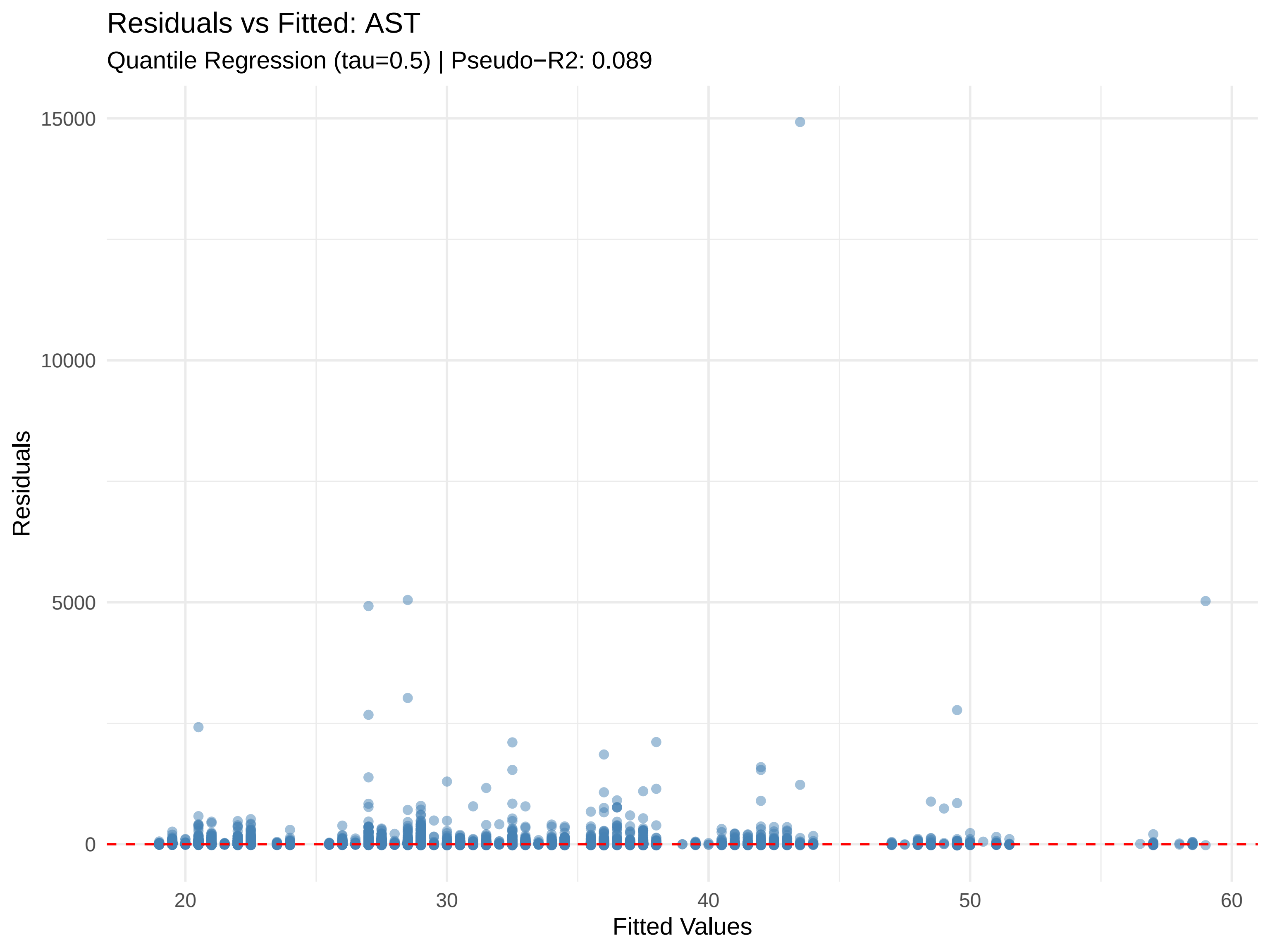
Residuals vs Fitted: AST
Quantile Regression (tau=0.5) | Pseudo−R2: 0.089
Residuals
15000
10000
5000
0
20
30
40
50
60
Fitted Values

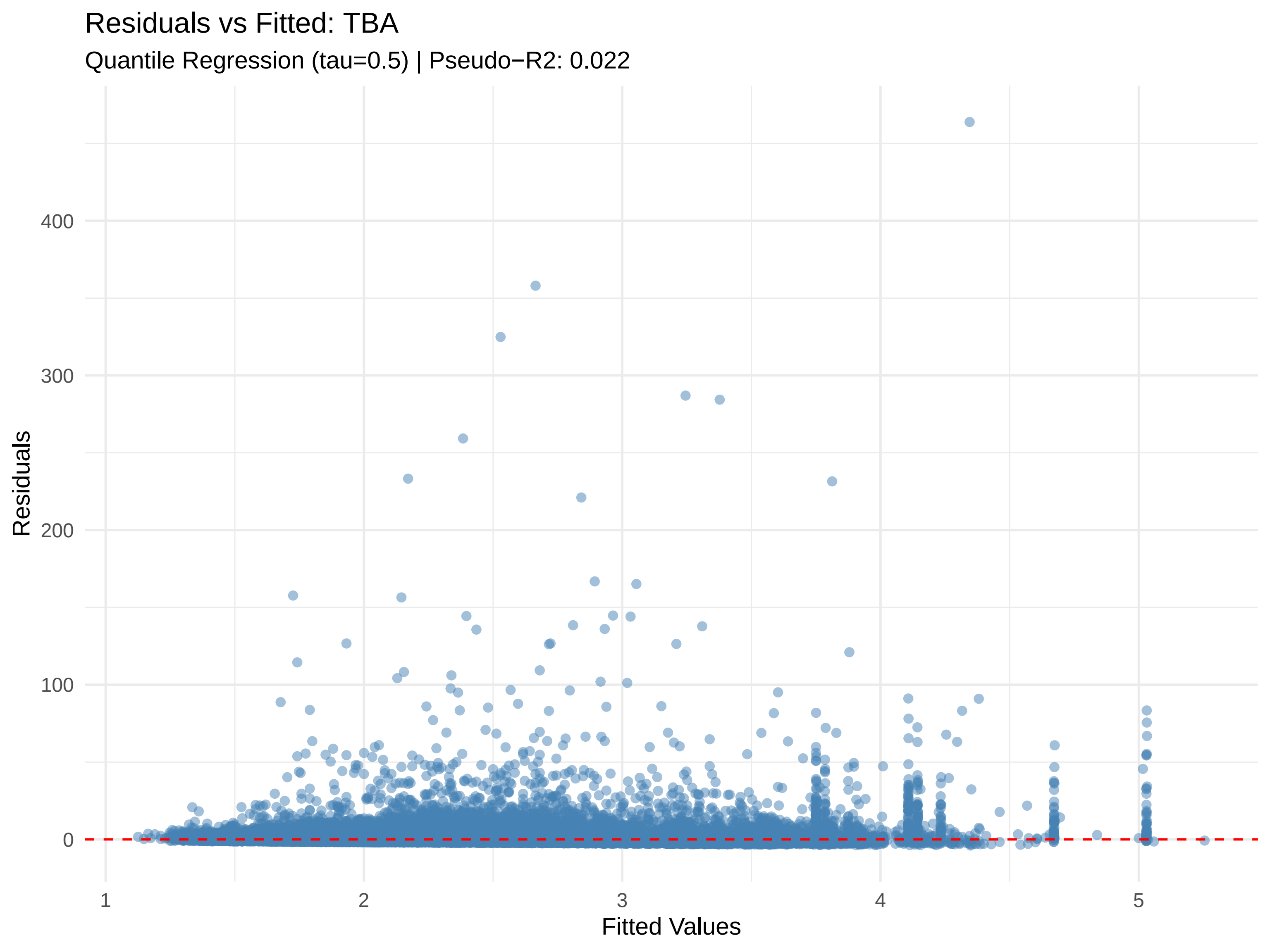

Residuals vs Fitted: TBA
Quantile Regression (tau=0.5) | Pseudo−R2: 0.022
Residuals
400
300
200
100
0
1
2
3
4
5
Fitted Values

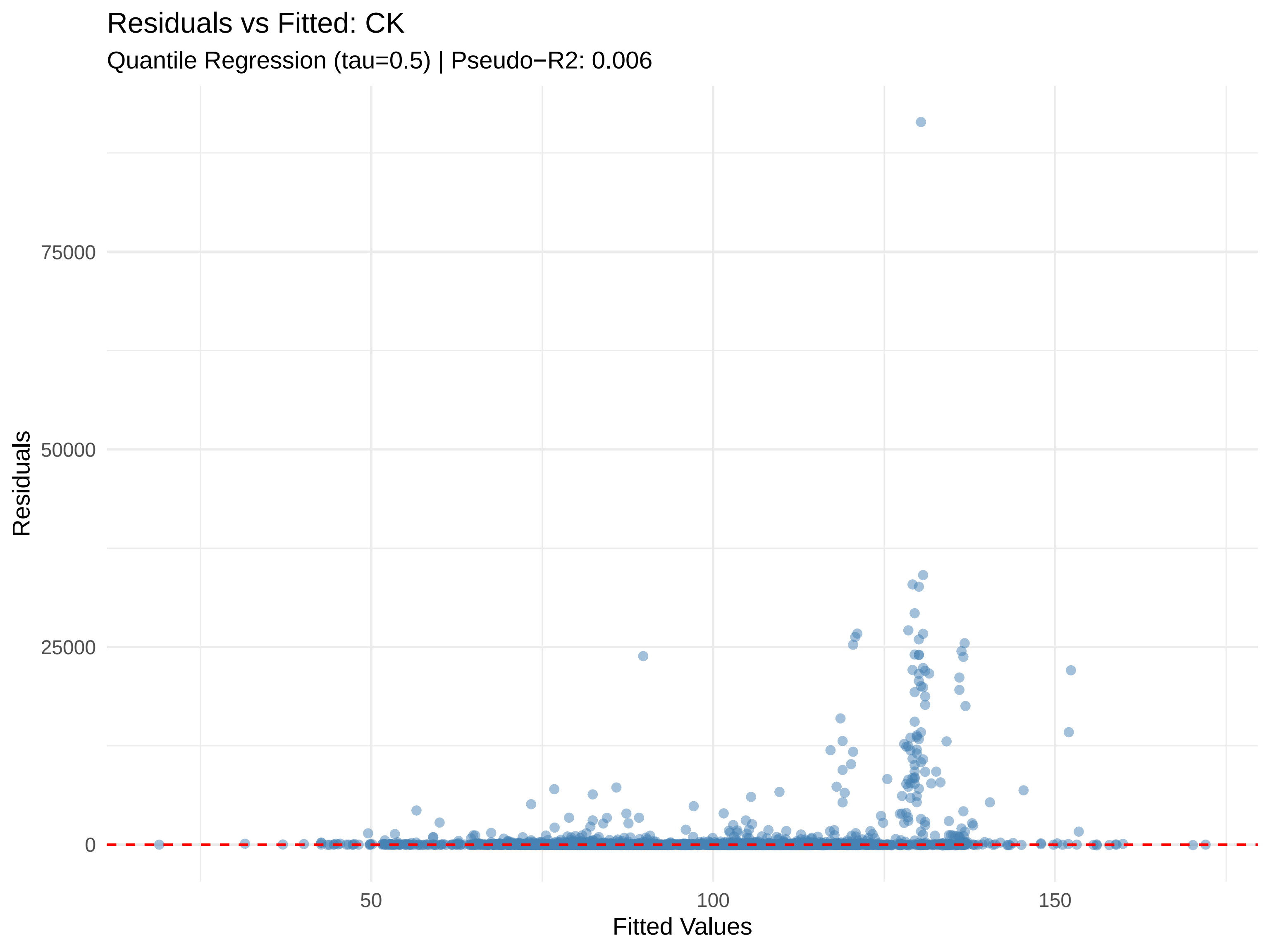
Residuals vs Fitted: CK
Quantile Regression (tau=0.5) | Pseudo−R2: 0.006
Residuals
75000
50000
25000
0
50
100
150
Fitted Values

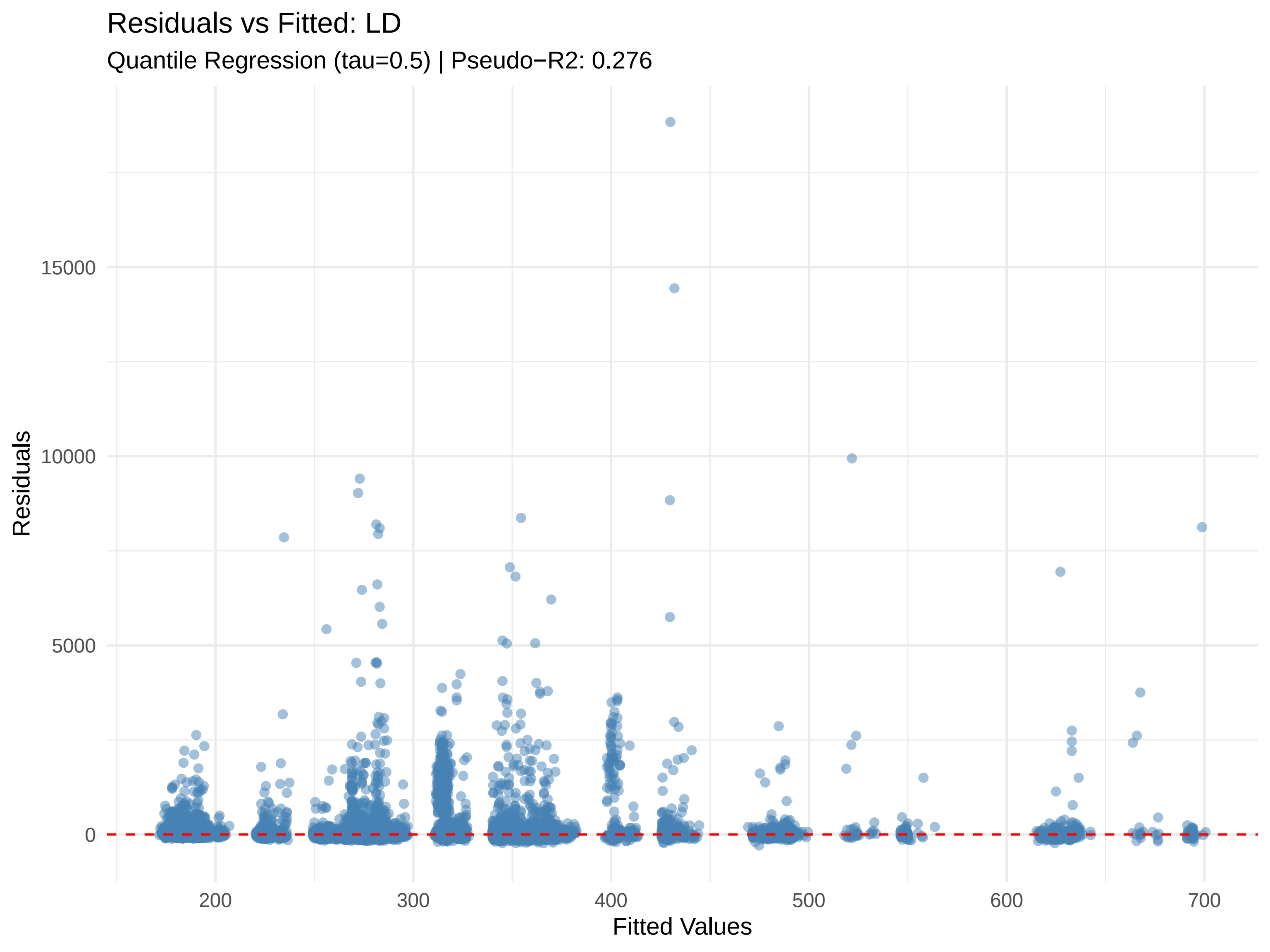
Residuals vs Fitted: LD
Quantile Regression (tau=0.5) | Pseudo−R2: 0.276
Residuals
15000
10000
5000
0
200
300
400
500
600
700
Fitted Values

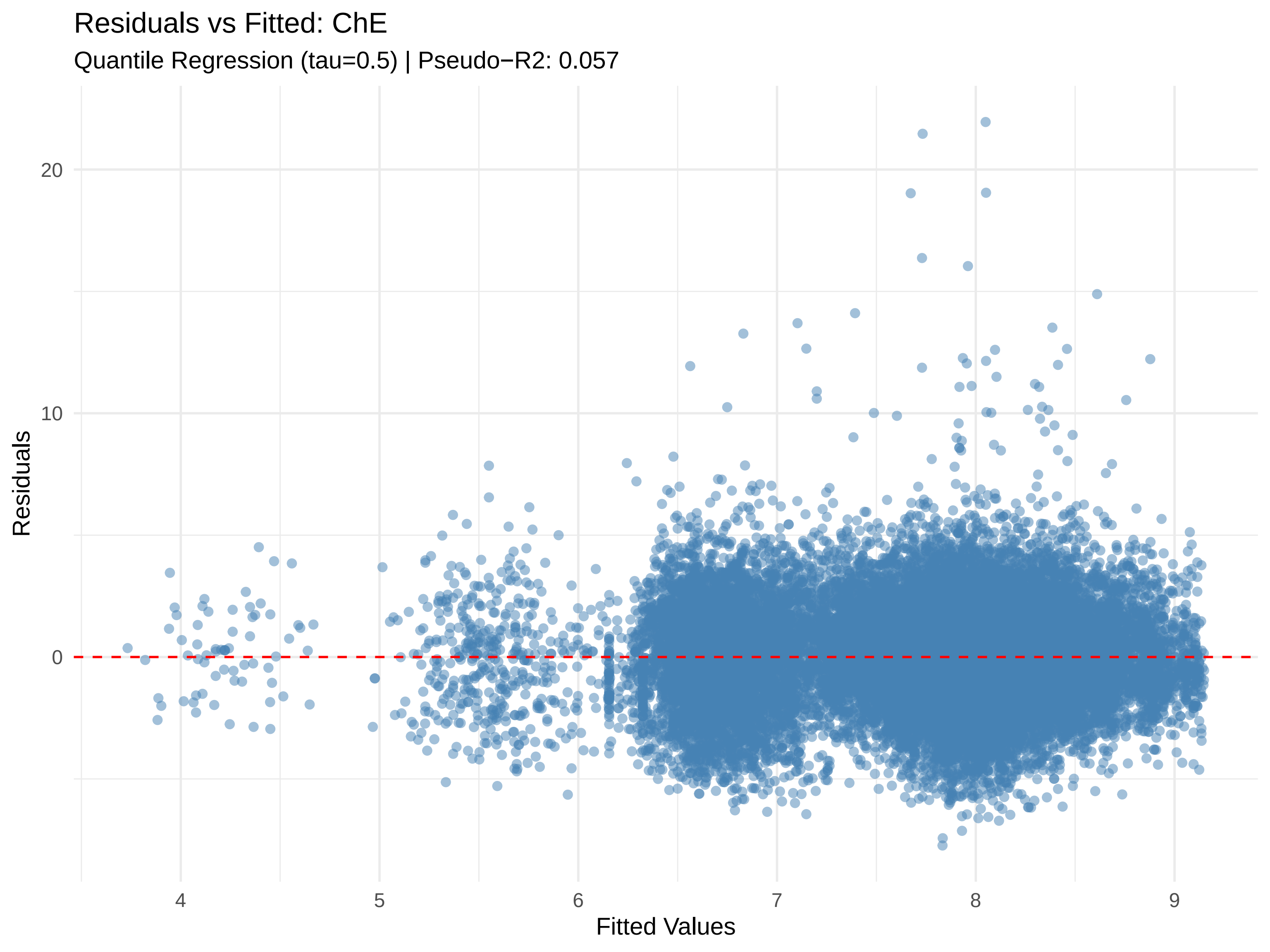
Residuals vs Fitted: ChE
Quantile Regression (tau=0.5) | Pseudo−R2: 0.057
Residuals
20
10
0
4
5
6
7
8
9
Fitted Values

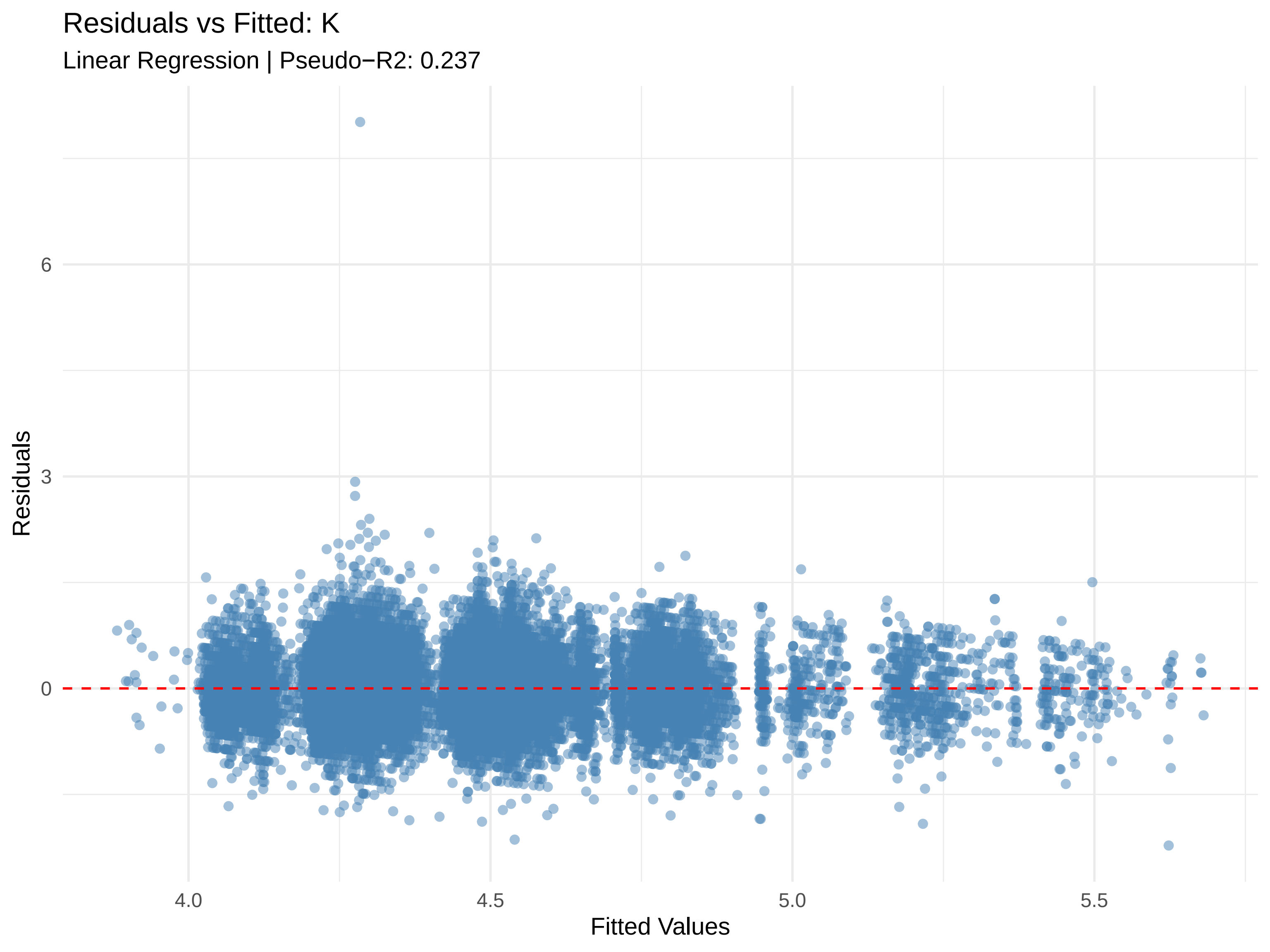

Residuals vs Fitted: K
Linear Regression | Pseudo−R2: 0.237
Residuals
6
3
0
4.0
4.5
5.0
5.5
Fitted Values

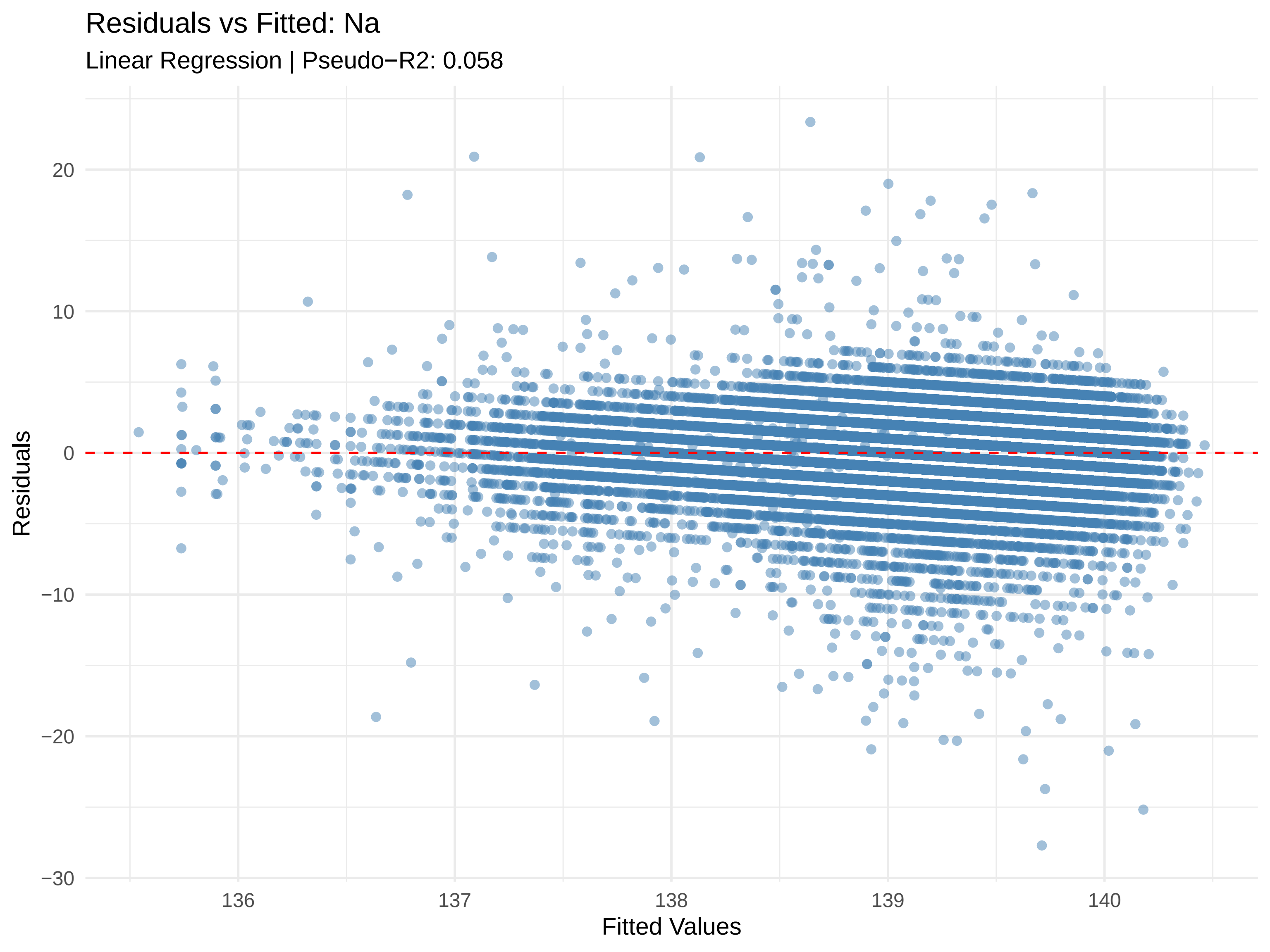

Residuals vs Fitted: Na
Linear Regression | Pseudo−R2: 0.058
Residuals
Fitted Values
20
10
0
−10
−20
−30
136
137
138
139
140

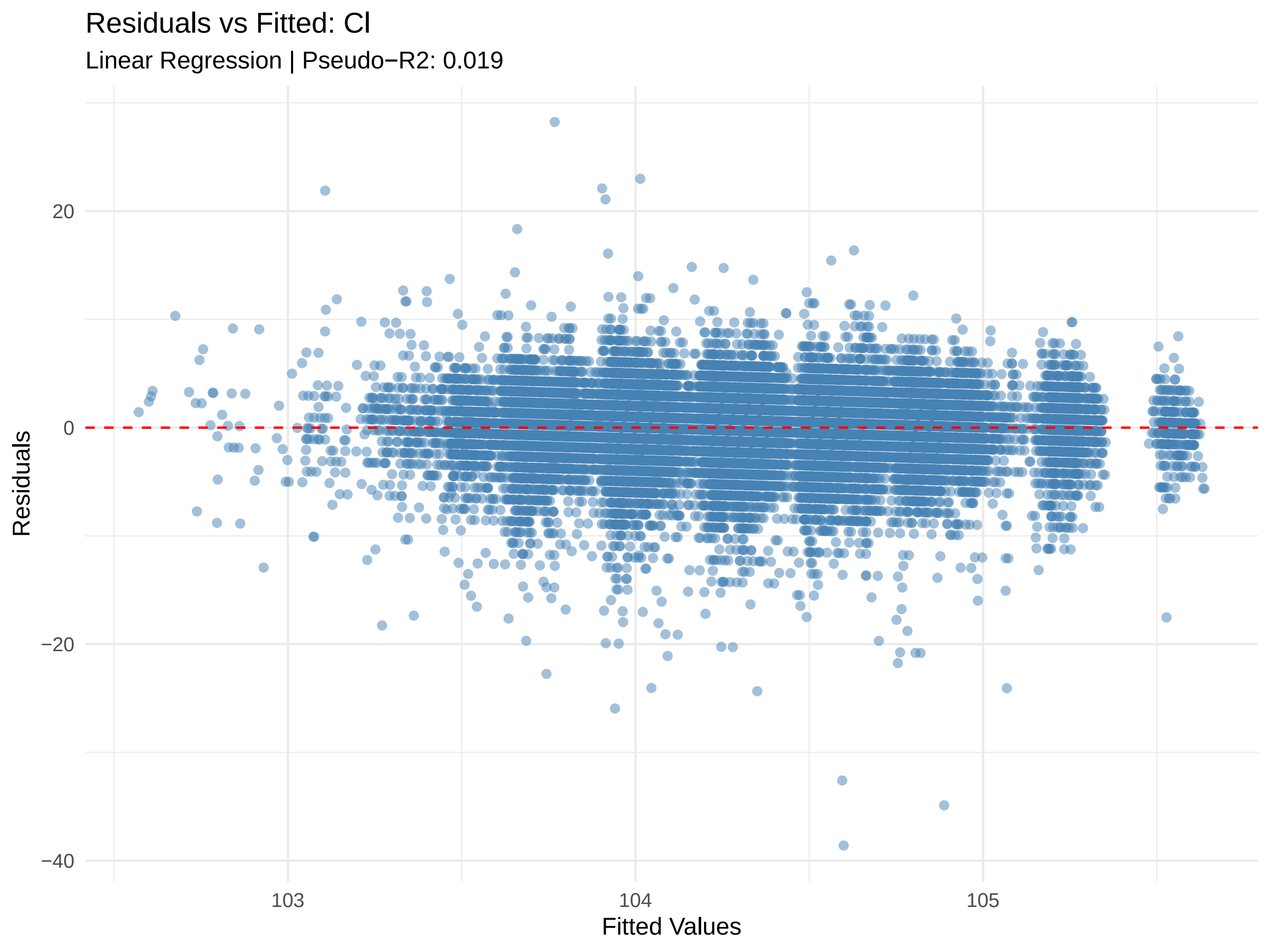
Residuals vs Fitted: CI
Linear Regression | Pseudo−R2: 0.019
Residuals
20
0
−20
−40
103
104
105
Fitted Values

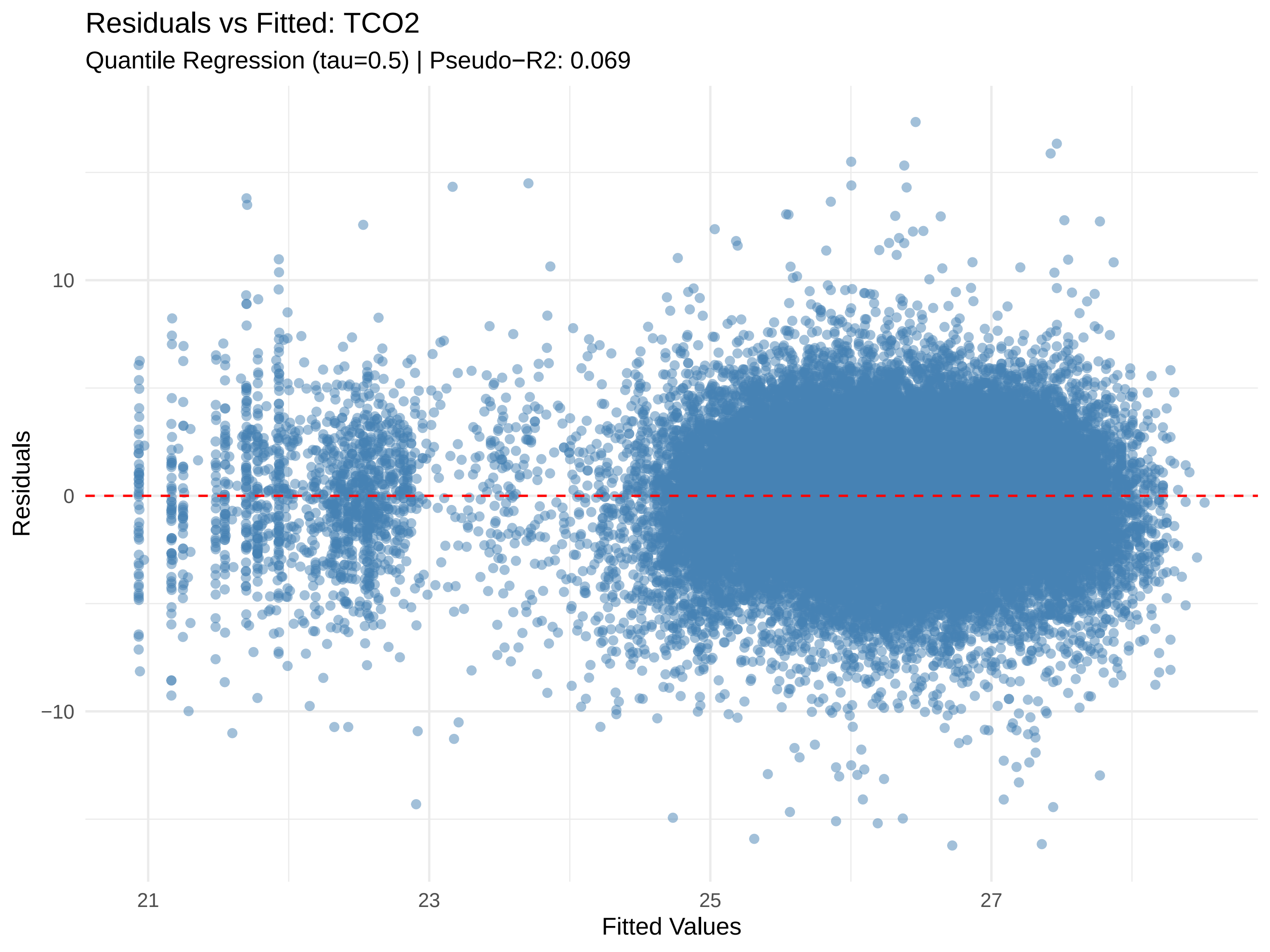
Residuals vs Fitted: TCO2
Quantile Regression (tau=0.5) | Pseudo−R2: 0.069
Residuals
10
0
−10
21
23
25
27
Fitted Values

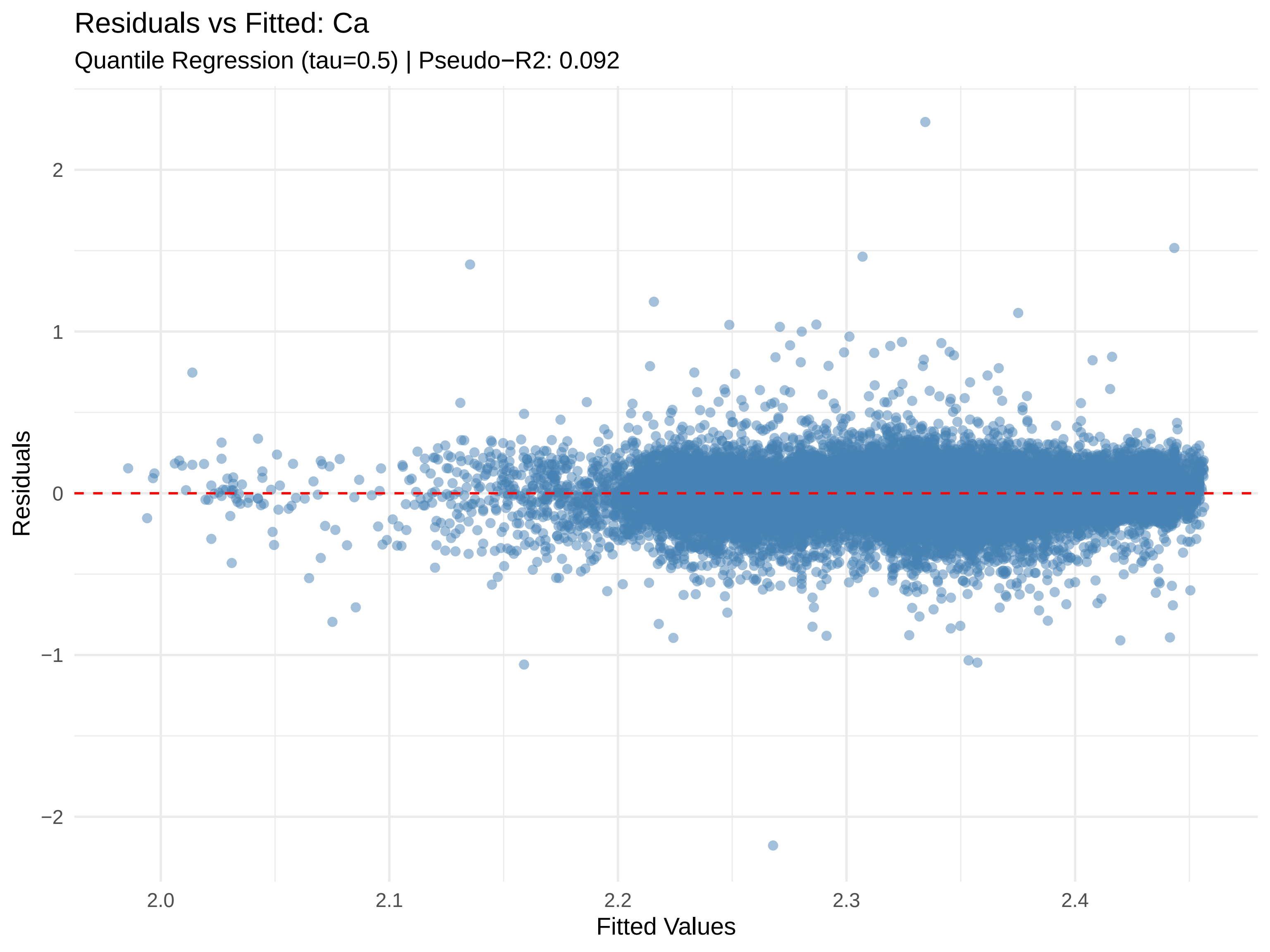

Residuals vs Fitted: Ca
Quantile Regression (tau=0.5) | Pseudo−R2: 0.092
Residuals
Fitted Values
2
1
0
−1
−2
2.0
2.1
2.2
2.3
2.4

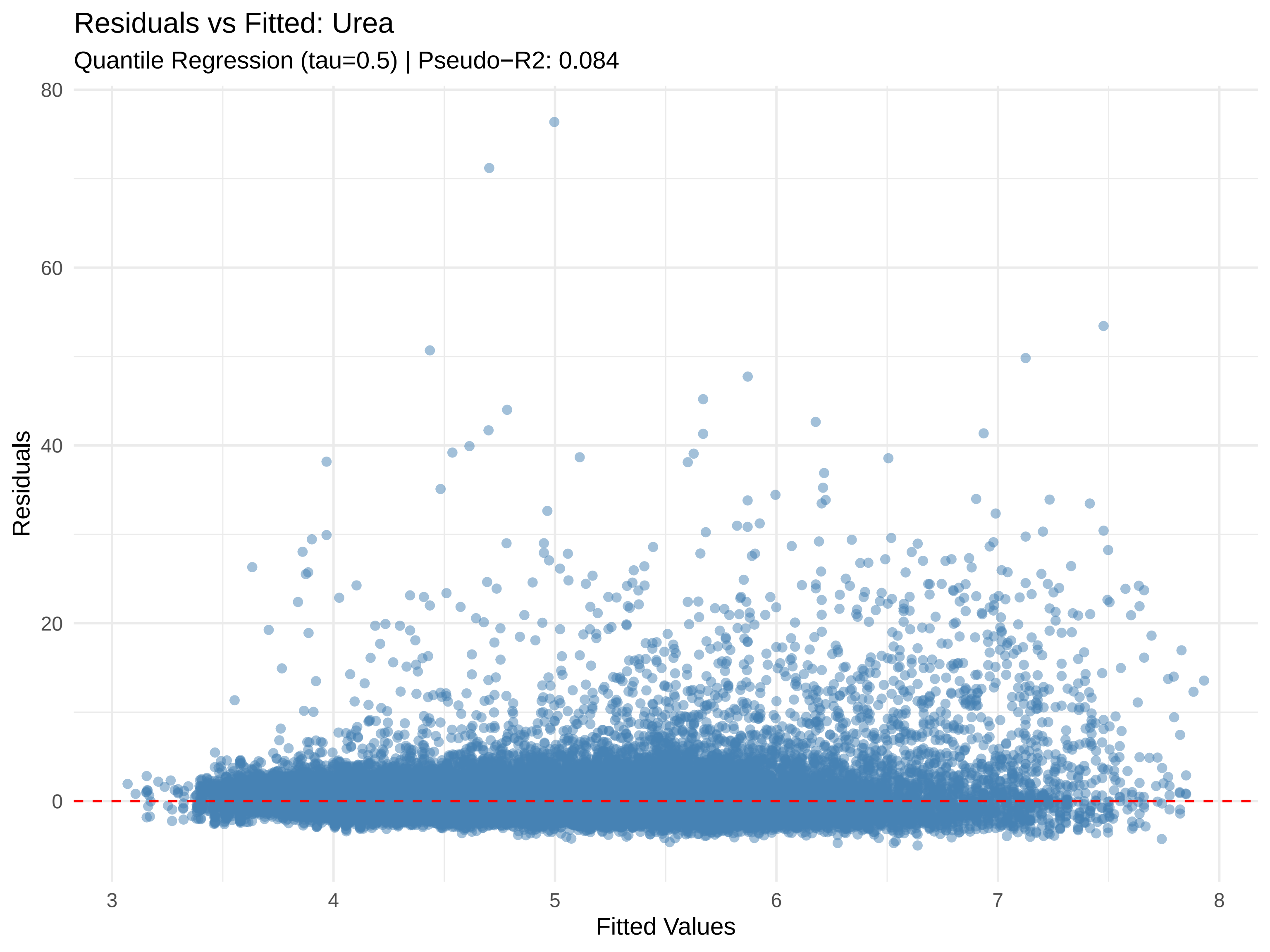
Residuals vs Fitted: Urea
Quantile Regression (tau=0.5) | Pseudo−R2: 0.084
Residuals
80
60
40
20
0
3
4
5
6
7
8
Fitted Values

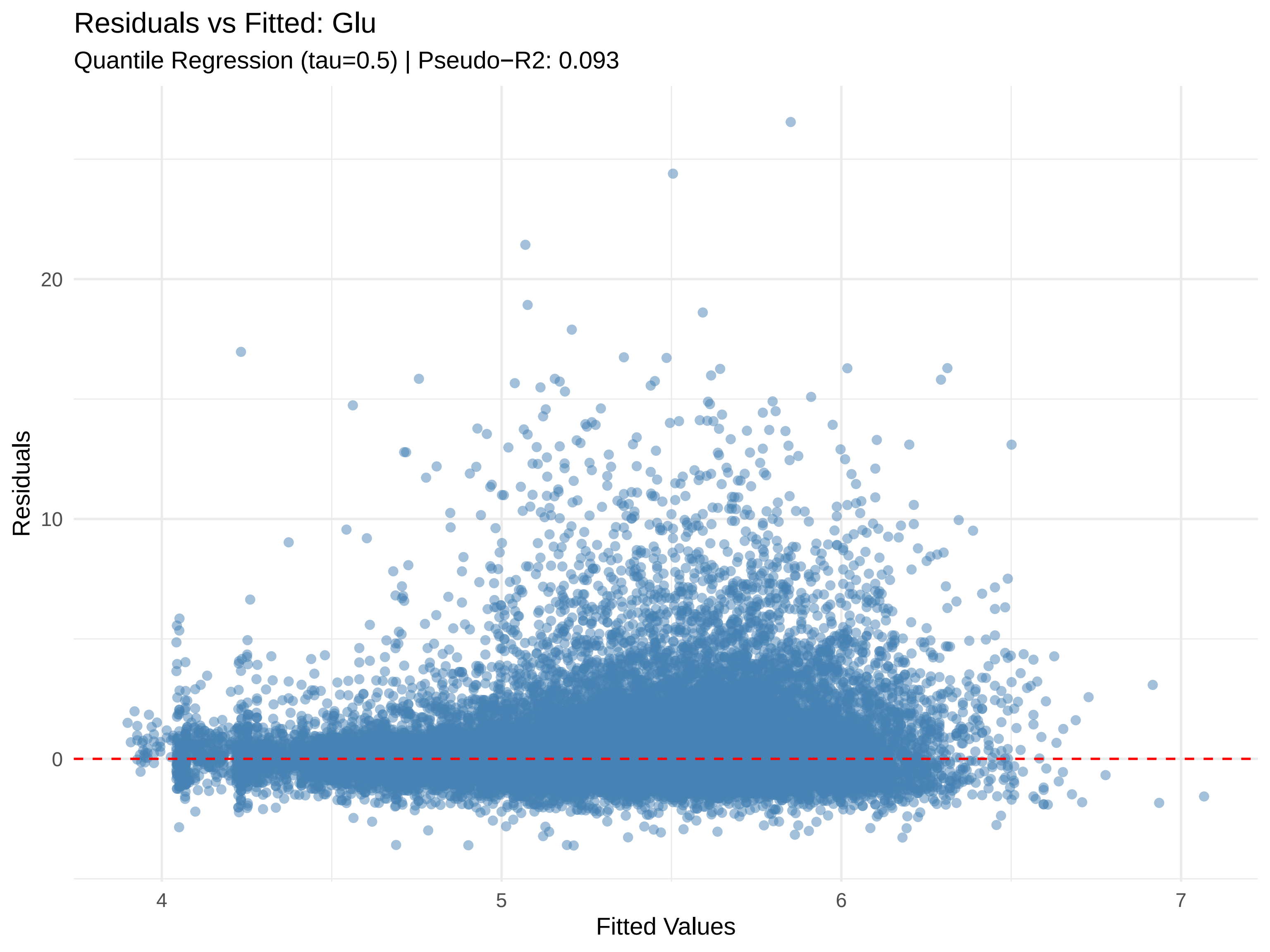

Residuals vs Fitted: Glu
Quantile Regression (tau=0.5) | Pseudo−R2: 0.093
Residuals
20
10
0
4
5
6
7
Fitted Values

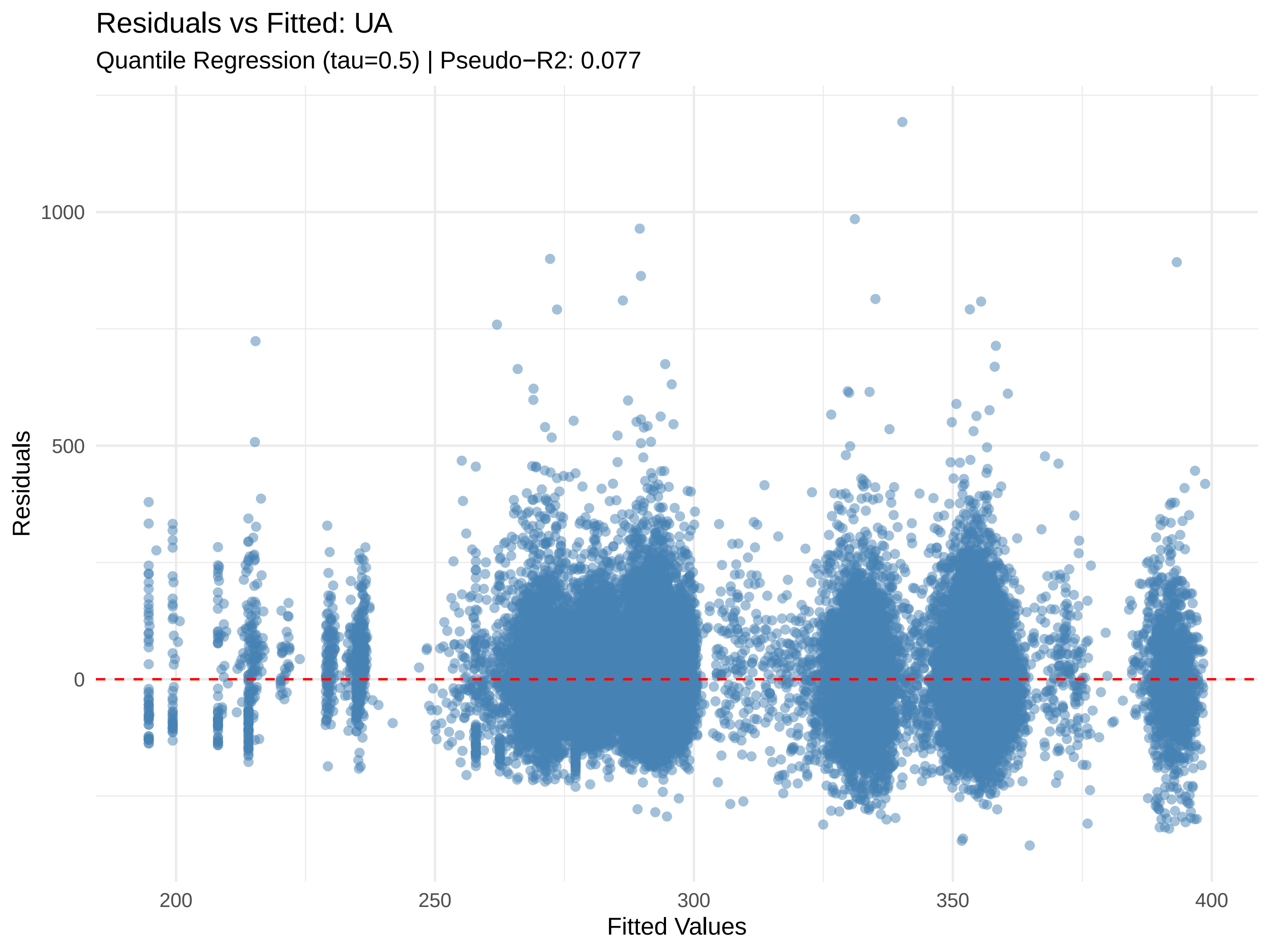

Residuals vs Fitted: UA
Quantile Regression (tau=0.5) | Pseudo−R2: 0.077
Residuals
1000
500
0
Fitted Values
200
250
300
350
400

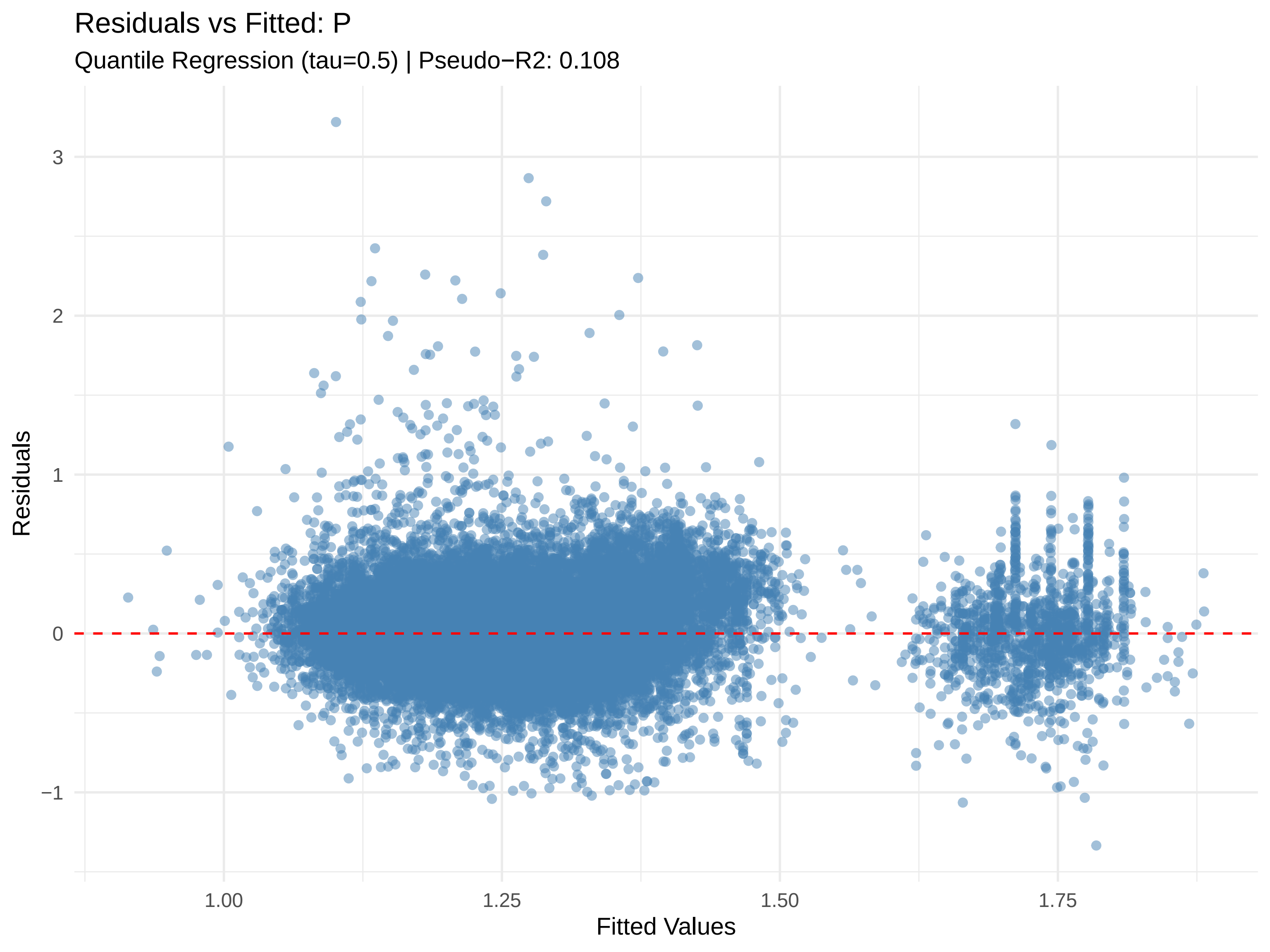
Residuals vs Fitted: P
Quantile Regression (tau=0.5) | Pseudo−R2: 0.108
Residuals
3
2
1
0
−1
1.00
1.25
1.50
1.75
Fitted Values

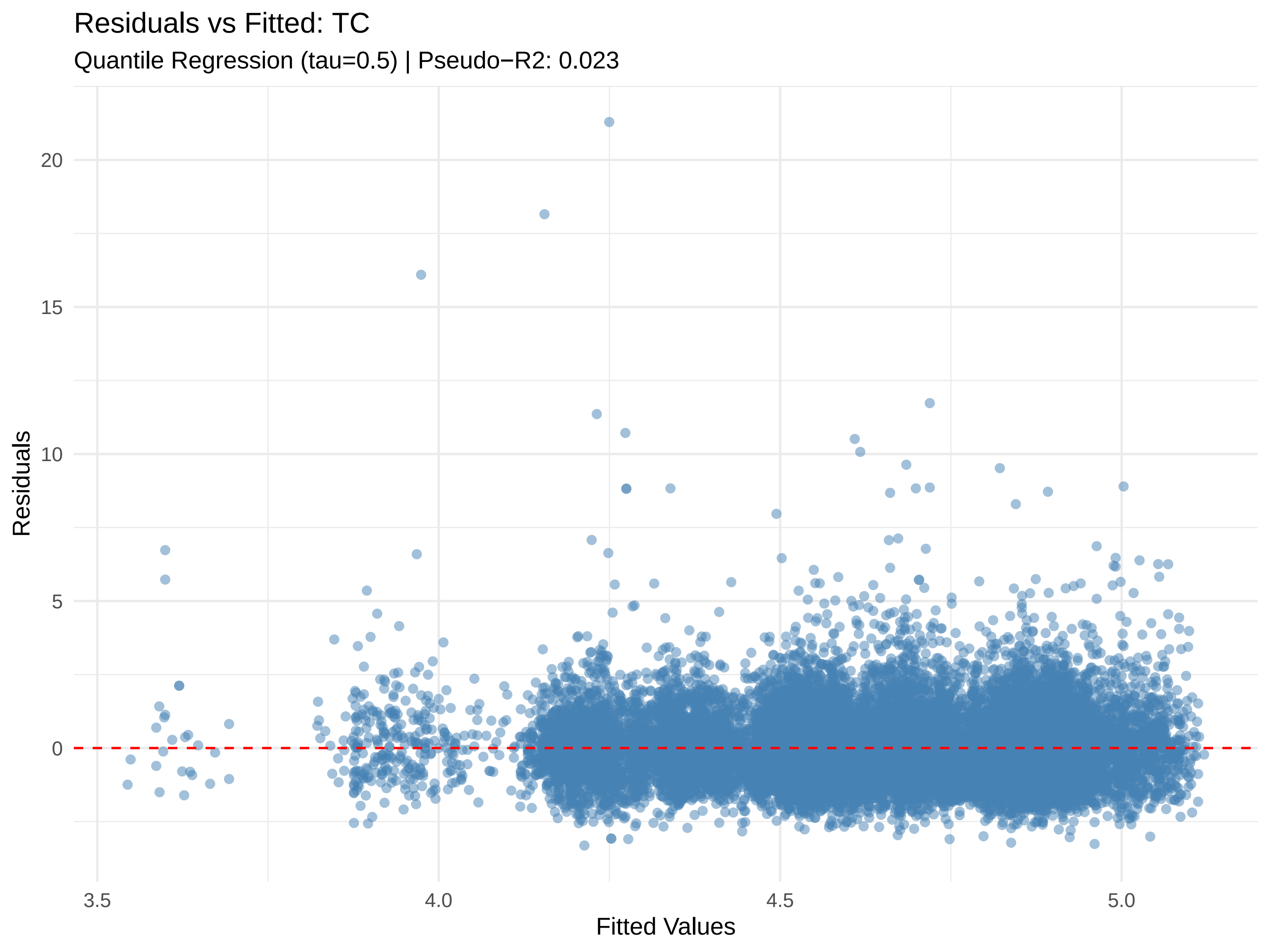

Residuals vs Fitted: TC
Quantile Regression (tau=0.5) | Pseudo−R2: 0.023
Residuals
Fitted Values
20
15
10
5
0
3.5
4.0
4.5
5.0

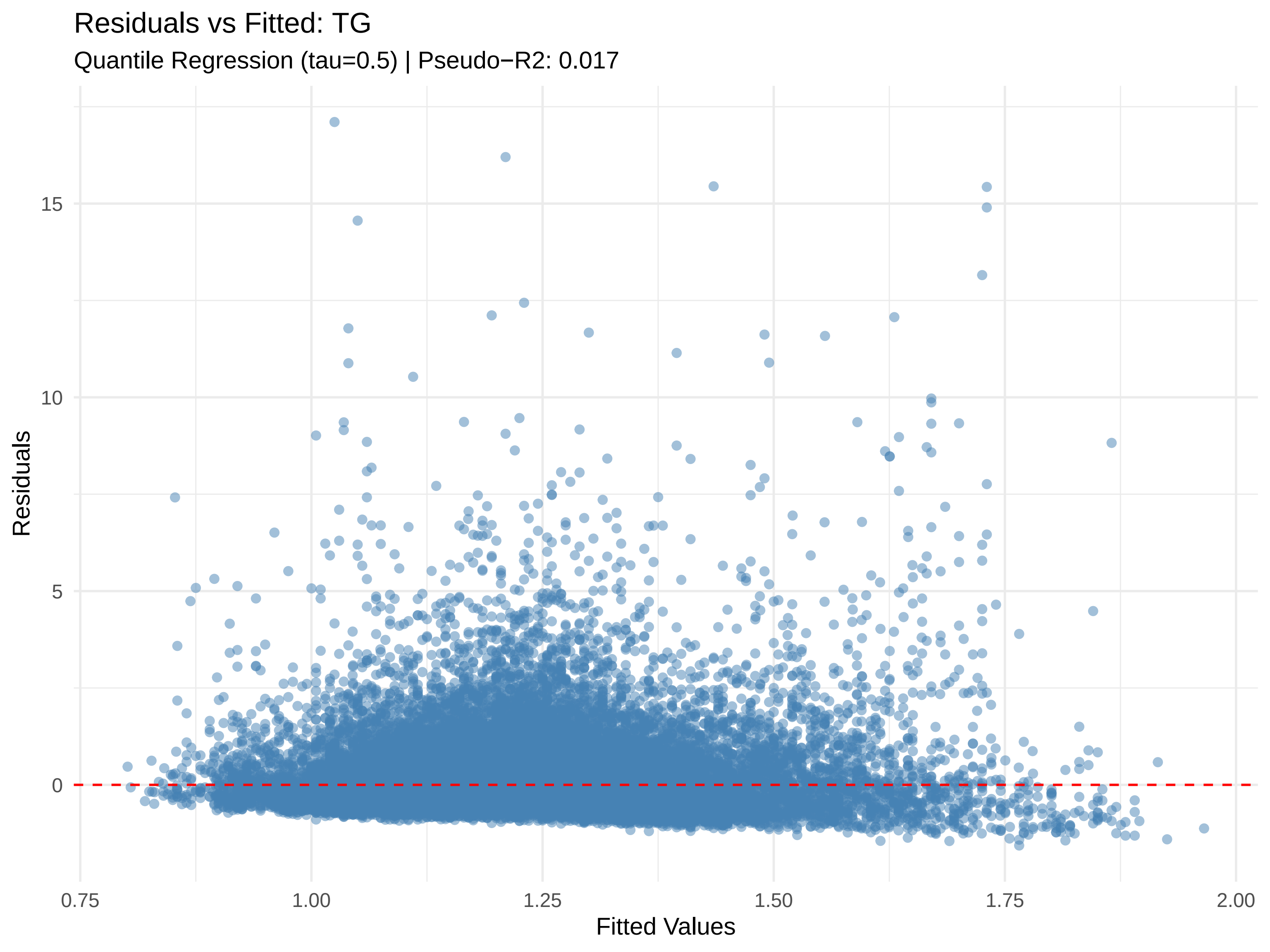
Residuals vs Fitted: TG
Quantile Regression (tau=0.5) | Pseudo−R2: 0.017
Residuals
15
10
5
0
0.75
1.00
1.25
1.50
1.75
2.00
Fitted Values

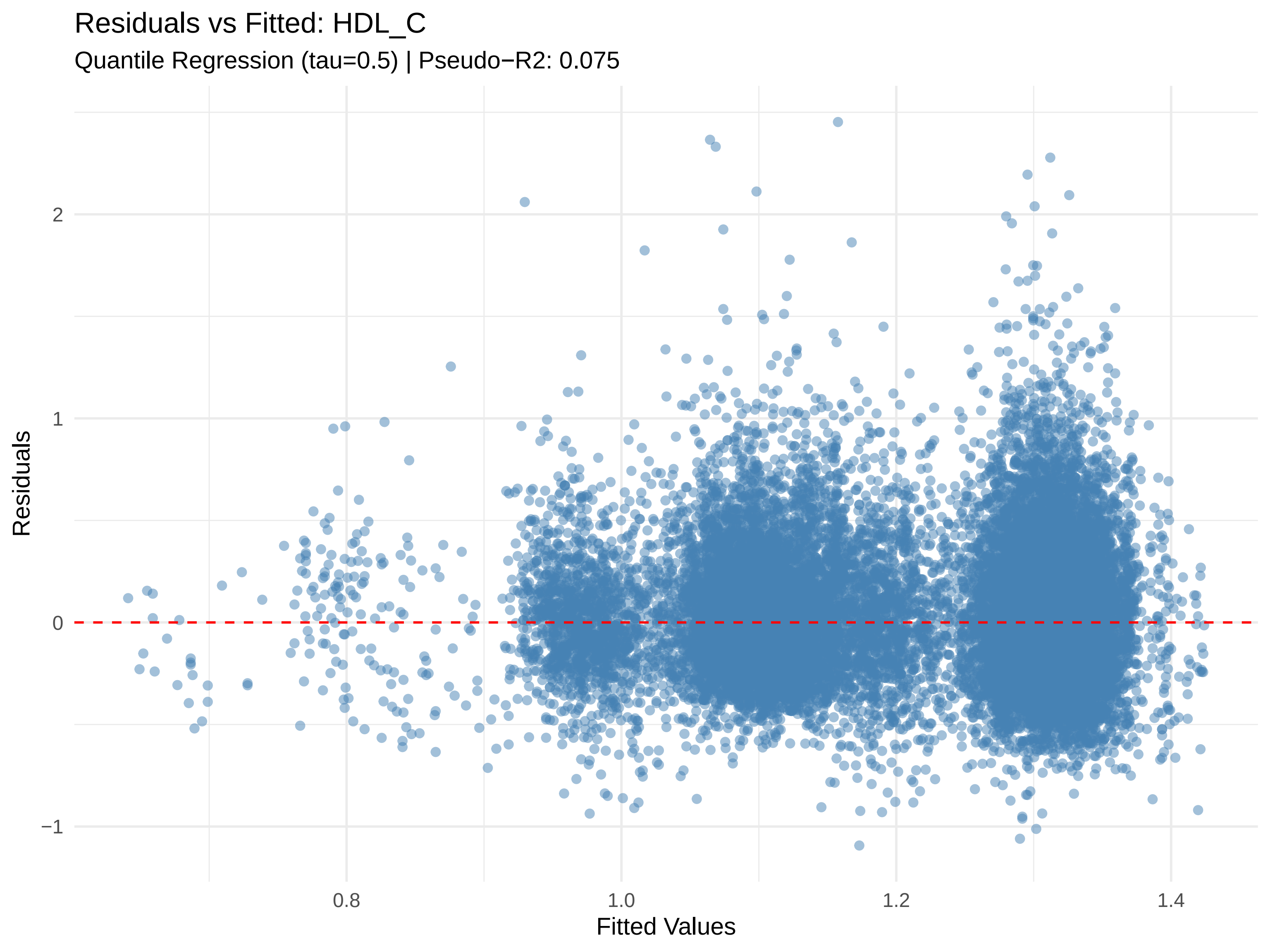

Residuals vs Fitted: HDL_C
Quantile Regression (tau=0.5) | Pseudo−R2: 0.075
Residuals
2
1
0
−1
0.8
1.0
1.2
1.4
Fitted Values

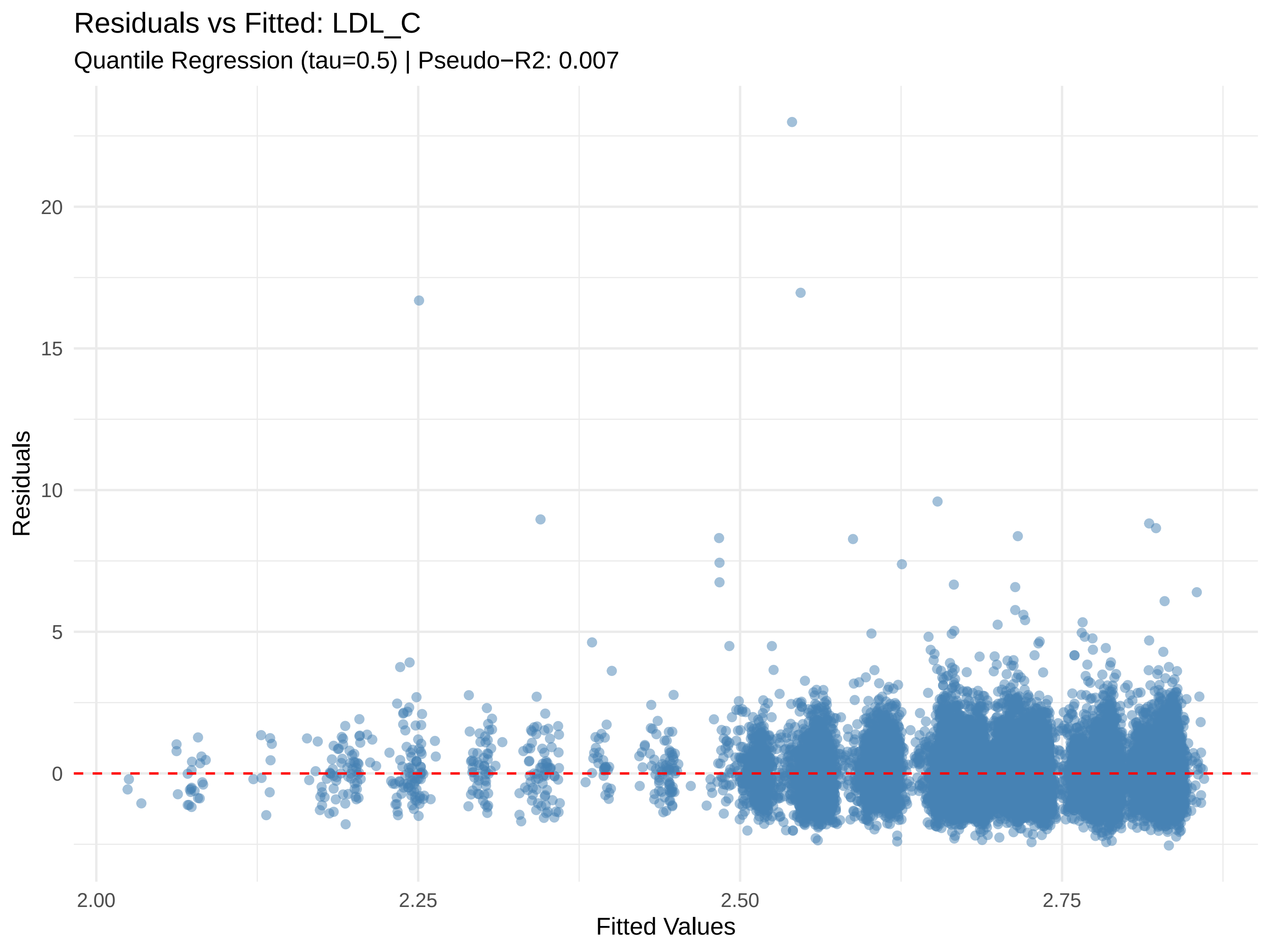
Residuals vs Fitted: LDL_C
Quantile Regression (tau=0.5) | Pseudo−R2: 0.007
Residuals
Fitted Values
20
15
10
5
0
2.00
2.25
2.50
2.75

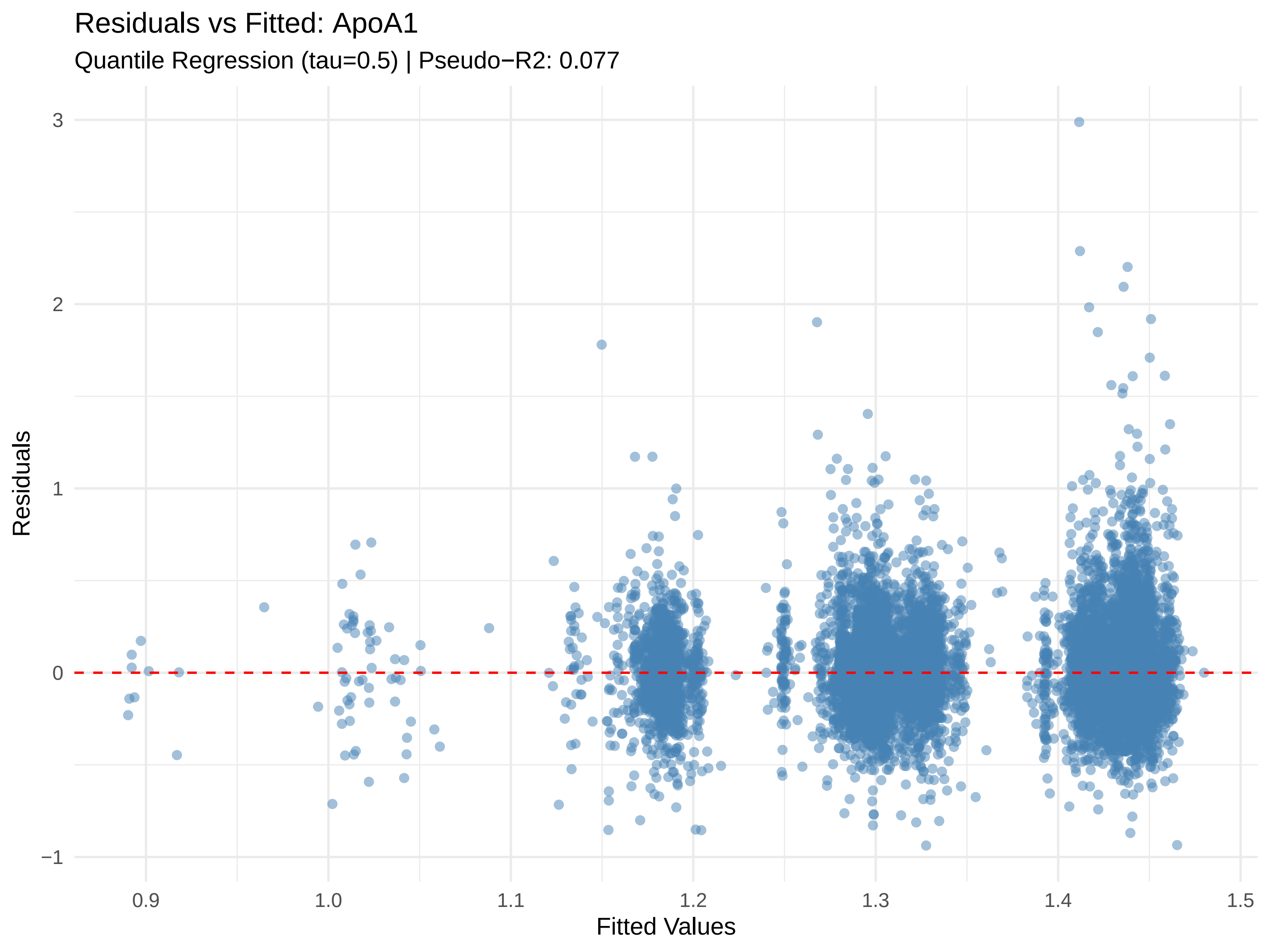
Residuals vs Fitted: ApoA1
Quantile Regression (tau=0.5) | Pseudo−R2: 0.077
Residuals
3
2
1
0
−1
0.9
1.0
1.1
1.2
1.3
1.4
1.5
Fitted Values

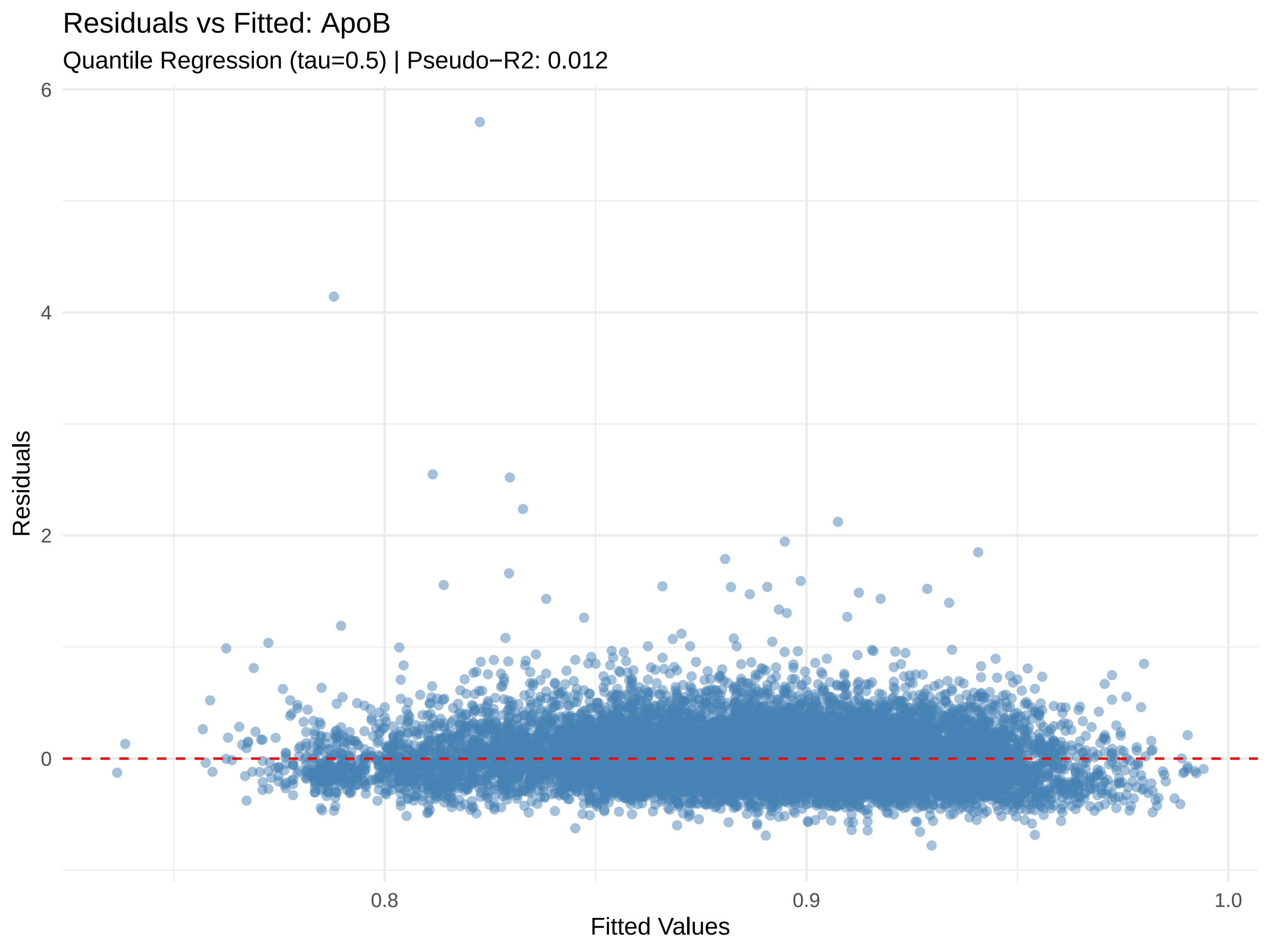

Residuals vs Fitted: ApoB
Quantile Regression (tau=0.5) | Pseudo−R2: 0.012
6
4
2
0
Residuals
0.8
0.9
1.0
Fitted Values

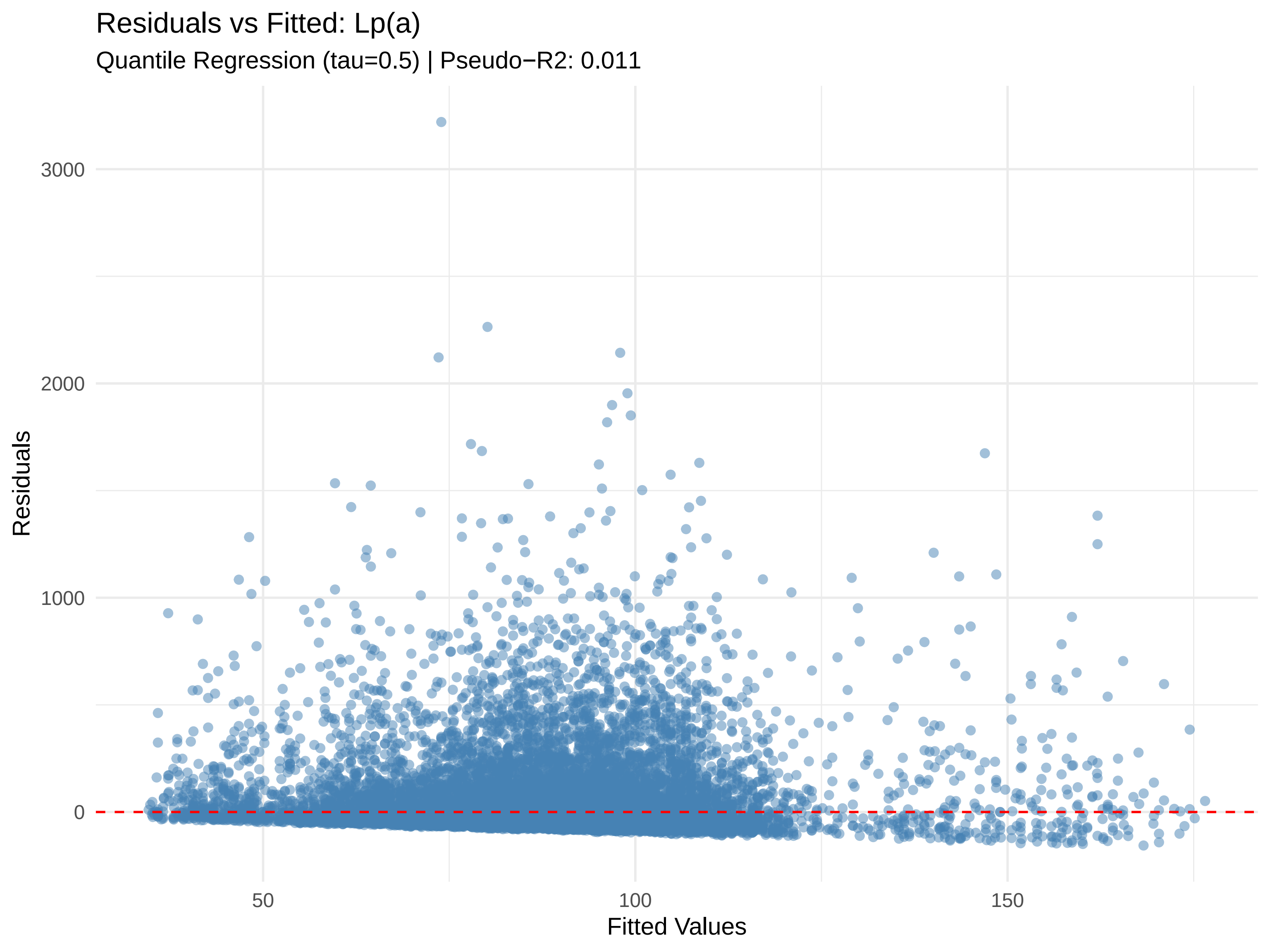

Residuals vs Fitted: Lp(a)
Quantile Regression (tau=0.5) | Pseudo−R2: 0.011
Residuals
3000
2000
1000
0
50
100
150
Fitted Values

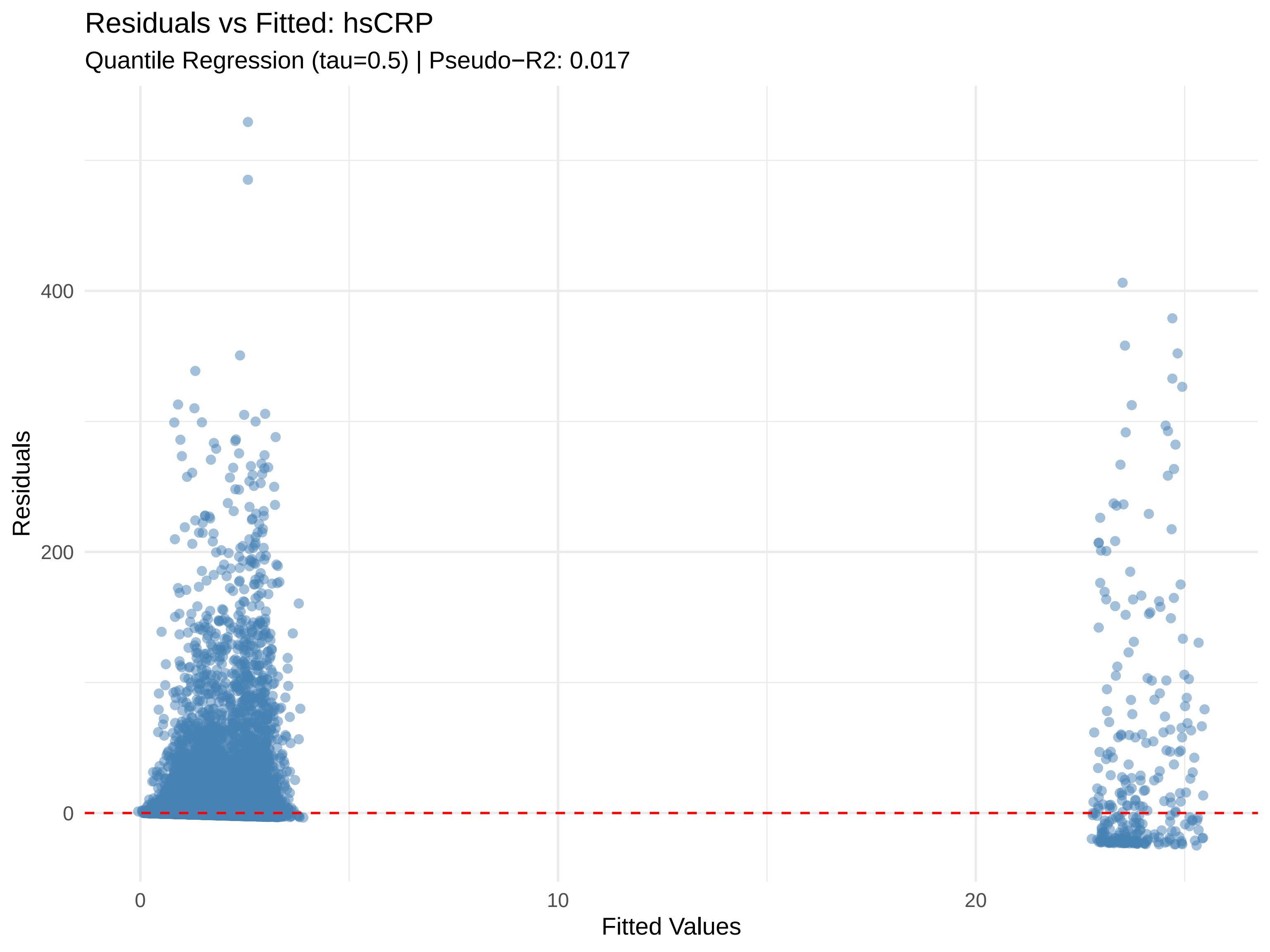

Residuals vs Fitted: hsCRP
Quantile Regression (tau=0.5) | Pseudo−R2: 0.017
400
200
0
Residuals
0
10
20
Fitted Values

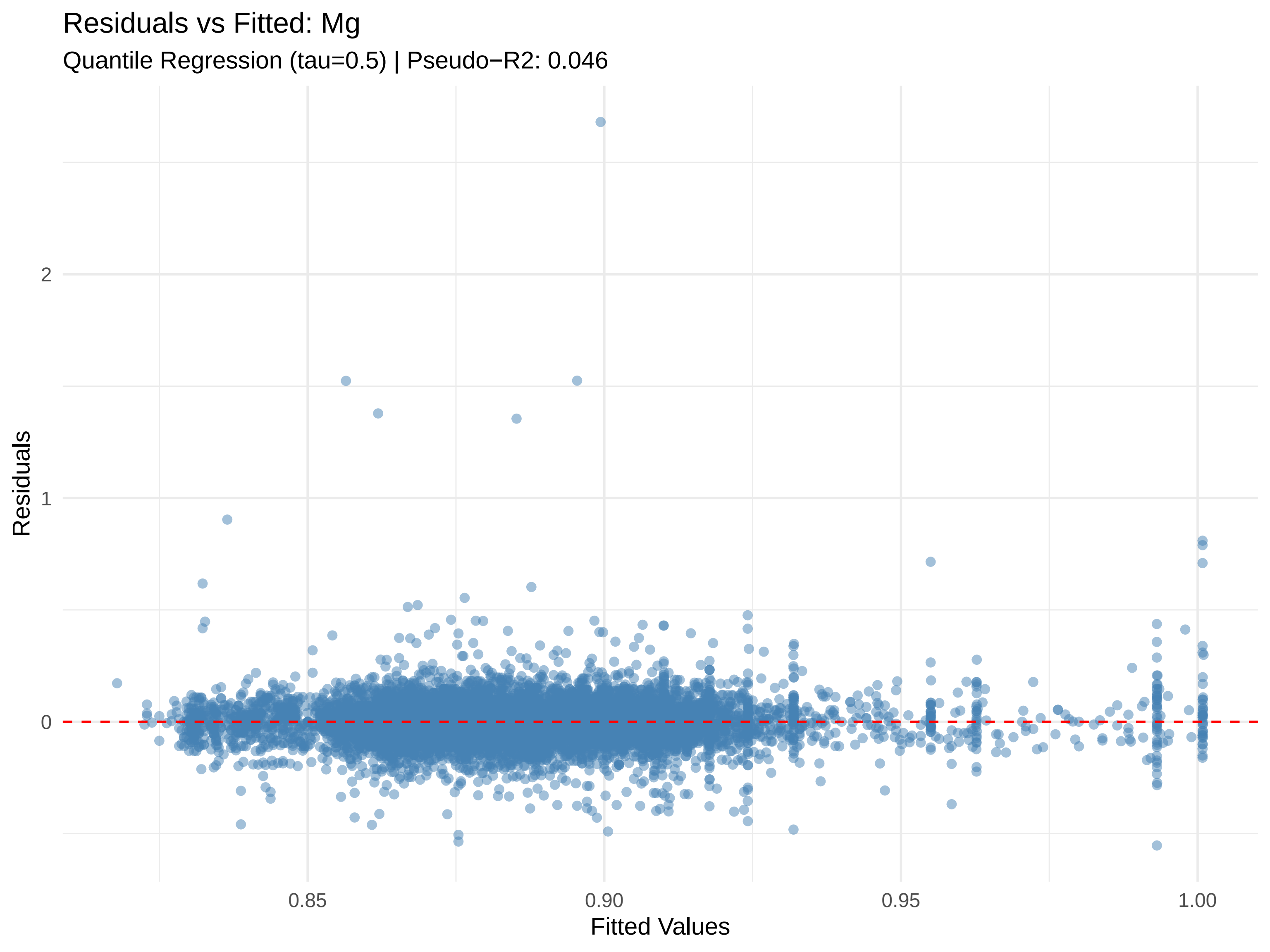
Residuals vs Fitted: Mg
Quantile Regression (tau=0.5) | Pseudo−R2: 0.046
Residuals
2
1
0
0.85
0.90
0.95
1.00
Fitted Values

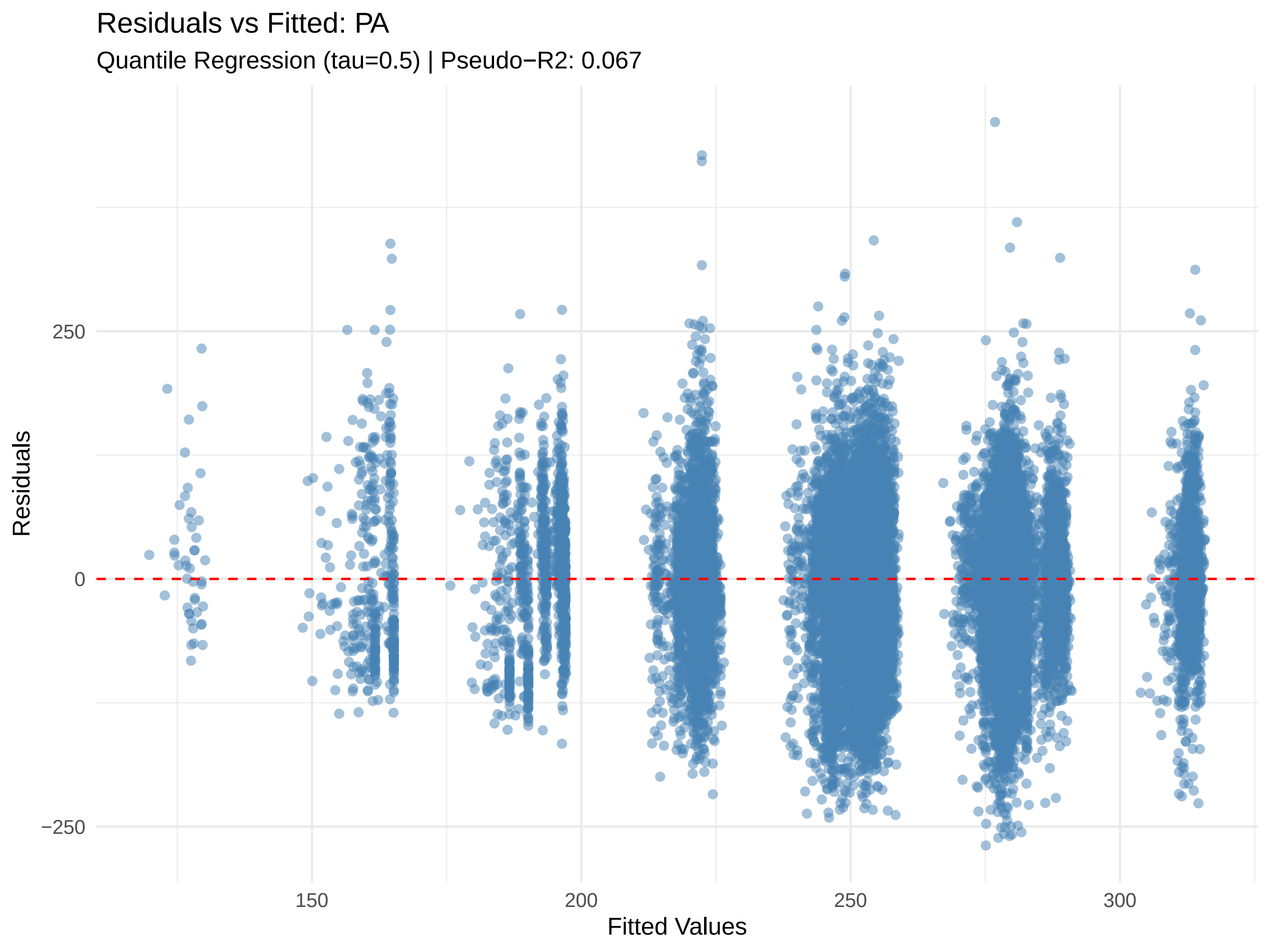

Residuals vs Fitted: PA
Quantile Regression (tau=0.5) | Pseudo−R2: 0.067
Residuals
250
0
−250
150
200
250
300
Fitted Values

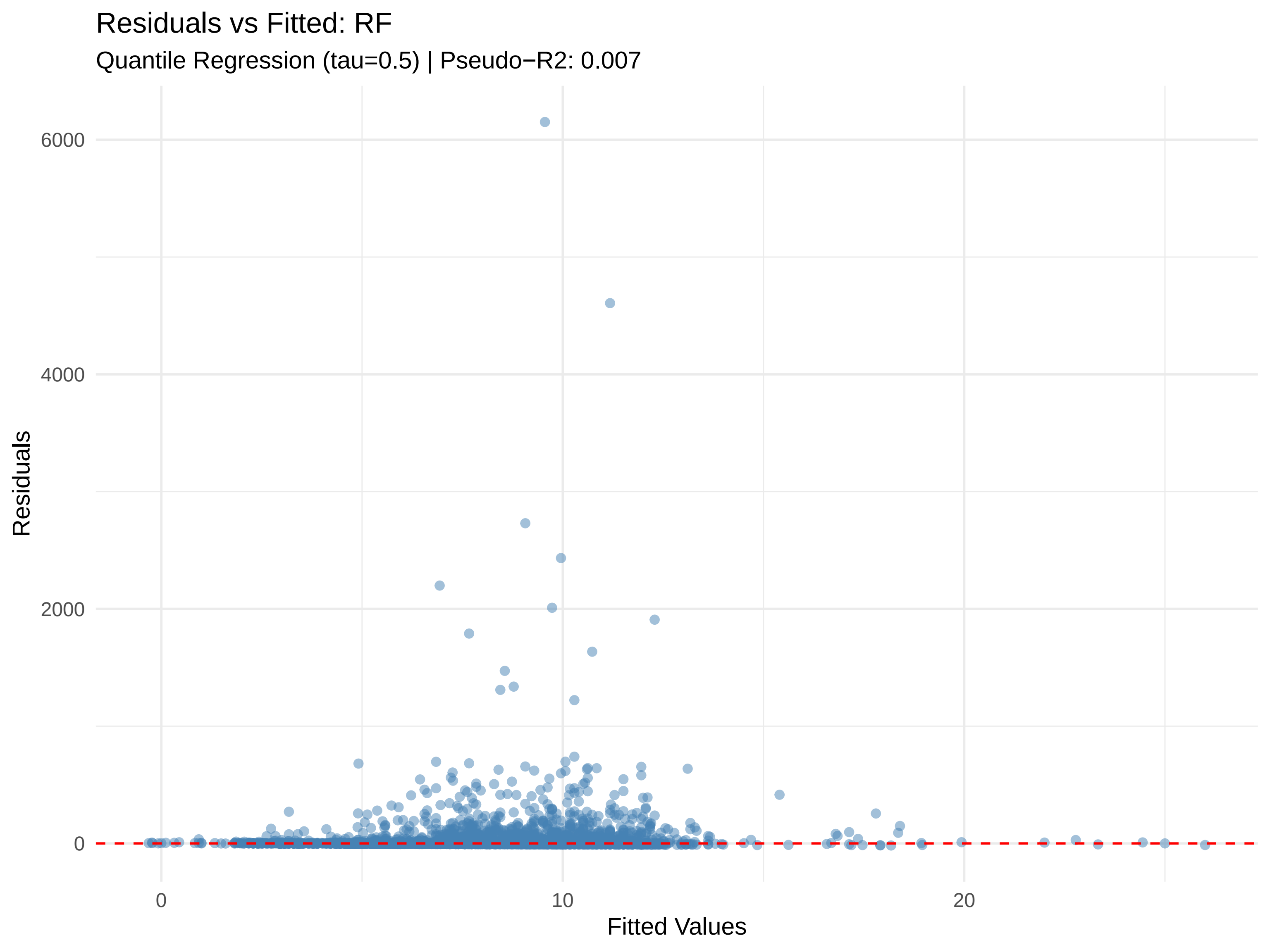

Residuals vs Fitted: RF
Quantile Regression (tau=0.5) | Pseudo−R2: 0.007
Residuals
6000
4000
2000
0
Fitted Values
0
10
20

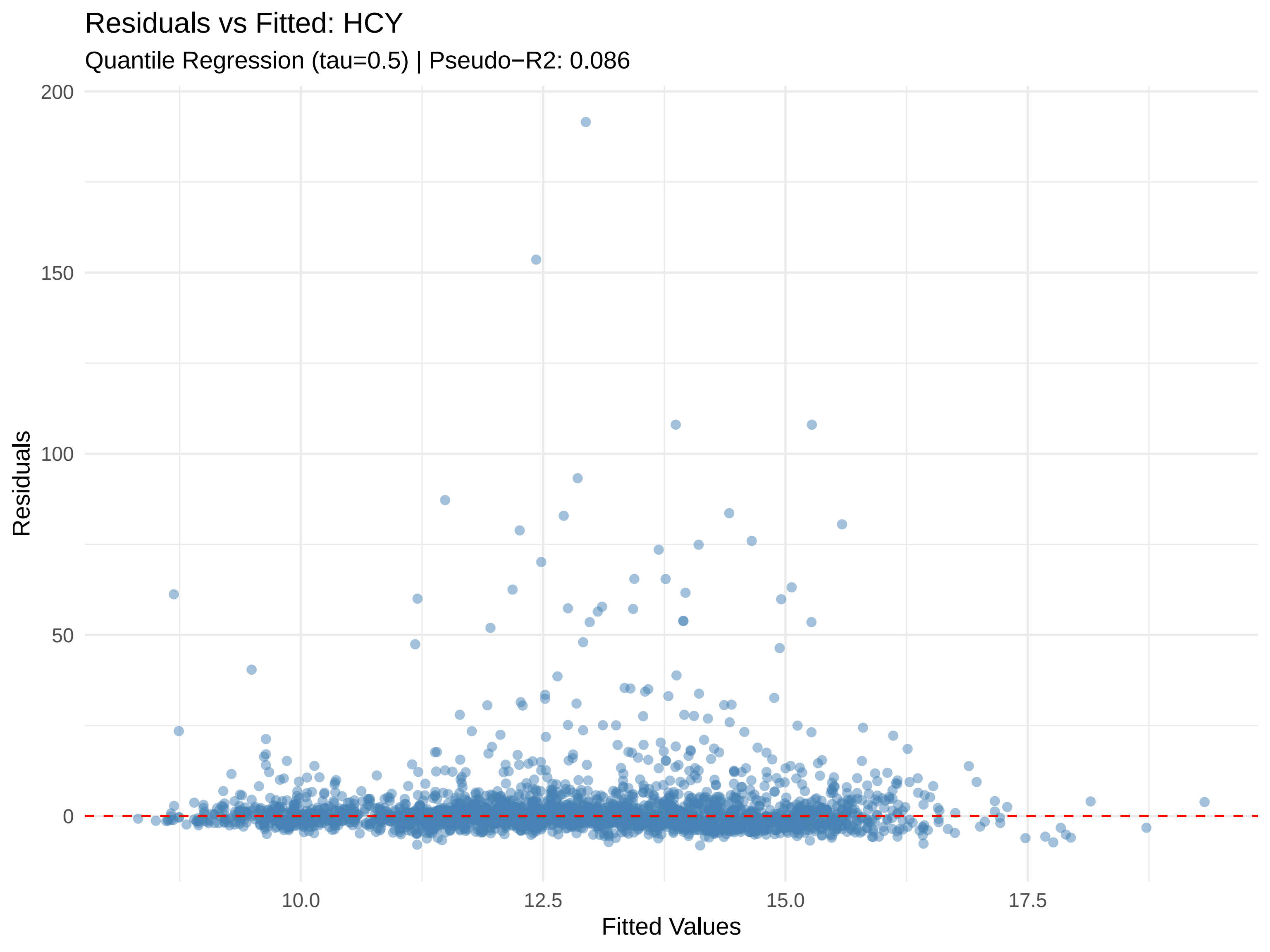
Residuals vs Fitted: HCY
Quantile Regression (tau=0.5) | Pseudo−R2: 0.086
Residuals
200
150
100
50
0
10.0
12.5
15.0
17.5
Fitted Values

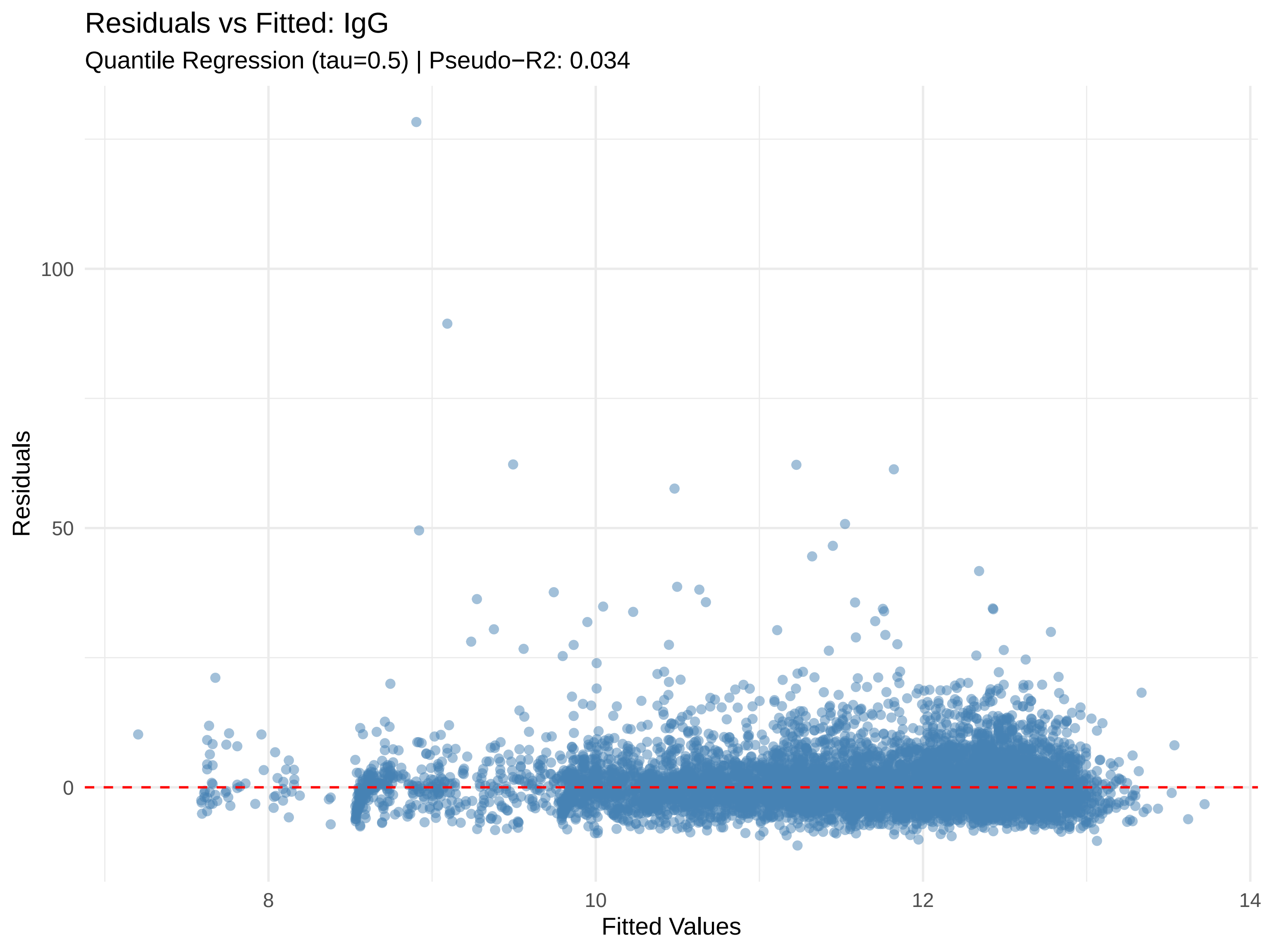
Residuals vs Fitted: IgG
Quantile Regression (tau=0.5) | Pseudo−R2: 0.034
Residuals
100
50
0
8
10
12
14
Fitted Values

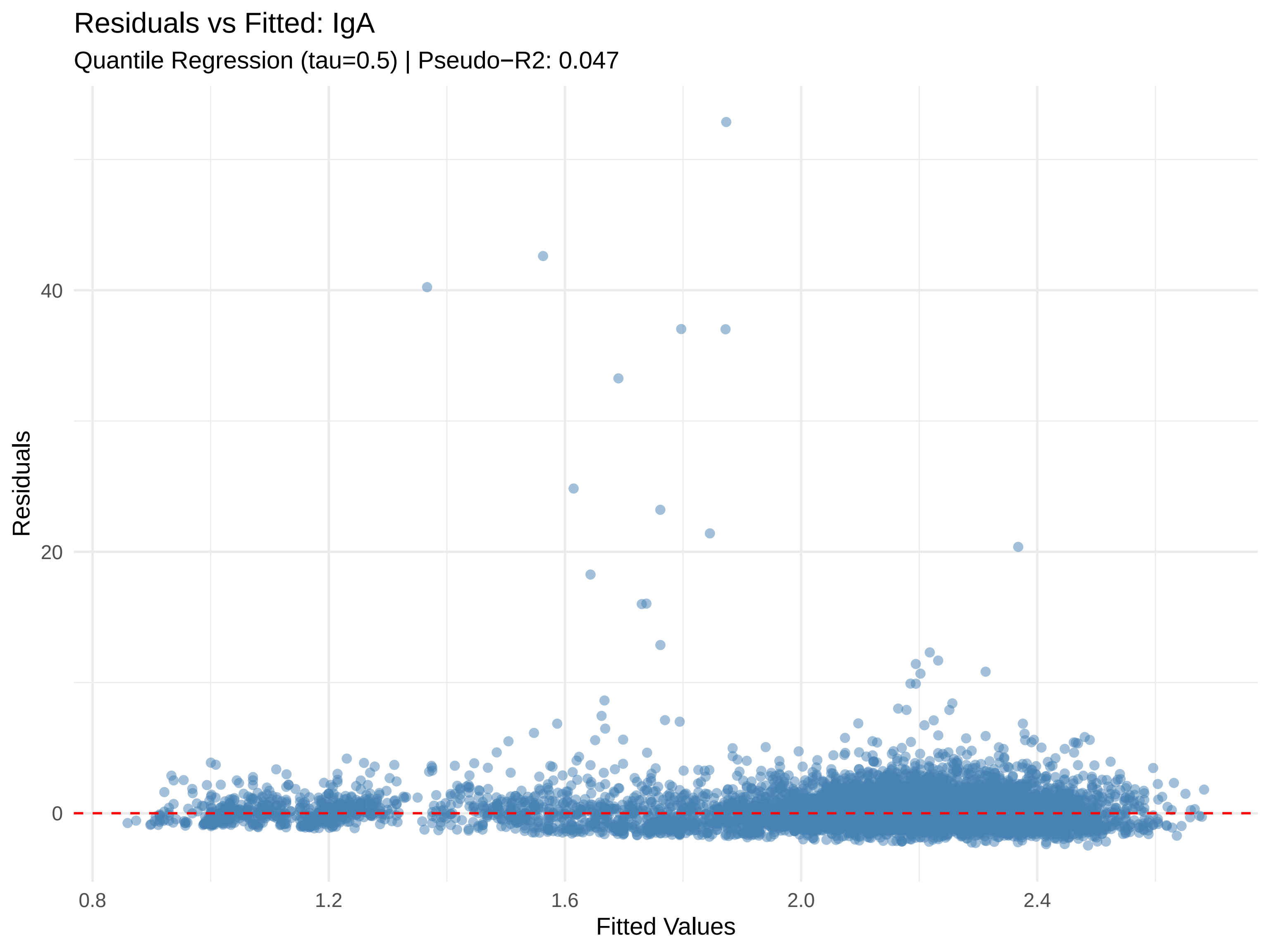
Residuals vs Fitted: IgA
Quantile Regression (tau=0.5) | Pseudo−R2: 0.047
Residuals
40
20
0
0.8
1.2
1.6
2.0
2.4
Fitted Values

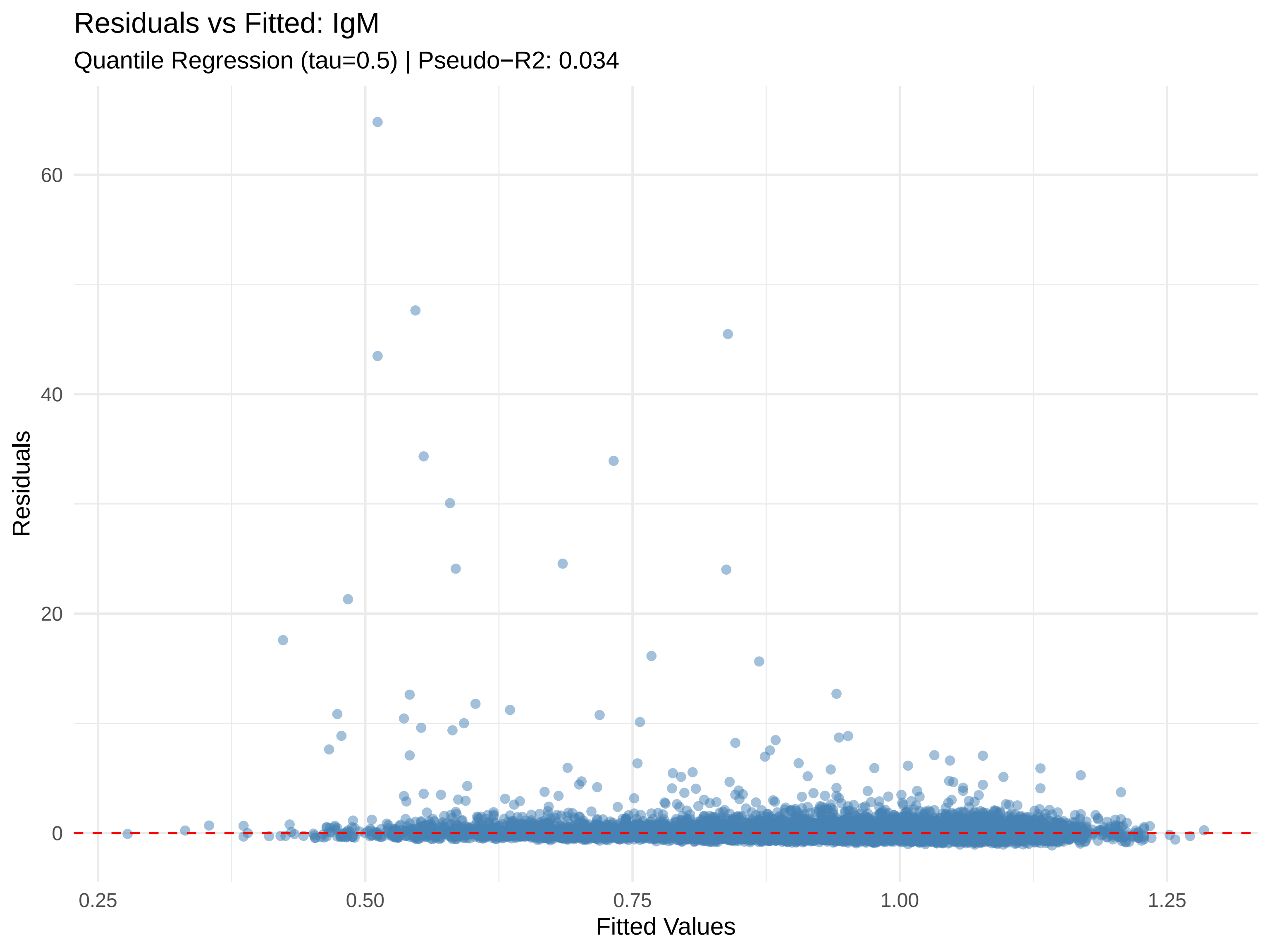

Residuals vs Fitted: IgM
Quantile Regression (tau=0.5) | Pseudo−R2: 0.034
Residuals
60
40
20
0
0.25
0.50
0.75
1.00
1.25
Fitted Values

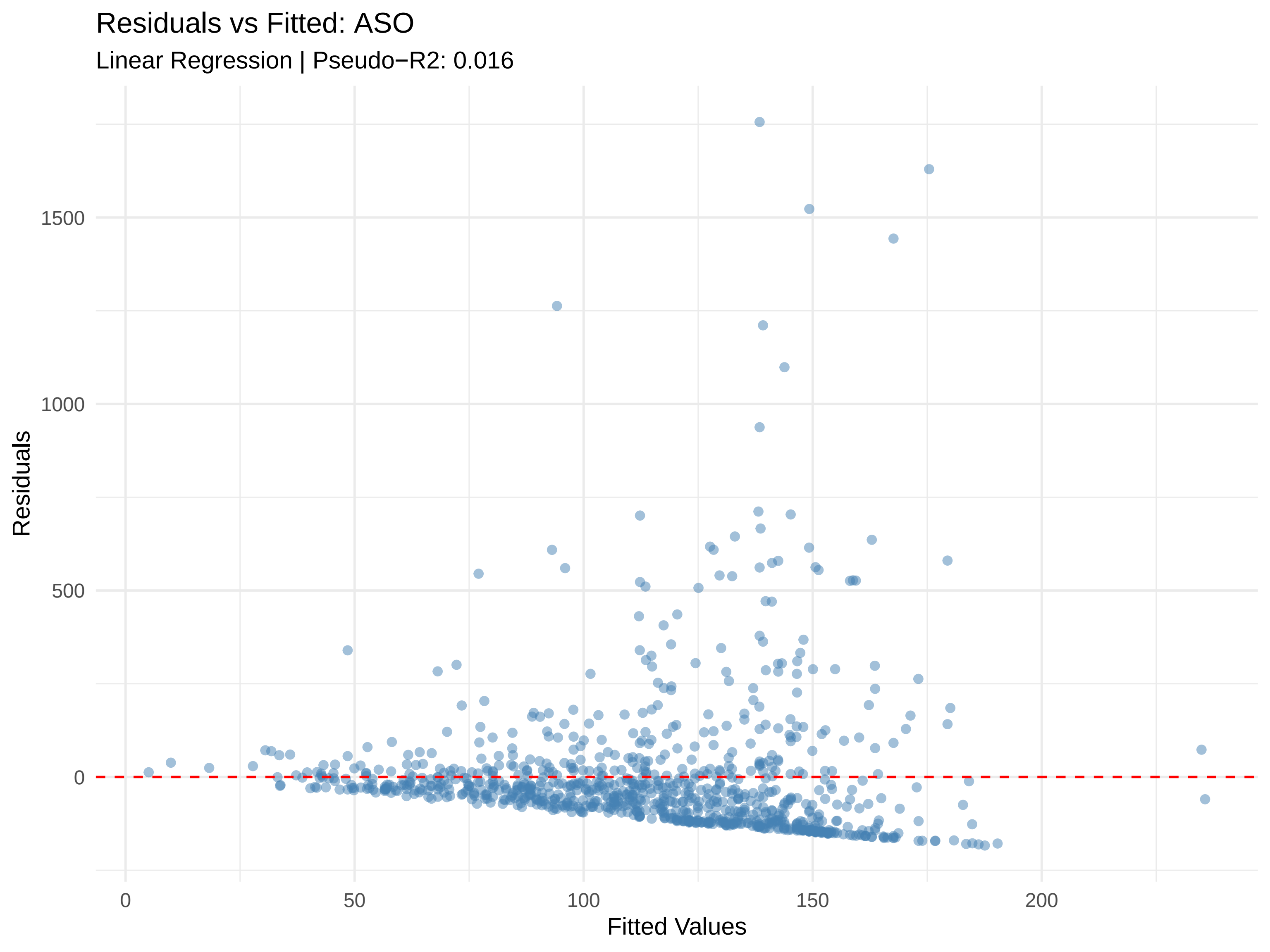

Residuals vs Fitted: ASO
Linear Regression | Pseudo−R2: 0.016
Residuals
1500
1000
500
0
0
50
100
150
200
Fitted Values

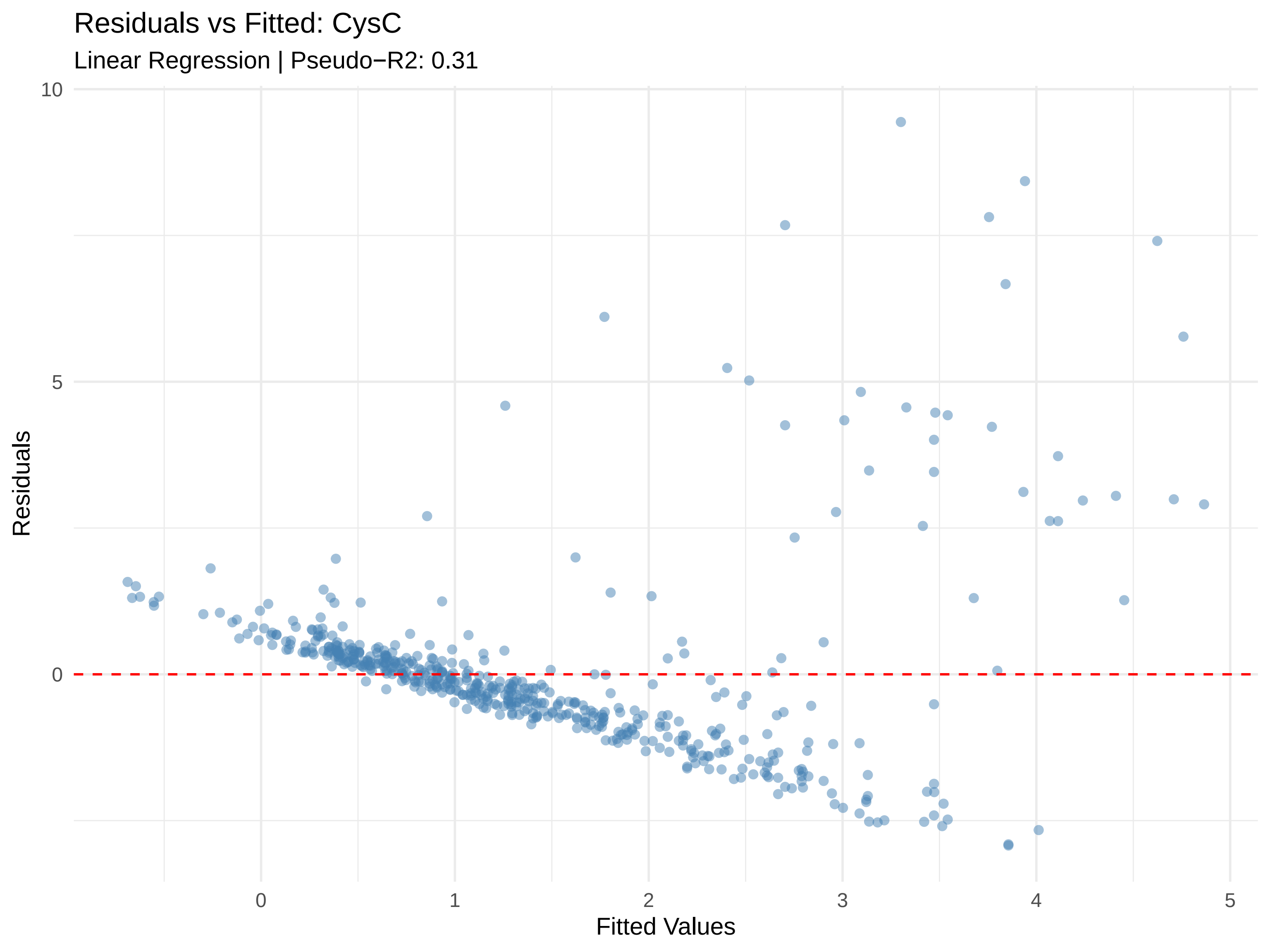

Residuals vs Fitted: CysC
Linear Regression | Pseudo−R2: 0.31
10
5
0
Residuals
0
1
2
3
4
5
Fitted Values

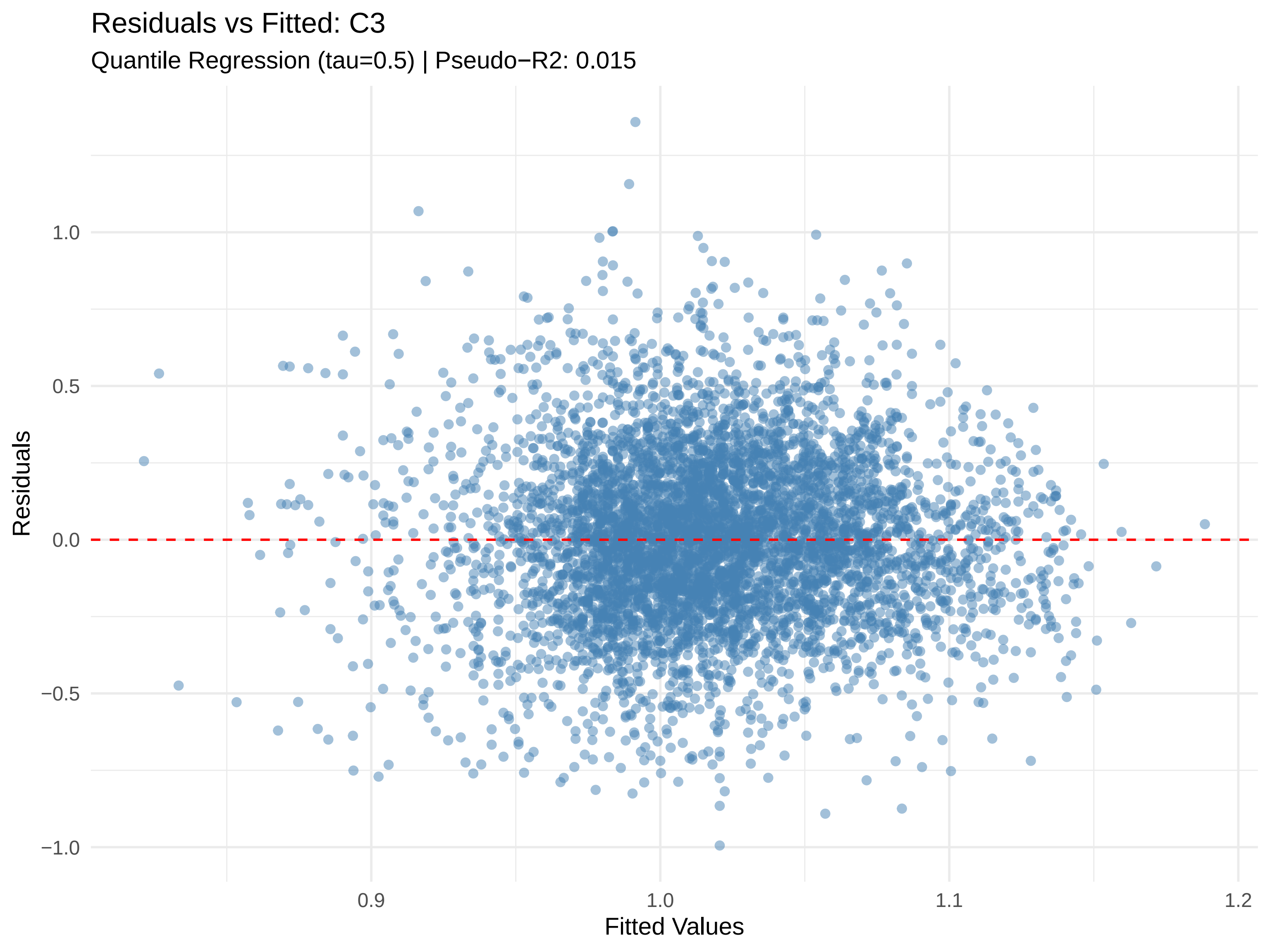
Residuals vs Fitted: C3
Quantile Regression (tau=0.5) | Pseudo−R2: 0.015
Residuals
1.0
0.5
0.0
−0.5
−1.0
0.9
1.0
1.1
1.2
Fitted Values

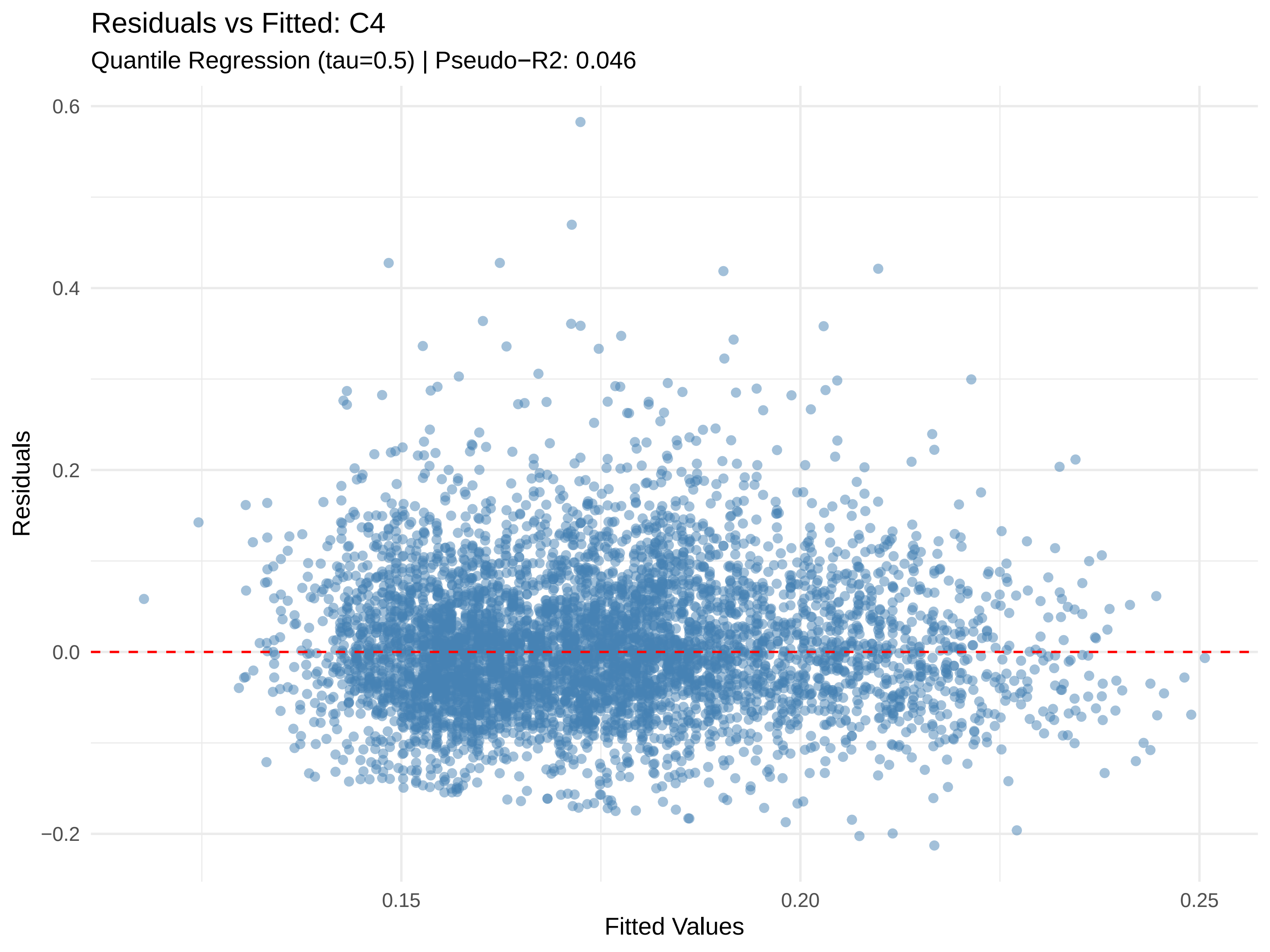

Residuals vs Fitted: C4
Quantile Regression (tau=0.5) | Pseudo−R2: 0.046
0.6
0.4
0.2
0.0
−0.2
Residuals
0.15
0.20
0.25
Fitted Values

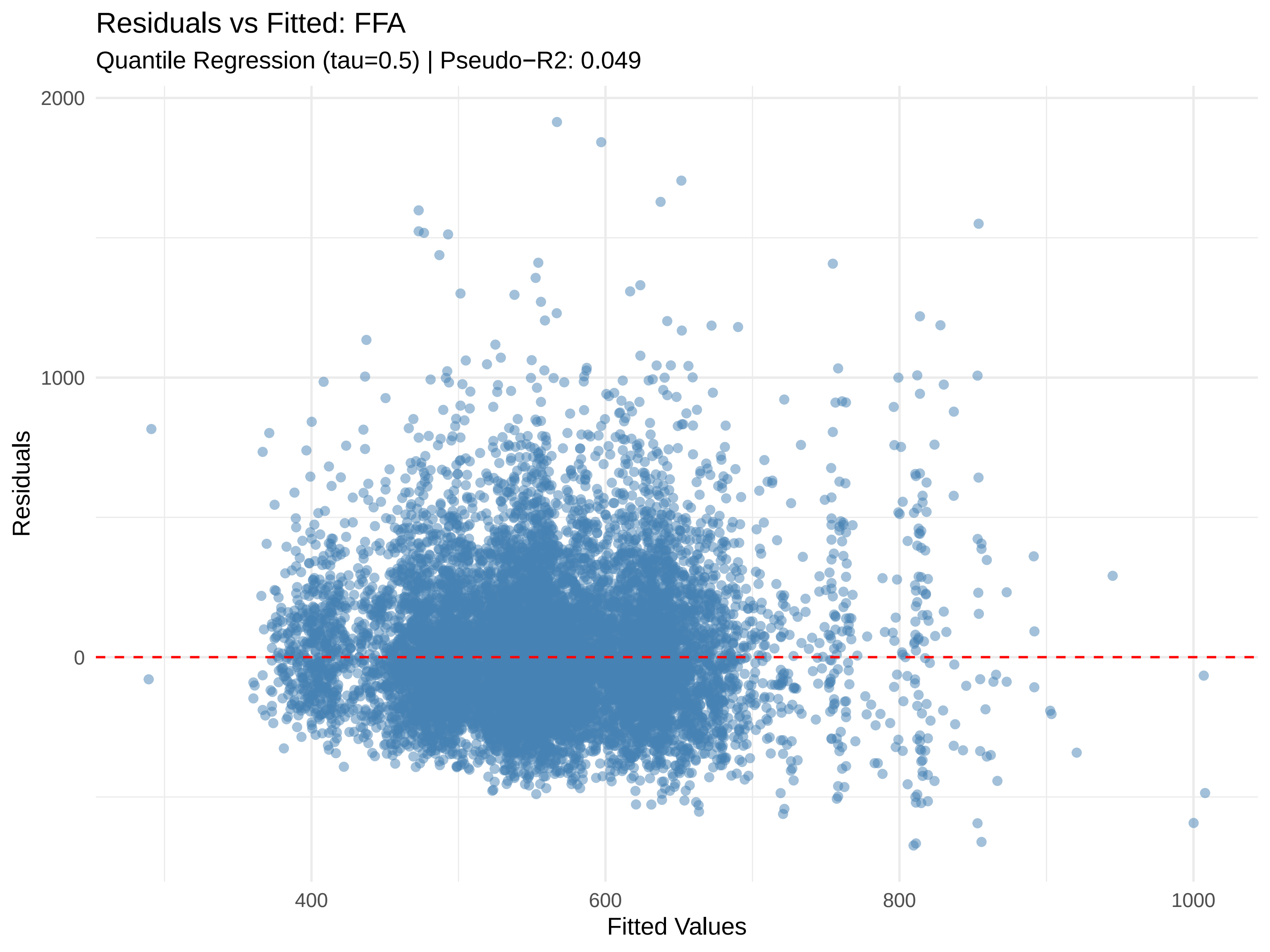

Residuals vs Fitted: FFA
Quantile Regression (tau=0.5) | Pseudo−R2: 0.049
2000
1000
0
Residuals
400
600
800
1000
Fitted Values

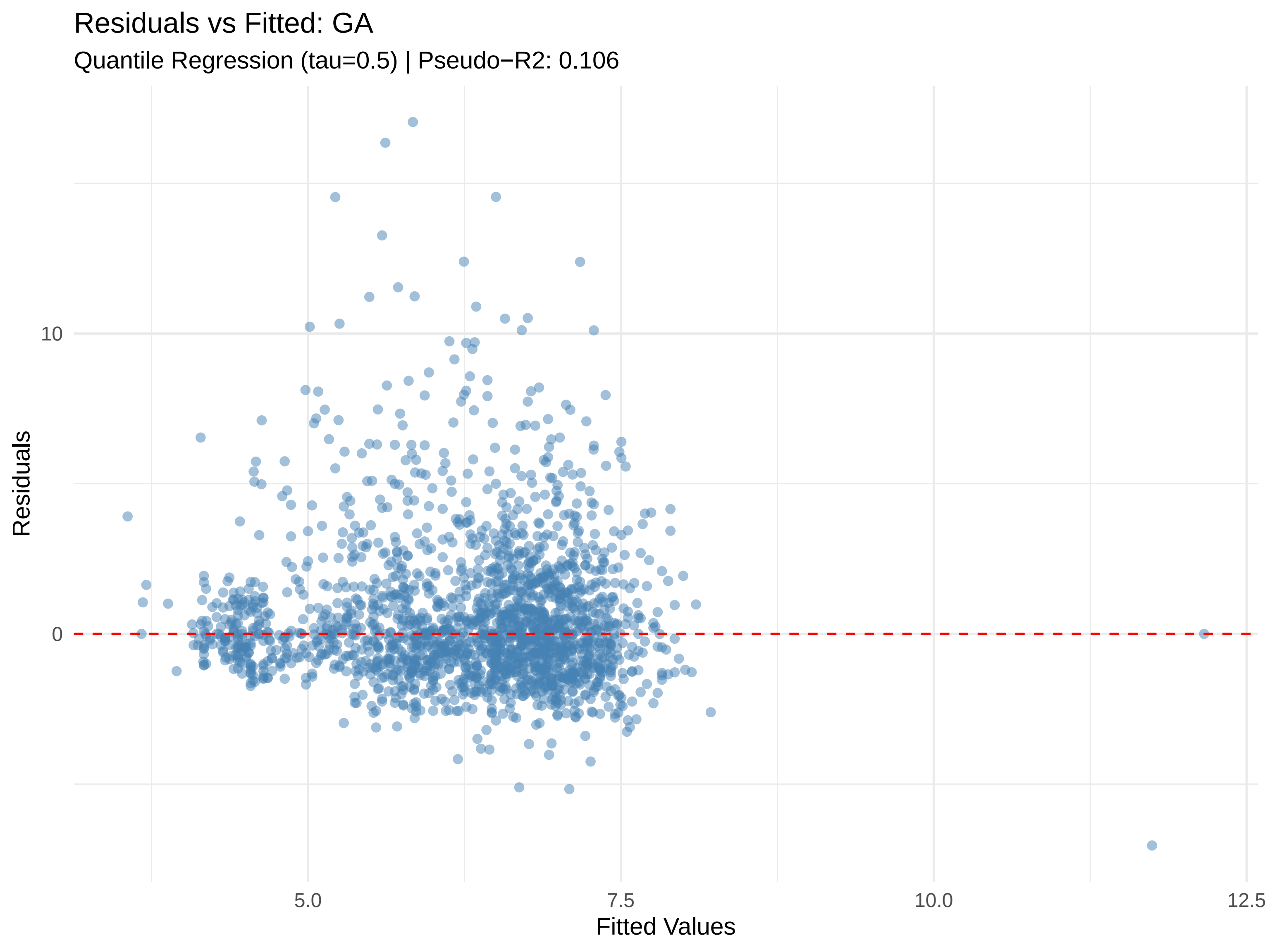

Residuals vs Fitted: GA
Quantile Regression (tau=0.5) | Pseudo−R2: 0.106
Residuals
10
0
5.0
7.5
10.0
12.5
Fitted Values

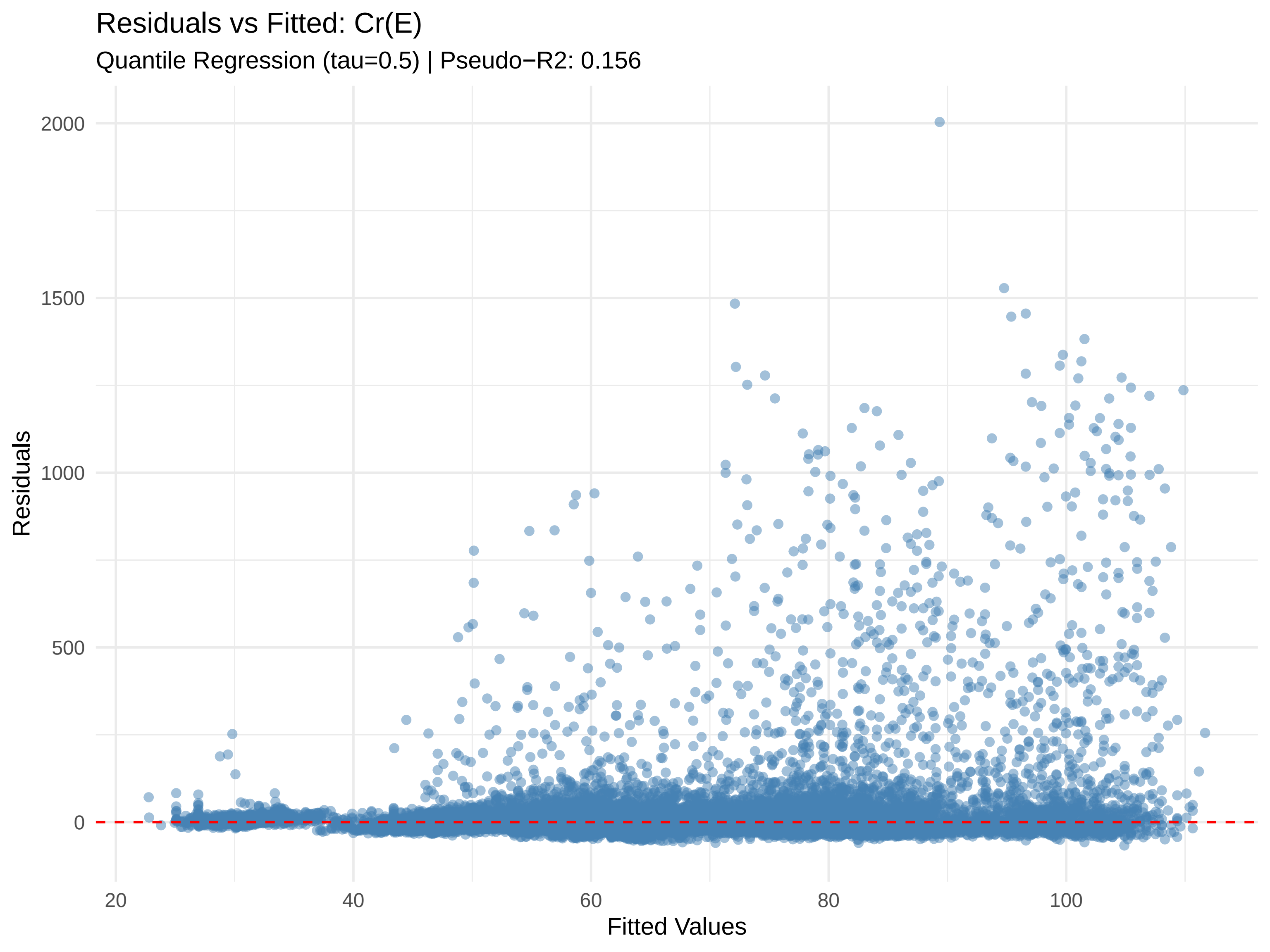

Residuals vs Fitted: Cr(E)
Quantile Regression (tau=0.5) | Pseudo−R2: 0.156
Residuals
2000
1500
1000
500
0
20
40
60
80
100
Fitted Values

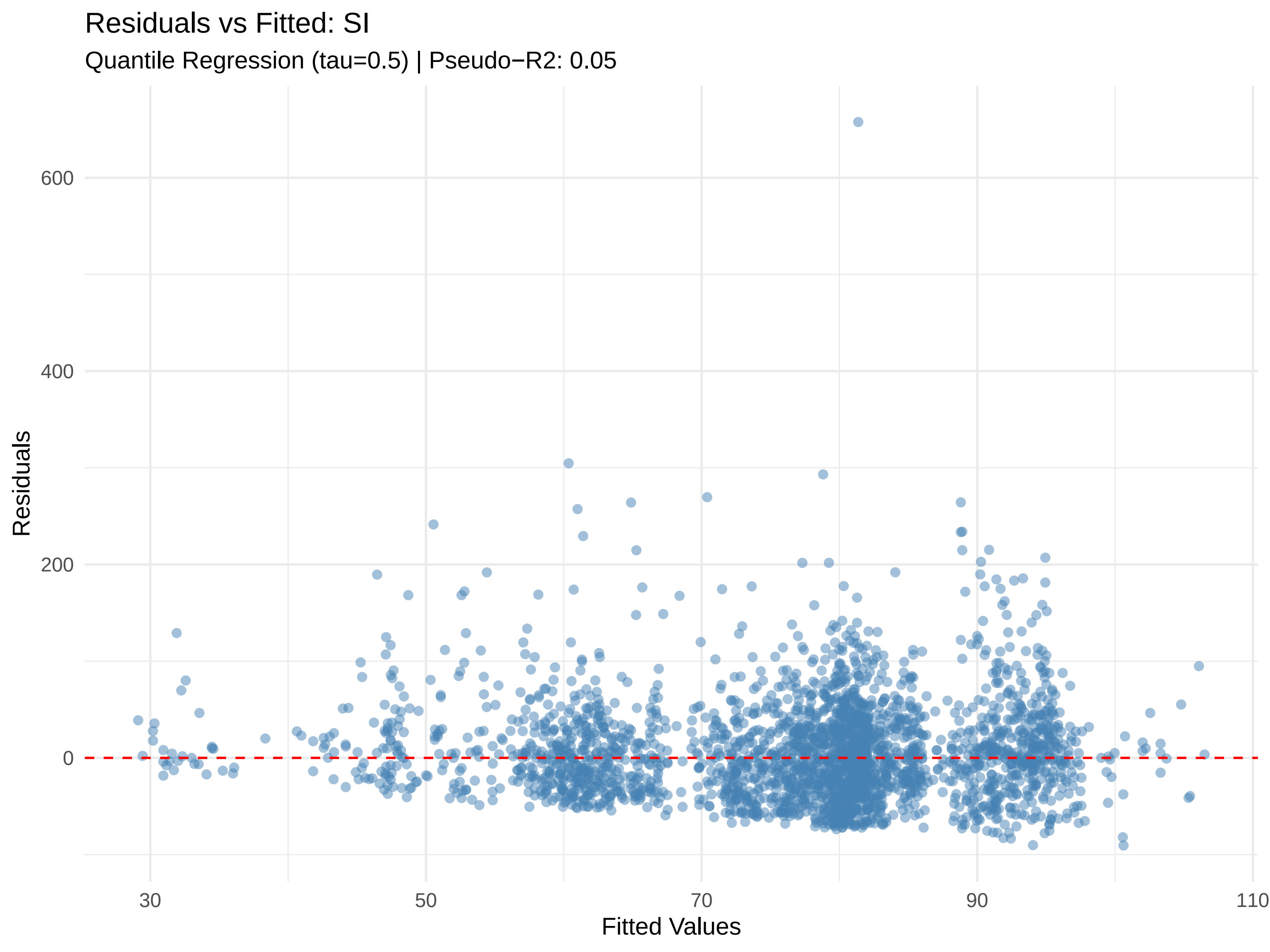

Residuals vs Fitted: SI
Quantile Regression (tau=0.5) | Pseudo−R2: 0.05
Residuals
600
400
200
0
30
50
70
90
110
Fitted Values

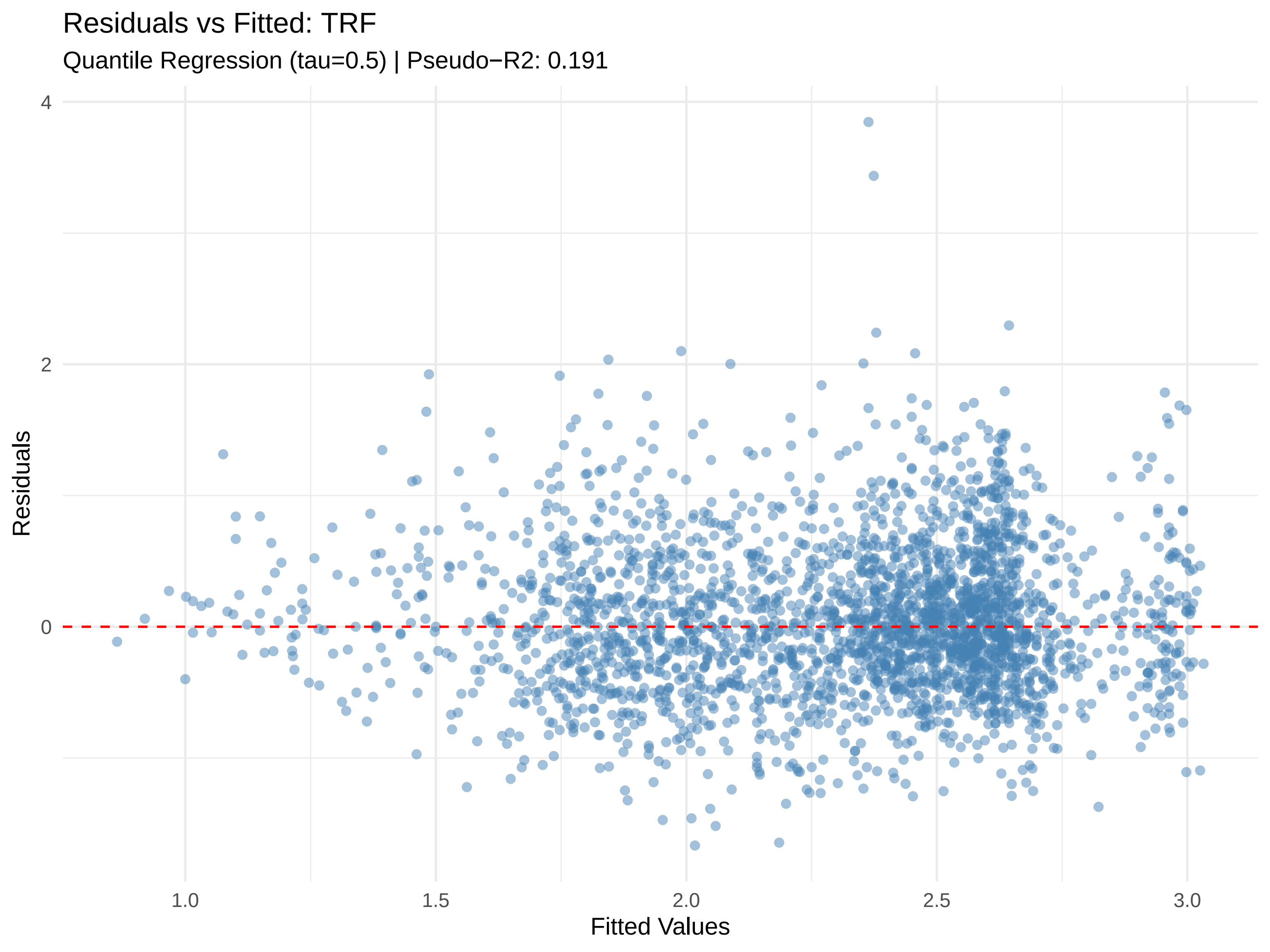
Residuals vs Fitted: TRF
Quantile Regression (tau=0.5) | Pseudo−R2: 0.191
Residuals
4
2
0
1.0
1.5
2.0
2.5
3.0
Fitted Values

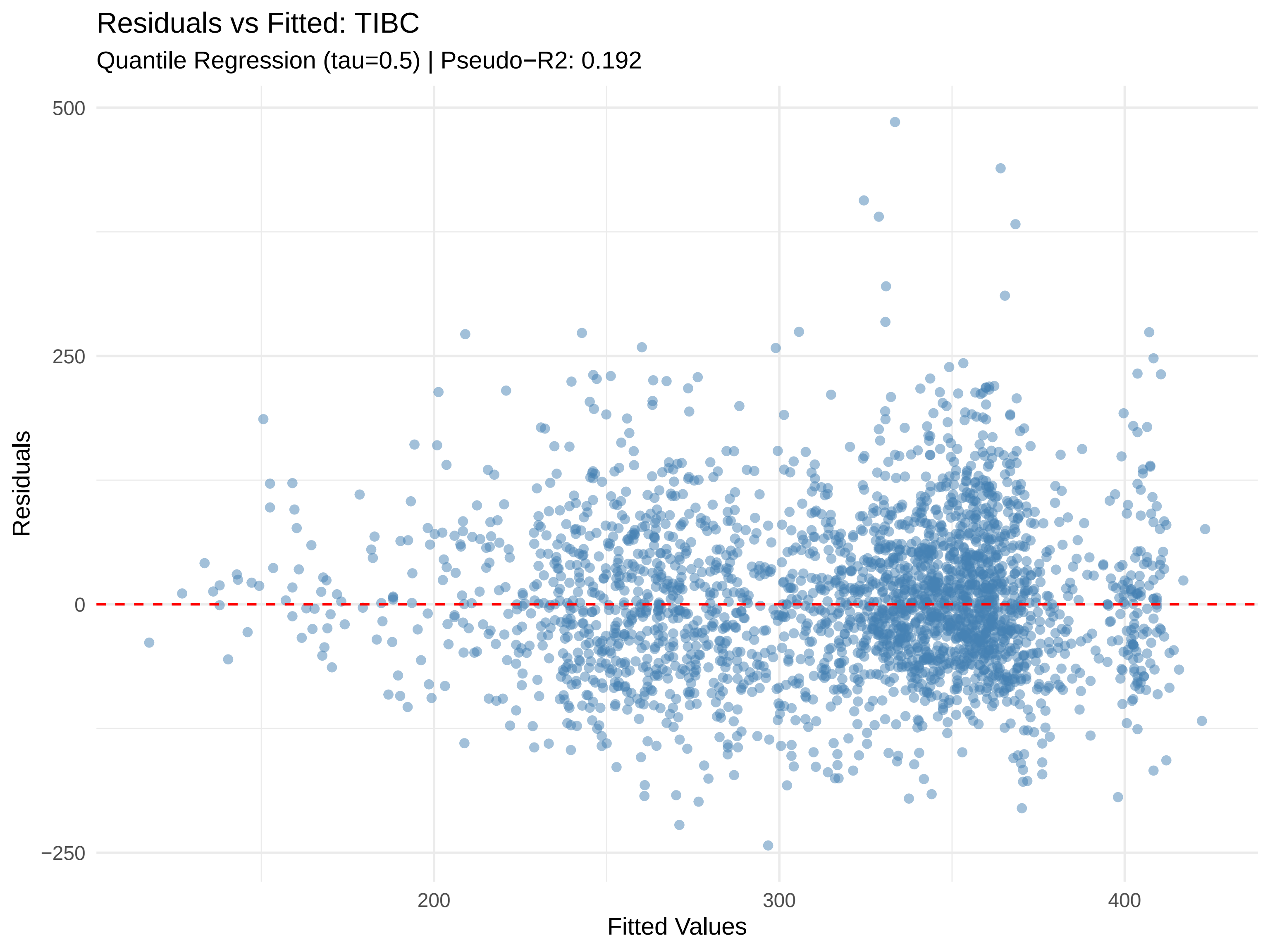
Residuals vs Fitted: TIBC
Quantile Regression (tau=0.5) | Pseudo−R2: 0.192
Residuals
500
250
0
−250
200
300
400
Fitted Values

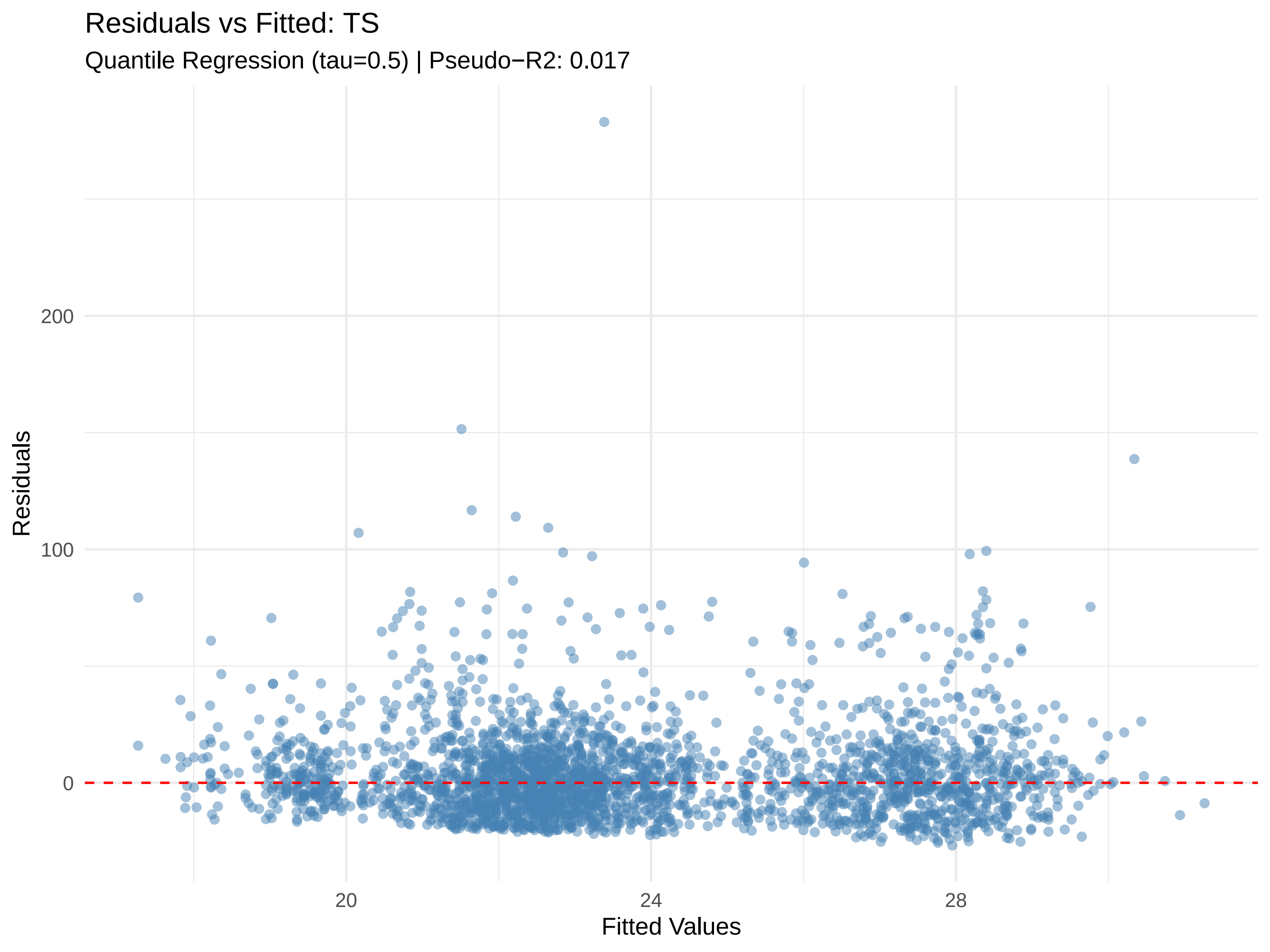
Residuals vs Fitted: TS
Quantile Regression (tau=0.5) | Pseudo−R2: 0.017
Residuals
200
100
0
20
24
28
Fitted Values

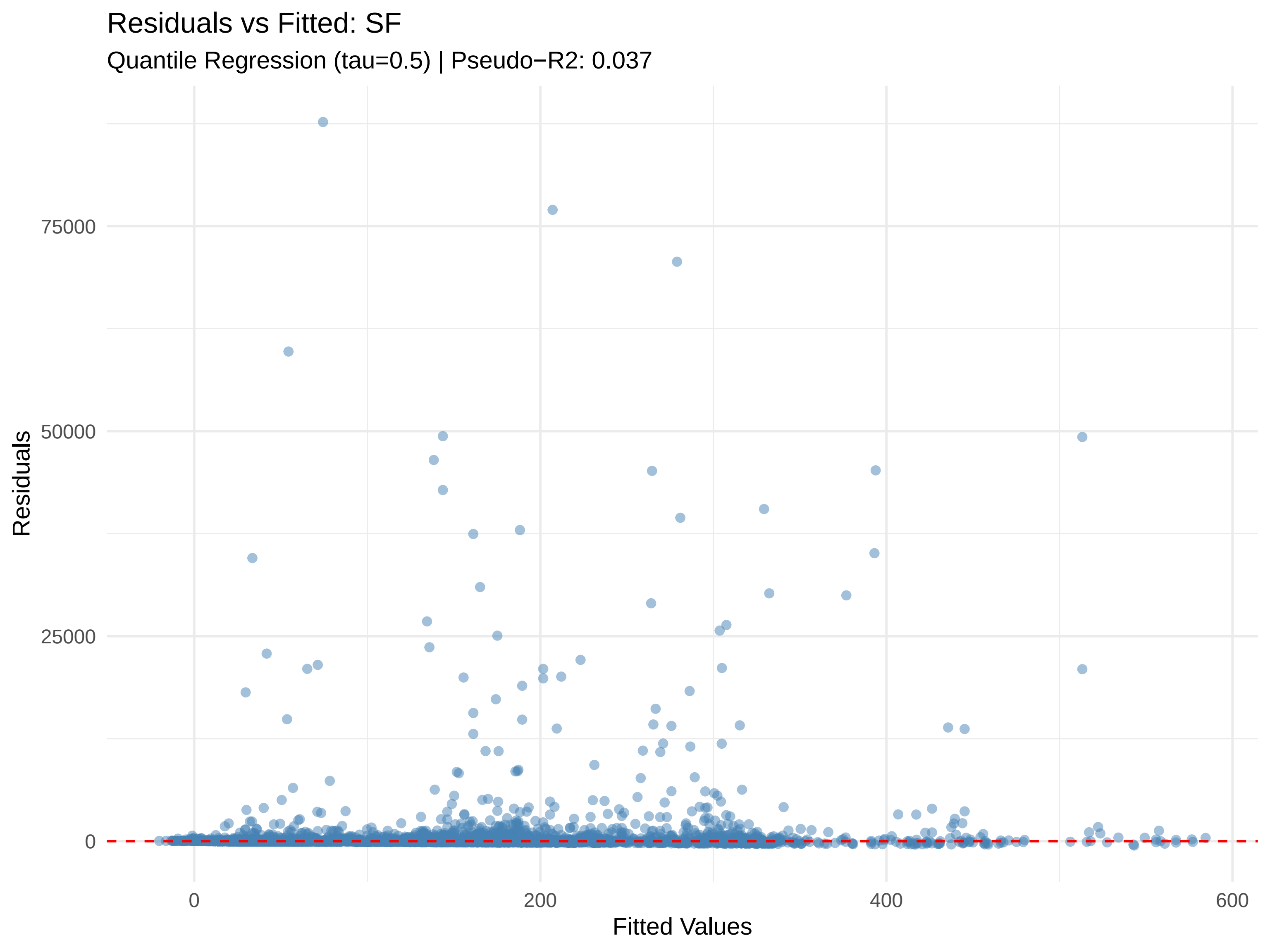

Residuals vs Fitted: SF
Quantile Regression (tau=0.5) | Pseudo−R2: 0.037
Residuals
75000
50000
25000
0
0
200
400
600
Fitted Values

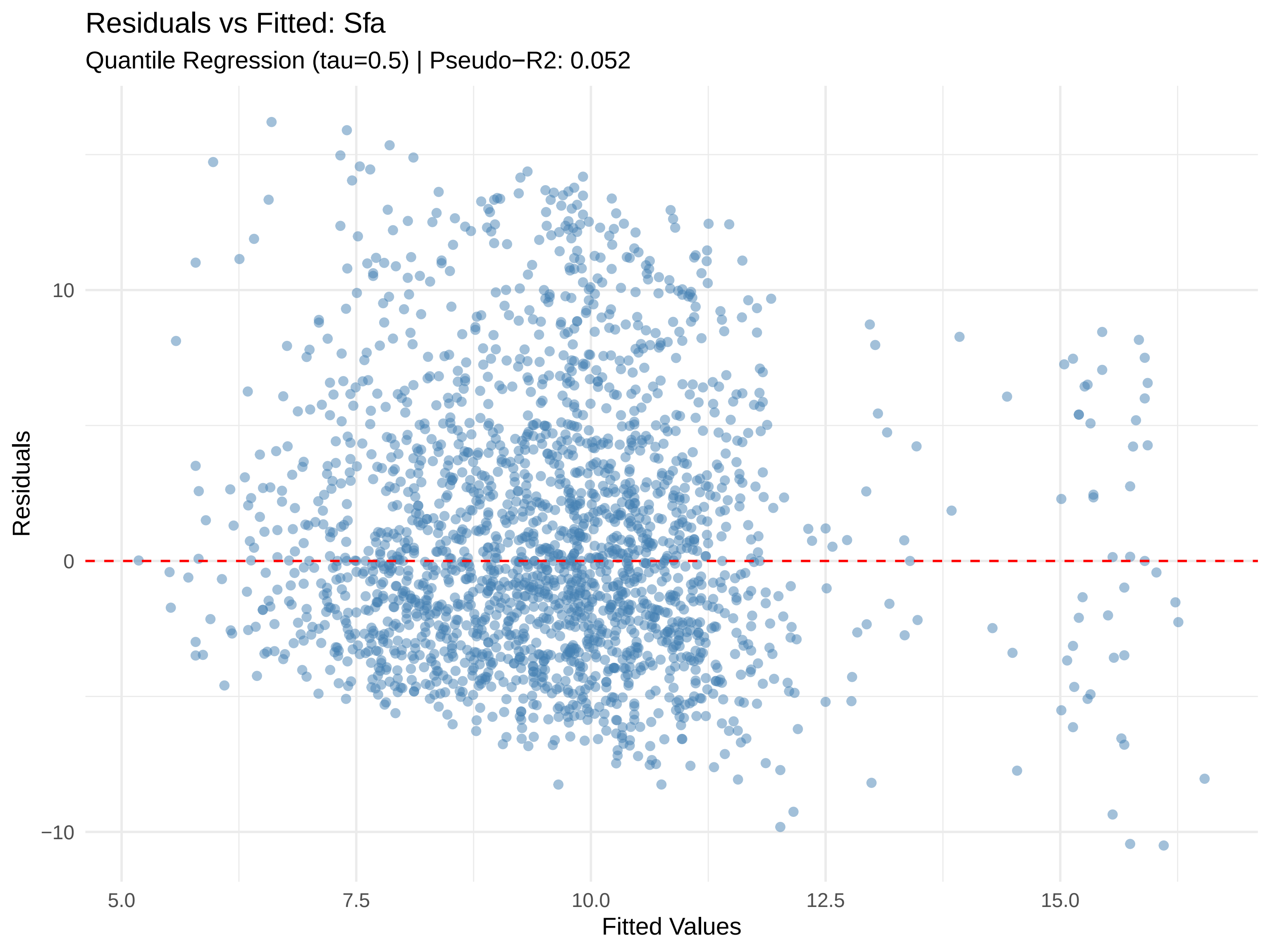

Residuals vs Fitted: Sfa
Quantile Regression (tau=0.5) | Pseudo−R2: 0.052
Residuals
10
0
−10
5.0
7.5
10.0
12.5
15.0
Fitted Values

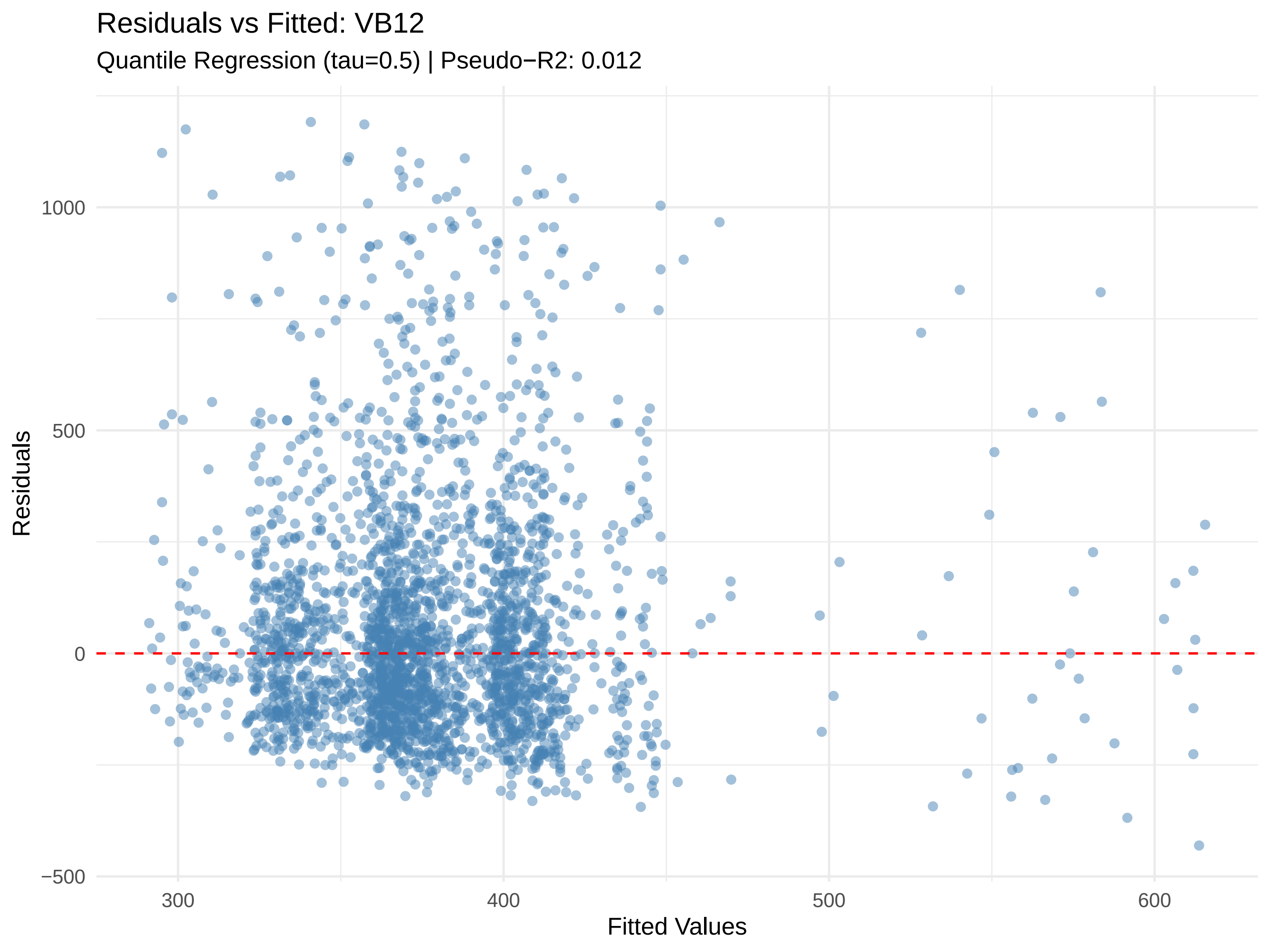

Residuals vs Fitted: VB12
Quantile Regression (tau=0.5) | Pseudo−R2: 0.012
Residuals
1000
500
0
−500
300
400
500
600
Fitted Values
